# Comprehensive Mendelian randomization and colocalization analysis of plasma proteomics to identify new therapeutic targets for bladder cancer

**DOI:** 10.7150/jca.116402

**Published:** 2025-07-11

**Authors:** Jieming Zuo, Junhao Chen, Zhiyong Tan, Lingxiang Wen, Junxian Zhao, Yuanzhi Fu, Haifeng Wang, Shi Fu, Jiansong Wang

**Affiliations:** 1 Yunnan Institute of Urology, The Second Affiliated Hospital of Kunming Medical University, Kunming 650101, China.; 2 Kunming Medical University, Kunming City, Yunnan Province, China.; 3 Department of Urology, 920th Hospital of Joint Logistics Support Force of Chinese People's Liberation Army, Kunming, Yunnan Province, China.; 4 Kunming University of Science and Technology, Kunming City, Yunnan Province, China.

**Keywords:** Bladder cancer, proteomics, circulating proteins, drug target.

## Abstract

Bladder cancer is characterized by a high recurrence rate and aggressive behavior, with frequent emergence of chemoresistance. Current treatments such as surgery, chemotherapy, and immunotherapy have limited efficacy, underscoring the urgent need for effective early diagnostic biomarkers and novel targeted therapies.

**Results:** In this study, we integrated plasma proteomic data from the UK Biobank Pharma Proteomics Project (UKB-PPP) and the Icelandic deCODE study with genome-wide association study (GWAS) data. We employed two-sample Mendelian randomization (MR), Bayesian colocalization analysis, and SMR/HEIDI tests to systematically identify potential plasma protein targets associated with bladder cancer risk. A total of 199 plasma proteins were found to be significantly associated with bladder cancer risk, among which five proteins (SLURP1, LY6D, WFDC1, NOV, and GSTM3) emerged as core candidate targets. Further validation showed that NOV and GSTM3 demonstrated robust causal associations with bladder cancer across multiple analytical methods, and molecular docking analysis revealed that these two proteins can bind to estrogen/progestin hormone-regulating drugs.

**Conclusions:** Our study identified multiple plasma proteins with causal links to bladder cancer and revealed their potential roles in tumor immune evasion, antioxidant defenses, and tumor metabolism. These findings provide new insights into bladder cancer biology and offer potential targets for precision therapy and drug repositioning.

## 1. Introduction

Bladder cancer (BC) is one of the most common malignancies of the urinary system worldwide, with over 500,000 new cases and nearly 200,000 deaths in 2020. Despite advances in surgery, chemotherapy, and immunotherapy for BC, its high recurrence rate, aggressive nature, and frequent development of chemotherapy resistance still limit therapeutic success 1. Early diagnosis and targeted therapy are crucial to improving the survival of BC patients; therefore, there is an urgent need to discover novel biomarkers and develop effective therapeutic targets. Recent innovative assays for bladder cancer detection have significantly advanced diagnostic capabilities. For instance, a novel urine-based DNA methylation test has demonstrated promise for early bladder cancer detection and recurrence monitoring. Studies have shown this multiplex urine DNA methylation assay to be highly sensitive and non-invasive, marking significant progress in diagnostic techniques 2, 3. Acknowledging these emerging diagnostic tools further emphasizes the need for novel biomarkers. In recent years, with the development of liquid biopsy techniques, proteins circulating in the blood-released via cell leakage or active secretion-are considered an important window into the disease state 4. These circulating proteins have potential as early diagnostic and prognostic biomarkers for bladder cancer, and may also serve as candidate therapeutic targets. For example, the protein haptoglobin has been found to be differentially expressed in the plasma of low-grade bladder cancer patients, suggesting its potential as an early screening biomarker 5. Another study has shown that large-scale plasma proteomics can identify apolipoprotein A1 as a potential diagnostic biomarker with high accuracy (AUC = 0.906), and implicate it in inflammatory signaling relevant to tumor progression 6.

Large-scale proteomic studies provide valuable datasets for identifying plasma proteins associated with bladder cancer. These datasets include thousands of protein quantitative trait loci (pQTLs). By applying Mendelian randomization (MR) analysis, we have an opportunity to reveal causal relationships between plasma proteins and bladder cancer risk 7. MR leverages naturally occurring genetic variation as an instrumental variable, which helps minimize reverse causation and confounding bias, thus allowing more reliable inference of the causal effect of proteins on disease risk. In recent years, MR approaches have achieved significant progress in studies of other diseases, providing new insights into the causal factors of stroke, diabetes, and cancers 8.

In this study, we performed a proteome-wide MR analysis by integrating bladder cancer GWAS summary data with plasma proteomic GWAS data, in order to identify plasma protein biomarkers significantly associated with bladder cancer risk. To strengthen the causal interpretation of our findings, we further combined colocalization analysis, the SMR (summary-data-based MR) method, and the HEIDI test to exclude biases. In addition, we evaluated the druggability of these proteins by in silico analysis to assess their prospects for clinical development as therapeutic targets. Our work provides a theoretical basis and translational possibilities for precision therapy in bladder cancer.

## 2. Methods

### 2.1 Data sources

We obtained two large-scale GWAS datasets of plasma protein levels from published studies. The first was from the UK Biobank Pharma Proteomics Project (UKB-PPP), which provides summary statistics for 2,940 plasma proteins measured in 54,219 participants using the Olink Explore 3072 proteomics platform 9. The second was from the deCODE study in Iceland, which reported GWAS results for 4,907 plasma proteins measured in 35,559 participants using the SomaScan v4 platform 10. These proteomic GWAS summary data are publicly available at the OpenGWAS/UKB-PPP repository (https://registry.opendata.aws/ukb-ppp/) and the deCODE summary data portal (https://www.decode.com/summarydata/).

Bladder cancer association data were obtained from a publicly available GWAS of bladder cancer in a Finnish population. This dataset (Finngen, release R11) includes 2,574 bladder cancer cases and 345,118 controls, all of Finnish ancestry, and was used as the outcome for our MR analyses 7. The summary statistics for this GWAS are accessible at finngen-public-data-r11/summary_stats/finngen_R11_C3_BLADDER_EXALLC.gz. All data utilized in this study were obtained from open public databases; therefore, no additional ethical approval was required.

### 2. 2 MR analysis

Genetic instruments for plasma protein levels were defined as cis-acting single nucleotide polymorphisms (SNPs) within a 1 Mb window of the gene encoding the protein, with an association p-value < 5 × 10^-8 for the protein level. Using two-sample MR analysis, we identified 2,080 and 1,922 independent cis-pQTL instruments for proteins from the UKB-PPP and deCODE datasets, respectively (After excluding proteins with no valid cis-pQTL instruments, we obtained instruments for 2,080 proteins in the Olink dataset and 1,922 proteins in the deCODE dataset). In the MR framework, the two proteomic GWAS datasets (UKB-PPP and deCODE) were treated as exposures, and two independent bladder cancer GWAS summary datasets were used as outcomes (including the Finnish dataset described above, and an additional GWAS). For each protein, if only a single genetic instrument was available, we used the Wald ratio to estimate the MR effect; if two or more instruments were available, we used the inverse-variance weighted (IVW) method. To test the robustness of the results and address potential heterogeneity or horizontal pleiotropy among instruments, we performed sensitivity analyses. In cases where Cochran's Q test indicated significant heterogeneity among the instruments, we applied the weighted median method. The weighted median MR method can provide a consistent causal effect estimate even if up to 50% of the instrument weight comes from invalid instruments, offering robustness against outliers or invalid variants. The MR analyses were conducted using the TwoSampleMR package (version 4.3.1) in R. We applied a false discovery rate (FDR) correction to adjust for multiple testing across the many proteins analyzed. By using an updated 1000 Genomes reference panel for clumping (provided by CTG-VL), we were able to include more SNPs per protein in each MR analysis, making the results more reliable than using the older reference panel.

### 2.3 Colocalization analysis

To further verify the causal relevance of the significant MR findings, we performed a Bayesian colocalization analysis 11. This analysis evaluates whether each locus contains a single genetic variant influencing both plasma protein level and bladder cancer risk, thereby distinguishing a truly shared causal variant from a coincidental co-occurrence due to linkage disequilibrium (LD). We implemented the colocalization analysis using the coloc R package (http://cran.r-project.org/web/packages/coloc) within a Bayesian framework. The method calculates posterior probabilities for five hypotheses: H0 (no association with either trait), H1 (association with trait 1 only), H2 (association with trait 2 only), H3 (association with both traits, but due to different variants), and H4 (association with both traits due to the same shared causal variant). We focused on the H4 scenario, which indicates that a protein level and bladder cancer risk are driven by the same genetic variant. We established criteria to classify potential target proteins based on the posterior probability for colocalization. Proteins with a posterior probability for hypothesis 4 (PP.H4) greater than 0.8 were considered primary targets with strong colocalization support. Proteins with 0.5 < PP.H4 ≤ 0.8 were classified as secondary targets with moderate support, and those with PP.H4 ≤ 0.5 were designated as tertiary targets with little support. However, recognizing the limitation of the basic colocalization approach in loci with multiple causal variants, we also adopted a combined posterior probability threshold. We deemed a gene locus to have strong evidence of colocalization if the sum of the posterior probabilities of H3 and H4 (PP.H3 + PP.H4) was ≥ 0.7. This threshold indicates a high overall probability of a shared genetic signal and has been validated in prior research 11. Using this criterion helps balance sensitivity and specificity, allowing us to screen for protein targets potentially having a causal relationship with bladder cancer risk while accounting for more complex genetic architectures.

### 2.4 SMR analysis

We conducted an SMR (summary-data-based Mendelian Randomization) analysis as complementary evidence to validate the causal relationships between proteins and bladder cancer. The SMR method integrates summary-level data from pQTL (protein QTL) and disease GWAS to test for causally associated traits. We used the SMR software developed by Zhu *et al.* and Wu *et al.* 12, 13, applying a Bonferroni-corrected significance threshold of P < 1.47 × 10^-3 (0.05/34, where 34 corresponds to the number of tests or candidates considered) for the SMR results. In addition, we employed the HEIDI (Heterogeneity in Dependent Instruments) test to evaluate whether an observed protein-disease association could be driven by linkage by multiple variants. In our criteria, an association was considered evidence of causality if the SMR test P-value was < 0.05 and the HEIDI test P-value was > 0.05. An SMR association with HEIDI P > 0.05 indicates no significant heterogeneity or pleiotropy, meaning the association is unlikely to be driven by multiple distinct variants in LD, thereby supporting the reliability of a single causal variant hypothesis.

### 2.5 PPI network and pathway enrichment analysis

To characterize the biological functions of the identified proteins, we performed gene ontology (GO) enrichment and Kyoto Encyclopedia of Genes and Genomes (KEGG) pathway enrichment analyses on the set of candidate target proteins. These analyses covered GO categories of molecular function, cellular component, and biological process, as well as relevant signaling and metabolic pathways. We used the ClusterProfiler R package 14 and the DAVID tool 15 for enrichment analysis, which are high-throughput functional annotation tools capable of statistical testing and interpretation of large proteomic datasets. Enrichment results with Benjamini-Hochberg adjusted P-value (p.adjust) < 0.05 were considered statistically significant. Simultaneously, we constructed a protein-protein interaction (PPI) network to reveal potential interactions among the identified proteins. PPI data were retrieved from the STRING database (https://string-db.org/), version 11.5, which integrates interaction information from experimental validation, computational prediction, and text mining. We visualized and analyzed the PPI network using Cytoscape software (version 3.9.1) 16. To assess the importance of key nodes in the network, we performed a centrality analysis using the CytoNCA plugin in Cytoscape^17^, calculating metrics such as degree centrality, betweenness centrality, and closeness centrality. These network analyses help identify core proteins and interaction modules related to bladder cancer biology.

### 2.6 Molecular docking and druggability analysis

To evaluate the feasibility of the identified proteins as potential drug targets (i.e., their “druggability”), we investigated known and predicted interactions between these proteins and small-molecule drugs. We utilized the DrugBank database 18 for comprehensive information on drugs, their targets, mechanisms, and molecular interactions, and the DSigDB database 19, which contains drug-associated gene sets and signature data. Through these resources, we screened for existing drugs or compounds related to the identified target proteins, compiling data on drug names, chemical structures, and development status, to explore their potential as therapeutic agents for bladder cancer. For the druggability analysis, we selected candidate small-molecule compounds from our screening results that had known direct or indirect relevance to bladder cancer, and we carried out molecular docking studies for those compounds with the core target proteins. The three-dimensional structures of the core target proteins (in PDB format) were obtained from the RCSB Protein Data Bank (https://www.rcsb.org/), and the chemical structures of small-molecule drugs (in SDF format) were retrieved from the PubChem database (https://pubchem.ncbi.nlm.nih.gov/). We performed molecular docking using the CB-Dock2 online tool 20, which improves blind docking by automatically identifying protein binding cavities, docking ligands, and applying homologous template fitting. In the docking simulations, a binding energy (estimated free energy of binding) less than 0 indicates that the ligand can bind to the protein spontaneously, and more negative binding energy values imply higher binding affinity. For each protein-drug pair, the docking grid (search space) was centered on the coordinates of the protein's known or predicted binding pocket (e.g., where a native ligand or key residue is located) to ensure the accuracy and biological relevance of the docking results.

## 3. Results

### 3.1 Plasma proteins associated with bladder cancer

**Figure 1:**
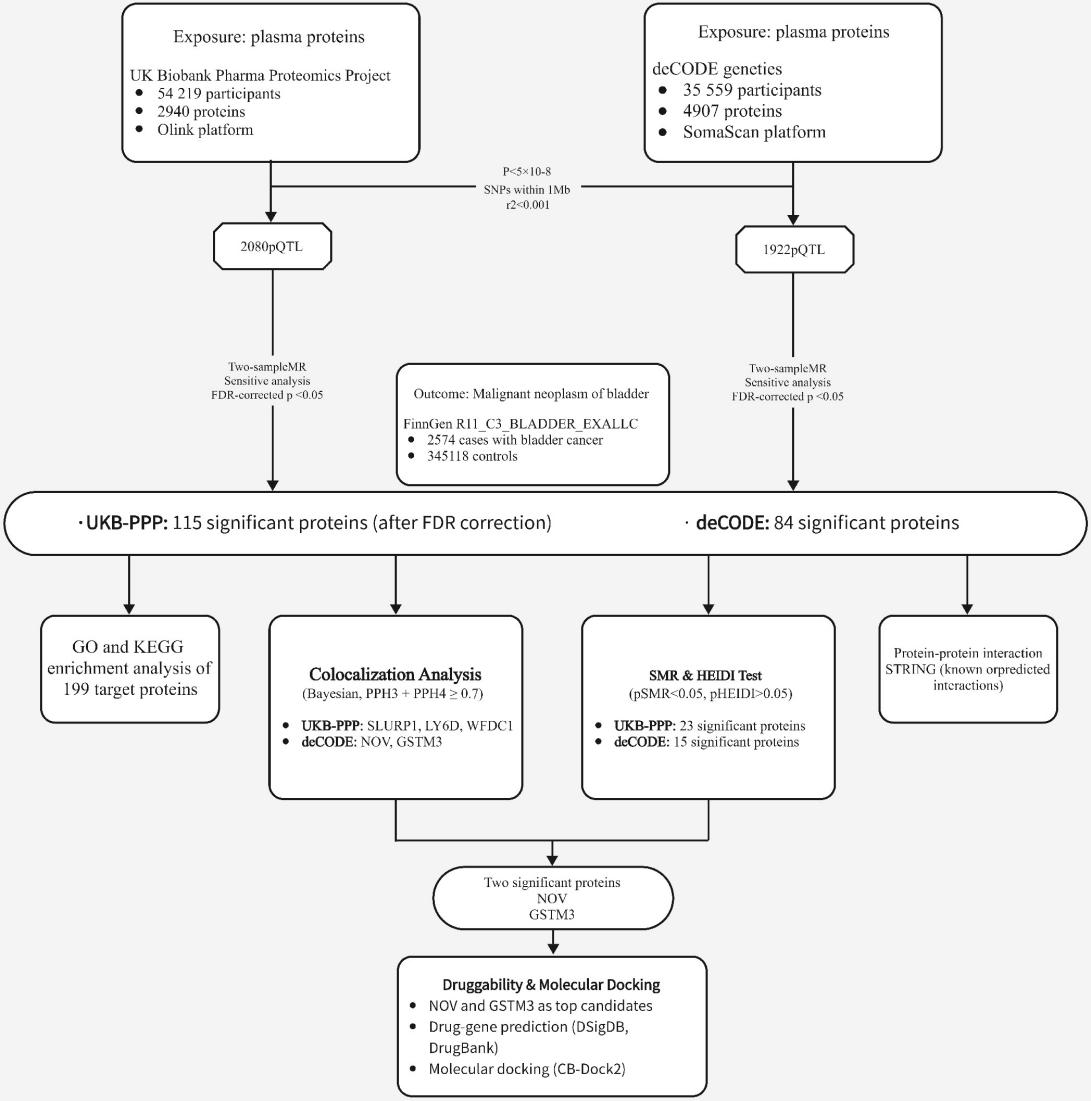
Flowchart of the study

**Figure 2:**
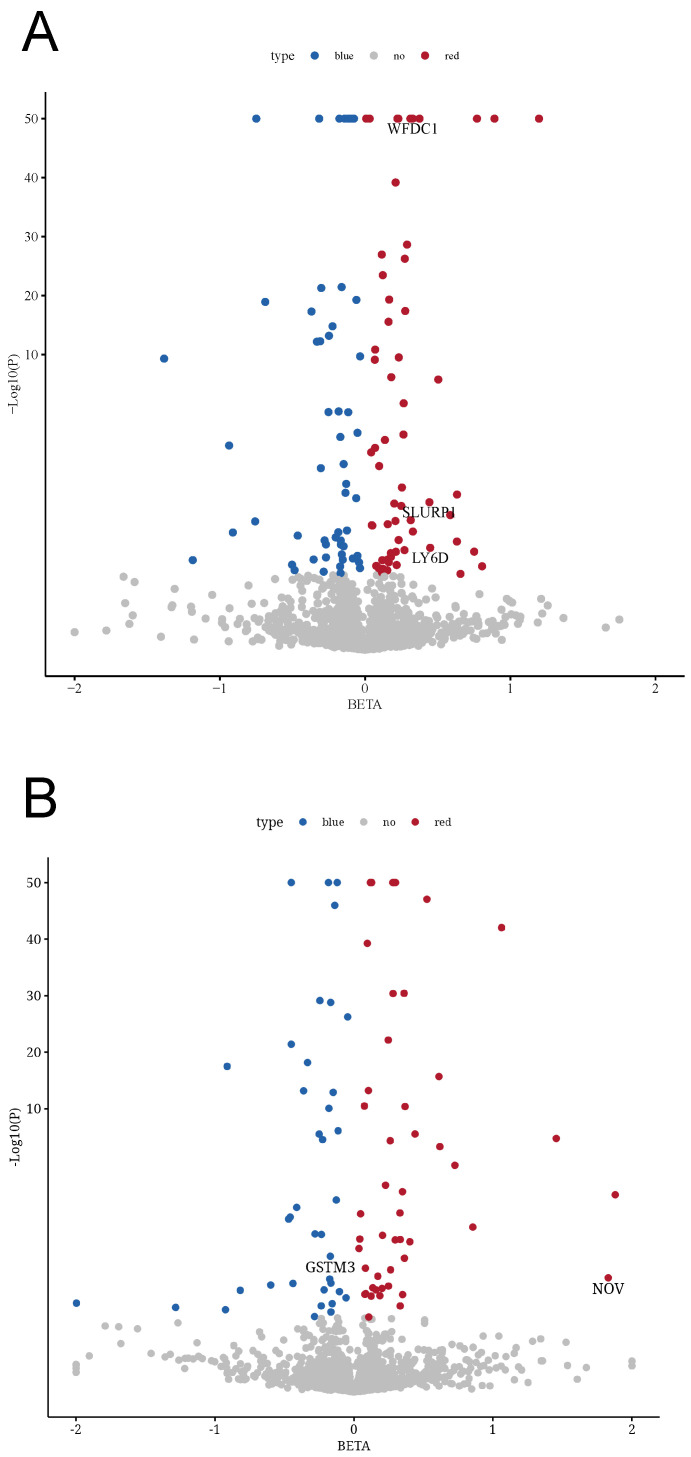
** Causal effects of the plasma proteins on BC. (A)** In the UKB-PPP study, 115 plasma proteins were significantly associated with bladder cancer (p < 0.05). **(B)** In the deCODE study, 84 plasma proteins were significantly associated with bladder cancer (p < 0.05). (Blue plots represented negative association, red plots represented positive association, while gray plots represented associations that did not reach the threshold of significance.).

An overview of our study design is shown in Figure 1 (analysis flowchart). To discover potential biomarkers for bladder cancer, we performed two-sample MR analyses to investigate the causal relationships between plasma protein levels and bladder cancer risk. We utilized two independent pQTL GWAS datasets: one from the Olink platform (UKB-PPP) and one from the deCODE proteomics database. After excluding plasma proteins without any valid genetic instrument, the MR analyses included 2,940 proteins from the Olink dataset and 4,907 proteins from the deCODE dataset. We obtained cis-pQTL instruments for 2,080 proteins in the Olink dataset and 1,922 proteins in the deCODE dataset, and carried out MR association tests for each protein with bladder cancer (applying FDR correction for multiple testing). In the Olink dataset, we identified 115 plasma proteins that showed a statistically significant association with bladder cancer risk (Figure 2A; adjusted p-values are provided in Supplementary Table S1). In the deCODE dataset, we identified 84 plasma proteins significantly associated with bladder cancer risk (Figure 2B; adjusted p-values in Supplementary Table S2).

### 3.2 Colocalization analysis identifies potential therapeutic targets

**Figure 3:**
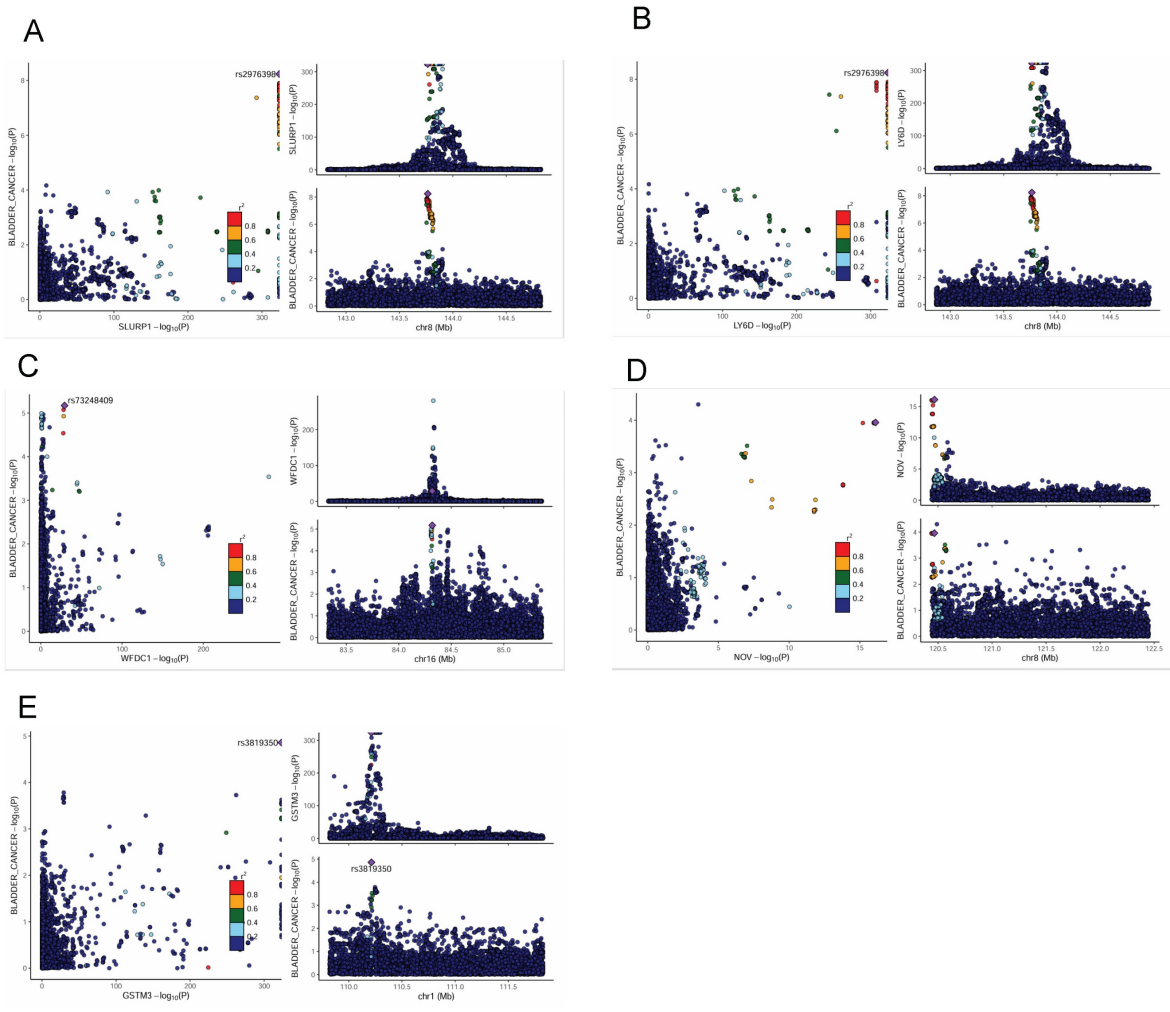
** Colocalization analysis of associations between plasma proteins and bladder cancer.** LocusCompare plots showing colocalization of SLURP1 **(A)**, LY6D **(B)**, WFDC1 **(C)** with bladder cancer susceptibility in the UKB-PPP study. LocusCompare plots showing colocalization of NOV (D), GSTM3 **(E)** with bladder cancer susceptibility in the deCODE study.

To further investigate the potential links between plasma proteins and bladder cancer, we performed a Bayesian colocalization analysis on the 199 plasma proteins filtered by the MR results from the two independent datasets (115 from Olink and 84 from deCODE). In this study, we defined strong colocalization evidence as a combined posterior probability (PP.H3 + PP.H4) ≥ 0.7. The colocalization analysis revealed that, among the 115 plasma proteins associated in the Olink dataset, three proteins - SLURP1, LY6D, and WFDC1 - exhibited strong evidence of colocalization with bladder cancer risk loci (PP.H3+PP.H4 values ~0.9999, 0.9998, and 0.758, respectively; Figure 3A-C, Supplementary Table S3). Similarly, among the 84 plasma proteins from the deCODE dataset, NOV and GSTM3 showed strong colocalization with bladder cancer risk (PP.H3+PP.H4 = 0.868 and 0.706, respectively; Figure 3D-E, Supplementary Table S4). These results suggest that SLURP1, LY6D, WFDC1, NOV, and GSTM3 are closely associated with the development of bladder cancer and have value as potential therapeutic targets, warranting further functional validation and clinical research.

### 3.3 SMR and HEIDI tests validate two causal proteins

**Figure 4:**
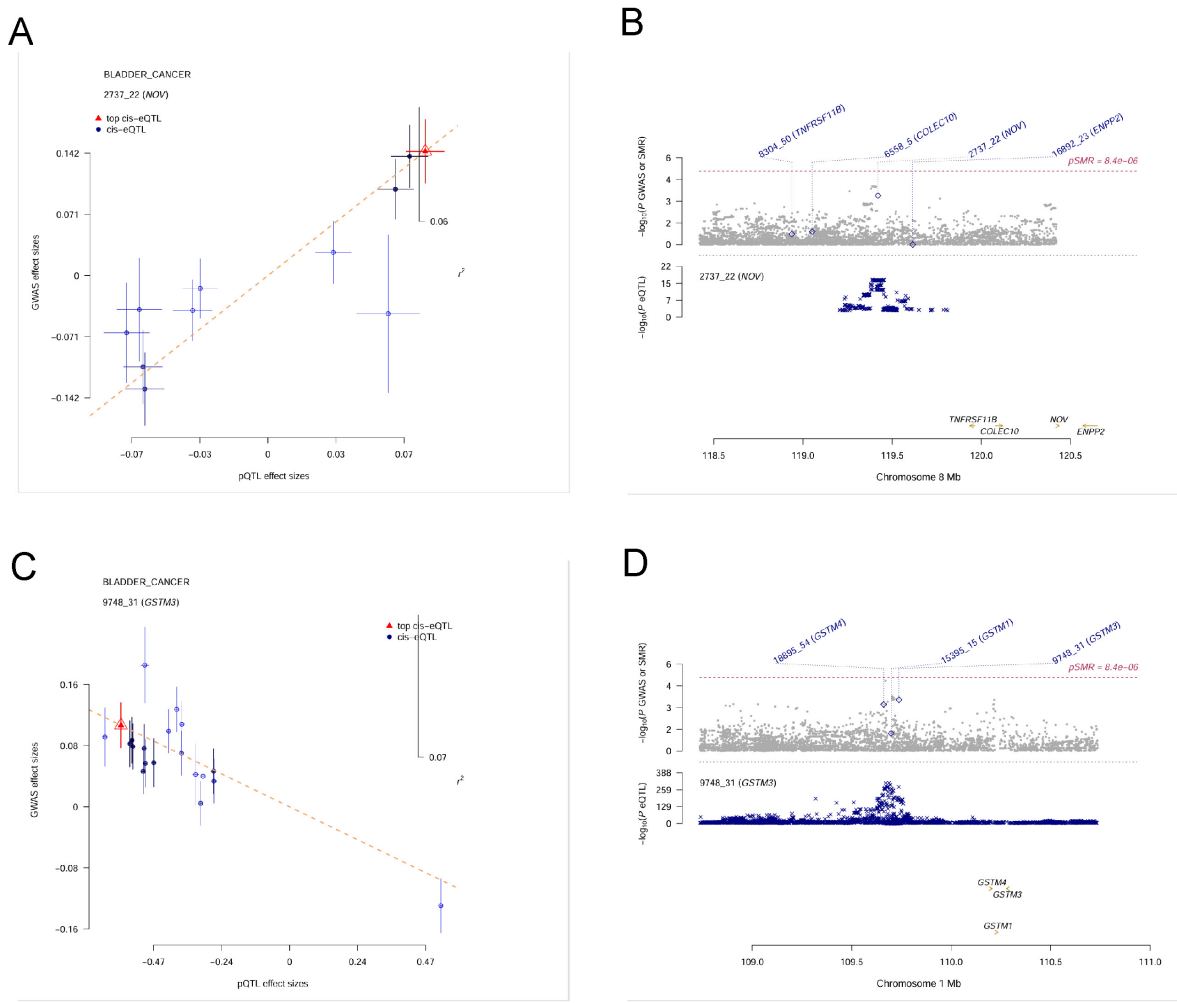
** Summary-data-based Mendelian Randomization (SMR) results for plasma proteins and bladder cancer risk. (A)**SMR effect plot for NOV. **(B)** SMR locus plot for NOV. **(C)** SMR effect plot for GSTM3. **(D)** SMR locus plot for GSTM3.

Because the colocalization approach assumes a single causal variant per locus and can be sensitive to complex LD patterns, potentially leading to bias in loci with multiple causal variants, we next applied the SMR method with HEIDI testing to validate the candidate proteins while accounting for pleiotropy or locus complexity. From the initial MR analysis, 115 plasma proteins were associated with bladder cancer in the Olink dataset; of these, 23 proteins showed evidence of association in the SMR analysis (P < 0.05) and also passed the HEIDI test (P > 0.05), as listed in Table 1. However, none of these 23 overlapped with the proteins identified by the colocalization analysis. In the deCODE dataset, 84 proteins were associated with bladder cancer by MR, of which 15 proteins passed the SMR threshold (P < 0.05) and HEIDI test (P > 0.05) (Table 2). Notably, NOV (Figure 4A, 4B) and GSTM3 (Figure 4C, 4D) were the two proteins that passed the colocalization analysis and also showed significant associations in SMR with no evidence of heterogeneity (HEIDI), indicating a high reliability in their causal associations with bladder cancer. These two proteins can be considered candidate pathogenic proteins, providing important clues for subsequent functional studies.

### 3.4 Protein-protein interaction and pathway enrichment analysis

**Figure 5:**
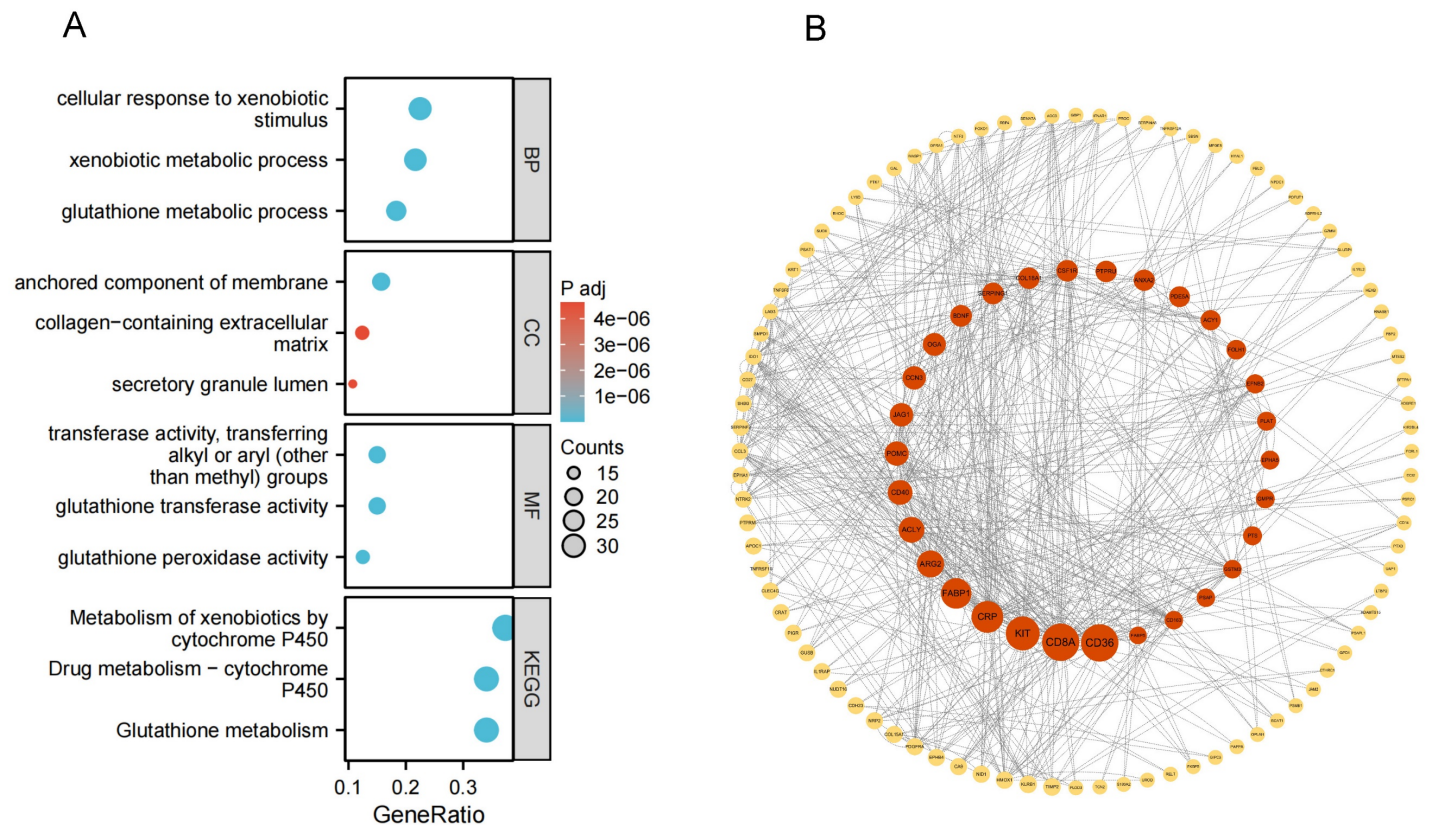
** GO, KEGG enrichment results, and PPI results. (A)** Enrichment results for 199 plasma proteins. **(B)** Protein-protein interaction (PPI) network for 199 plasma proteins (with Betweenness Centrality as a continuous variable).

To further elucidate the biological functions of these plasma protein targets, we performed Gene Ontology (GO) and KEGG pathway enrichment analyses for the combined set of 199 target proteins identified in the Olink and deCODE datasets. Using the clusterProfiler package in R, we identified numerous significantly enriched GO terms and pathways (with Benjamini-Hochberg adjusted P < 0.05). The enriched pathways indicate that these targets are closely related to tumor glutathione metabolism, xenobiotic metabolic processes, cytochrome P450 enzyme system, anchored component of membrane, and glutathione transferase activity (Figure 5A). We next constructed a PPI network of the 199 plasma proteins using the STRING database (v11.5), applying a medium confidence threshold for interactions (score ≥ 0.4; see Supplementary Figure S1). The PPI network was visualized in Cytoscape and comprised 189 nodes and 307 interaction edges (P < 0.001 by network randomization; Figure 5B). We then performed centrality analysis using the CytoNCA plugin to identify key nodes within this network. By examining degree, betweenness, and closeness centralities, we could pinpoint the core proteins in the network and their interaction patterns. These core proteins potentially play important roles in bladder cancer's biological mechanisms and may represent central hubs in pathways of interest.

### 3.5 Therapeutic target druggability evaluation via molecular docking

**Figure 6:**
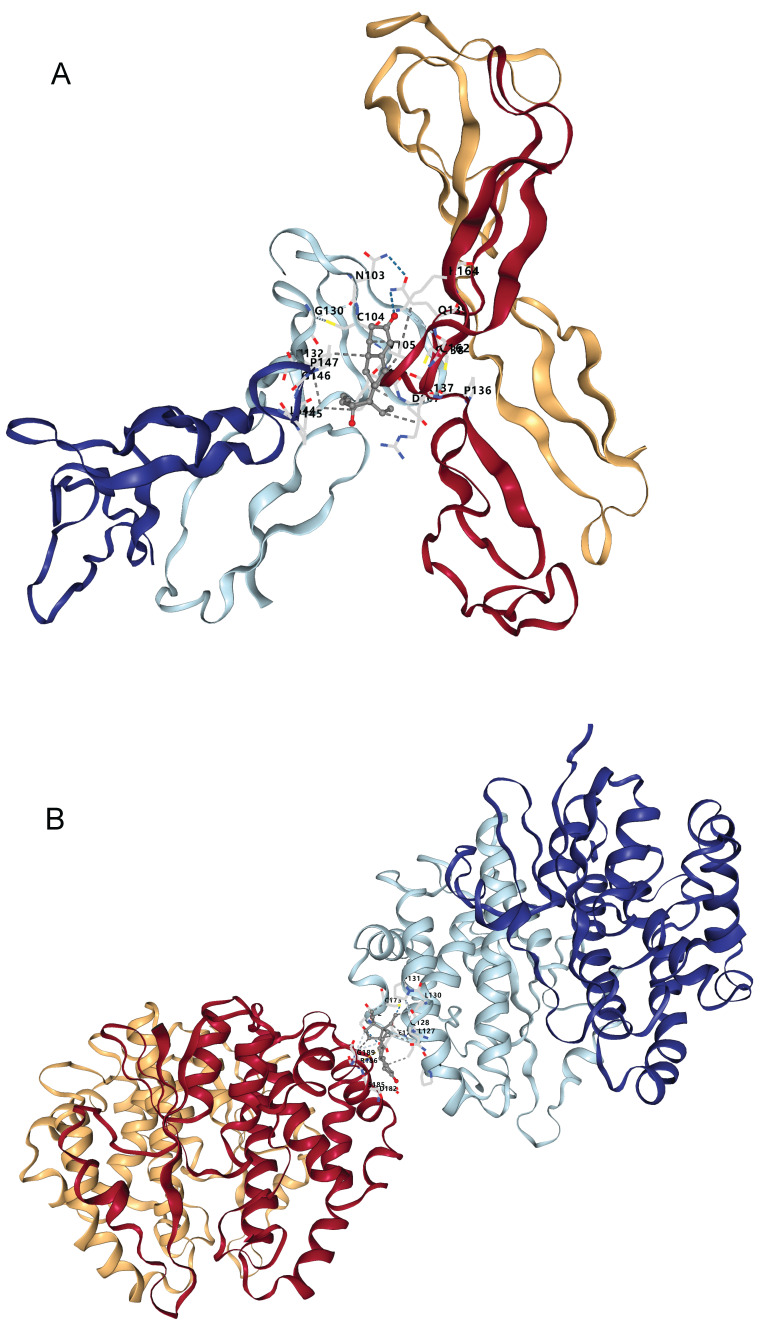
** Docking results of available proteins with small molecule ligands. (A)** Results of NOV docking with Norgestrel. **(B)** Results of GSTM3 docking with Medroxyprogesterone Acetate.

Through the above analyses, we identified two plasma protein targets - NOV and GSTM3 - that are highly relevant to bladder cancer. We then explored potential therapeutic compounds targeting these proteins. We queried the DSigDB database to retrieve drugs or small molecules whose gene expression signatures matched those of NOV or GSTM3. In the Comparative Toxicogenomics Database (CTD), we found 19 drugs or chemical substances that could potentially regulate or affect NOV expression (see Table 3), and 34 drugs or compounds that might modulate GSTM3 expression (Table 4). Interestingly, both targets were found to be potentially regulated by estrogen/progesterone (hormonal) drugs. We next downloaded the three-dimensional structures of the core target proteins and the identified candidate drugs from the PDB and PubChem databases, respectively, and used CB-Dock2 to predict the binding interactions between these drugs and targets. The molecular docking results showed that norgestrel (a progestin) has multiple potential binding sites on the NOV protein (Figure 6A, Table 5). Among these, binding pocket C1 demonstrated the highest binding affinity for norgestrel, with a Vina score of -7.7 kcal/mol (pocket volume 1071 Å, center coordinates at (8, 5, 6), pocket dimensions 21×21×21 Å). This result suggests that pocket C1 may serve as the primary binding site for norgestrel on the NOV protein. Similarly, medroxyprogesterone acetate (MPA) showed multiple binding sites on GSTM3 (Figure 6B, Table 6), with the top-ranked pocket (C1) exhibiting the highest affinity (Vina score = -8.5 kcal/mol; pocket volume 2456 Å, center at (-10, -22, 83), pocket size 31×21×21 Å). These docking findings indicate that MPA binds strongly to GSTM3, with pocket C1 likely being the principal binding site on the protein.

## 4. Discussion

Bladder cancer is a common malignancy of the urinary system worldwide, characterized by high recurrence and aggressiveness. Traditional treatment modalities, including surgery, chemotherapy, and radiotherapy, have yielded limited success due to the high recurrence rate and the development of chemotherapy resistance in bladder cancer. Therefore, exploring new molecular targets is of great significance for understanding the tumorigenic mechanisms of bladder cancer and developing precise targeted therapies. Using proteomics data from the Olink and deCODE databases, our large-scale two-sample MR study identified 199 potential target proteins associated with bladder cancer progression and prognosis. These targets are closely involved in pathways such as glutathione metabolism, xenobiotic metabolic processes, cytochrome P450 enzyme activity, membrane anchoring, and glutathione transferase activity 21-25. Subsequent colocalization analysis highlighted SLURP1, LY6D, WFDC1, NOV, and GSTM3 as being closely linked to bladder cancer occurrence. Because the results for a few targets appeared to contradict their known biological functions, we further conducted SMR analysis for verification, which ultimately narrowed down NOV and GSTM3 as the two robust targets. Investigating these targets will help to deepen our understanding of the mechanisms of bladder cancer development and provide support for developing more effective targeted therapies 26. By analyzing the roles of these targets, our study offers a molecular-level insight into bladder cancer progression and can drive the development of innovative treatments. Further research and validation of these targets not only promise to improve the clinical outcomes of bladder cancer, but may also introduce new biomarkers and therapeutic options for early diagnosis, patient stratification, and prognosis evaluation in bladder cancer.

In the Olink dataset, we obtained three compelling protein targets through large-scale MR and colocalization analysis: SLURP1, LY6D, and WFDC1. SLURP1 and LY6D both belong to the lymphocyte antigen 6 (Ly6) superfamily, which typically play roles in immune responses, and they may influence the tumor microenvironment by modulating immune evasion mechanisms of bladder cancer cells 27. LY6D is thought to play a role in multiple tumor types, including bladder cancer, breast cancer, and other epithelial-origin cancers. One study that analyzed gene expression data of Ly6 family members found that several proteins, including LY6D, are overexpressed across various cancers, and notably, high LY6D expression is associated with poorer survival in patients with bladder cancer, ovarian cancer, and breast cancer 28. This finding aligns with our MR result for LY6D, which yielded a positive effect estimate (β = 0.4485), indicating that higher LY6D expression is positively correlated with increased bladder cancer risk. Meanwhile, the colocalization probability (PP.H3+PP.H4 > 0.7) in our analysis indicates that the LY6D expression trait and bladder cancer risk share a common genetic locus, further strengthening the evidence for LY6D's involvement in bladder cancer. The colocalization finding provides genetic-level support for LY6D as a potential target. Other research has shown that LY6D is highly expressed specifically in tumors with squamous differentiation, with higher expression in low-grade, non-muscle-invasive bladder cancers and lower expression in more aggressive muscle-invasive bladder cancers 29. This expression pattern suggests that LY6D could serve as a potential differentiation marker, particularly useful for identifying squamous differentiation features of bladder tumors and for prognostic evaluation of low-invasiveness tumors. Members of the Ly6 family are also believed to play important roles in immune regulation, potentially helping bladder cancer cells evade immune surveillance by affecting how the immune system recognizes them 30. We propose that LY6D may contribute to immune evasion in bladder cancer by modulating the activity of immunosuppressive cells in the tumor microenvironment, such as myeloid-derived suppressor cells (MDSCs) and regulatory T cells (Tregs), thereby dampening the cytotoxic function of CD8^+ T cells and reducing antitumor immune responsiveness.

Studies have found that inhibiting MDSCs or Tregs can enhance antitumor immunity, and LY6D may activate the PI3K/Akt and MAPK signaling pathways, which inhibit apoptosis and enhance immune evasion; indeed, PI3K/Akt signaling has been shown to promote PD-L1 expression in multiple cancer types 28, 30. These observations suggest that LY6D could play a key role in the immune evasion mechanisms of bladder cancer, making it a potential target for immunotherapy interventions.

The Ly6 family member SLURP1 is an endogenous ligand for nicotinic acetylcholine receptors (nAChRs), especially the α7 subunit. Studies indicate that SLURP1 expression is high in normal epithelial tissues but is significantly down-regulated in neoplastic (cancerous) tissues 31. Binding of SLURP1 to the α7-nAChR triggers a cascade of intracellular signaling events, including upregulation of NF-κB via both Ca^2+-dependent and Ca^2+-independent pathways. Specifically, this pathway involves activation of Raf-1/MEK1/ERK1/2 and the synergistic action of CaMKII (calcium/calmodulin-dependent protein kinase II) and protein kinase C (PKC) 28, 32-34. NF-κB is a crucial regulator of cell proliferation and survival, and through this pathway SLURP1 may contribute to the anti-apoptotic and proliferative processes in bladder cancer cells. Our findings suggest that SLURP1 and LY6D together may promote bladder tumor growth and progression via mechanisms involving immune evasion, antioxidant stress responses, and anti-apoptosis. Investigating the interplay between these factors could provide new targets and strategies for precision therapy and immunotherapy of bladder cancer. For instance, SLURP1 has been shown to reduce TNF-α-induced NF-κB activation, thereby suppressing inflammatory responses an anti-inflammatory function that might be important in the bladder cancer microenvironment and SLURP1 can also stabilize epithelial cell junctions 31. Meanwhile, LY6D might influence bladder cancer progression through interactions with non-coding RNAs or other proteins, complex mechanisms that may not be captured by traditional SMR analysis 27. Taken together, SLURP1 and LY6D likely contribute to bladder cancer through indirect effects on the tumor microenvironment and immune regulation. Their high-level influence on the tumor's interaction with the host immune system suggests that further study of these targets could yield novel therapeutic approaches, such as enhancing anti-tumor immunity by targeting LY6D or restoring SLURP1 function.

Our MR and colocalization analyses from the Olink dataset also highlighted WFDC1 as a potential bladder cancer target (meeting the PP.H3+H4 ≥ 0.7 criterion). WFDC1 (also known as ps20) is associated with tumor growth suppression and metastasis inhibition. As a protease inhibitor, WFDC1 can suppress the activity of certain cancer-related proteases that play important roles in tumor cell migration and invasion by degrading the extracellular matrix (ECM). When WFDC1 expression is down-regulated, those proteases become more active, potentially promoting tumor cells to invade surrounding tissues through enhanced ECM degradation 35. Additionally, WFDC1 has been found to be significantly down-regulated in cancer-associated fibroblasts (CAFs), which are cells in the tumor microenvironment that contribute to cancer development and progression 36. CAFs can secrete cytokines that promote tumor growth and metastasis and remodel the ECM to support cancer cell survival. By reducing the secretion of these pro-tumor factors, WFDC1 helps maintain the stability of the tumor microenvironment and inhibits tumor progression. Experimental studies have shown that overexpression of WFDC1 can markedly slow tumor cell growth, and interestingly, WFDC1 is located on chromosomal region 16q24 - a locus known to undergo loss of heterozygosity (LOH) in multiple cancer types. This suggests that WFDC1 plays a protective role in tumorigenesis, potentially by maintaining genomic stability and intercellular signaling 36, 37. However, in our two-sample MR analysis, the effect estimate for WFDC1 on bladder cancer risk was positive (approximately β = 0.328), implying that genetically higher WFDC1 levels were associated with increased risk of bladder cancer - a result seemingly at odds with its classical tumor-suppressive function. After applying FDR correction, WFDC1 remained a notable finding. We considered that the existing research on WFDC1 is relatively limited and largely based on *in vitro* functional experiments that emphasize its cancer-inhibiting effects. Such experiments, conducted in highly controlled environments, might overlook complex interactions present in living organisms. MR analysis, on the other hand, infers causality using genetic instruments and reflects the overall effect of genetically predicted expression (or protein level) on the phenotype (bladder cancer) in a population, rather than a direct functional effect observed in cell-based systems. It is also known from genome-wide association studies that the majority of bladder cancer-associated genetic variants reside in non-coding regions, which can influence gene expression through regulatory networks 38, 39.

Our colocalization analysis indicates that genetic variants in the WFDC1 locus may play a key role in bladder cancer risk. It is possible that certain genetic variants affecting WFDC1 expression or function could, in specific environmental or tissue contexts, make this gene act as a cancer-promoting factor rather than a suppressor. This consideration leads us to the hypothesis of “dual roles” for genes or proteins: in the complex etiology of diseases, the same gene can have context-dependent opposite effects (protective in one context but risk-promoting in another) 40. There are precedents for such dual roles; for example, some dual-specificity phosphatases (DUSP family) can either suppress or promote cancer depending on context 41, protein misfolding can have gain- or loss-of-function consequences in neurodegenerative diseases 42, and certain microbial pathogen effector proteins can both facilitate infection and trigger plant immune resistance 43. We speculate that the bladder tumor microenvironment may specifically alter the biological effect of WFDC1. In the context of bladder cancer, WFDC1 might paradoxically promote tumor invasion, possibly by enhancing protease activity or altering inflammatory regulation, despite its tumor-suppressive role observed in other settings. Further research is needed to explore WFDC1's role under different conditions and to test the dual-function hypothesis-for instance, whether WFDC1 might have pro-tumorigenic effects when influenced by certain regulatory variants or microenvironment factors in bladder tissue.

To obtain more comprehensive evidence of causality and function for this complex disease, we integrated the bladder cancer data with SMR analysis for validation, using the HEIDI test as a critical complement, and incorporated the deCODE proteomic data as well. Colocalization and SMR/HEIDI offer complementary approaches - the former provides Bayesian evidence of a shared variant, whereas the latter confirms causality with a specific instrument while detecting pleiotropy. We used both to ensure robust identification of causal protein targets. This step further ensured the reliability of our findings and reduced the likelihood of pleiotropy or heterogeneity affecting the results, thereby providing stronger support for identifying true bladder cancer targets. Through this validation step, SLURP1, LY6D, and WFDC1 did not pass the stringent SMR/HEIDI criteria, whereas NOV and GSTM3 passed all tests. An SMR result with HEIDI P > 0.05 effectively rules out substantial pleiotropy or heterogeneity, meaning the association is likely driven by a single genetic variant, and thus strengthens the evidence for a causal relationship. The fact that NOV and GSTM3 showed consistent associations across MR, colocalization, and SMR (with negative HEIDI) suggests that their gene expression levels may influence bladder cancer risk through a direct and singular pathway. Therefore, NOV and GSTM3 can be prioritized as bladder cancer biomarkers or therapeutic targets for in-depth research. On the other hand, our findings indicate that the genes SLURP1, LY6D, and WFDC1 may influence bladder cancer development through more indirect or context-specific pathways, rather than through direct causal mechanisms detectable by MR alone. Both SLURP1 and LY6D notably modulate the tumor microenvironment and immune responses, mechanisms that typically exert indirect impacts on tumor progression. For instance, SLURP1 is known to mitigate inflammation by inhibiting TNF-α-induced NF-κB activation within the microenvironment 44, and it helps preserve epithelial integrity by stabilizing cellular junctions 31. Similarly, LY6D may impact bladder cancer progression indirectly through complex interactions involving non-coding RNAs or protein complexes, nuances that single-variant-focused methods like SMR analysis might miss 27, 45. Similarly, the functions of WFDC1 appear to involve indirect regulation of the tumor microenvironment and intricate signaling pathways 36, 46, 47. Moreover, WFDC1 expression levels can vary significantly among different tissues, suggesting the observed eQTL signals for WFDC1 in bladder tissue could be context-dependent or subtle. Thus, our findings suggest future studies should explore WFDC1 interactions within broader cancer-related pathways, possibly incorporating higher-resolution datasets or integrated multi-omics approaches to better capture its context-specific roles in bladder cancer.

NOV (also known as CCN3) is a member of the CCN gene family of matricellular proteins. It encodes a secreted protein involved in numerous biological processes including cell adhesion, migration, proliferation, and apoptosis, and it plays important roles in tumorigenesis and cancer progression 48-50. Studies have shown that NOV can promote epithelial-mesenchymal transition (EMT): it increases tumor cell invasiveness and metastatic ability by down-regulating E-cadherin and up-regulating vimentin 51. Additionally, upregulation of NOV is associated with increased mTOR activity, highlighting its role in cancer metabolic reprogramming and proliferation 52. Our two-sample MR analysis also confirmed NOV as a promoting factor for bladder cancer (i.e., genetically higher NOV levels increase bladder cancer risk). Based on various lines of evidence, we speculate that NOV may promote bladder cancer progression by activating signaling pathways such as PI3K/AKT and Smad (TGF-β) to induce EMT and enhance the metastatic potential of bladder cancer cells 53, 54. Therefore, measuring NOV levels alongside EMT markers could potentially improve risk stratification and management of bladder cancer patients - for instance, identifying patients with a more aggressive, EMT-associated disease course who might benefit from therapies targeting these pathways. GSTM3 is a member of the glutathione S-transferase Mu (GST) family of enzymes. Its primary function is detoxification metabolism: it conjugates glutathione (GSH) to a wide variety of endogenous and exogenous toxins, aiding in their neutralization and elimination 55. Recent research on GSTM3 in cancers and other diseases indicates that it has significant effects on individual susceptibility, disease progression, and response to therapy (including chemotherapy) 56-58. One study demonstrated an interaction between GSTM3 deletion and smoking exposure: individuals with a GSTM3 deletion who smoke accumulate higher levels of carcinogens and DNA damage, which substantially increases their risk of bladder cancer 59. This is because individuals carrying mutations or deletions in the GSTM3 gene have a reduced capacity to detoxify carcinogens (such as tobacco-derived toxins and arylamines) 60. Such findings suggest that GSTM3 may act as a protective factor in bladder cancer, and our MR results are consistent with this - indicating that higher GSTM3 expression is associated with lower bladder cancer risk (a protective effect). In pancreatic and breast cancers, GSTM3 upregulation has been shown to significantly reduce reactive oxygen species (ROS) accumulation and simultaneously inhibit tumor cell glycolysis, altering the metabolic phenotype of the tumor cells. Through these actions, GSTM3 can modulate the energy supply of tumor cells and potentially inhibit tumor progression 61, 62. A similar mechanism likely applies to bladder cancer, where GSTM3's detoxification and antioxidant functions might suppress tumor growth by mitigating oxidative stress and metabolic reprogramming. In the context of cancer treatment, the role of GSTM3 is gaining attention. For example, in cervical cancer, GSTM3 has been reported to regulate the NF-κB and MAPK signaling pathways, playing a crucial role in tumor cell survival, proliferation, and anti-apoptotic functions. These pathways not only drive tumor cell growth but are also closely related to the cancer cells' resistance to therapy. Experiments have shown that knocking out GSTM3 significantly reduces tumor cell viability and increases the cells' sensitivity to chemotherapeutic drugs such as cisplatin 63. This finding implies that targeting GSTM3 (inhibiting its function) might be a promising strategy to enhance the efficacy of chemotherapy. Furthermore, certain genetic polymorphisms in GSTM3 (for example, rs1055259) may alter microRNA binding sites, thereby affecting the gene's expression levels 64. Such polymorphisms could lead to differences in individuals' susceptibility to cancer and their responses to treatment, offering a new perspective for precision medicine. Taken together, these insights suggest that GSTM3 could be pursued as a target to increase chemosensitivity, a biomarker for treatment resistance, and a predictor of patient response to chemotherapy or targeted therapy, thereby helping optimize treatment regimens on an individual basis.

Molecular docking is a computer-aided drug screening technique that allows prediction of the binding affinity and binding mode between drug molecules and target proteins. By applying this method, we can screen potential candidate drugs at the molecular level and evaluate their pharmacological properties in silico. As we conducted an in-depth analysis of bladder cancer mechanisms, NOV and GSTM3 emerged as potential druggable targets. Through molecular docking, we examined these targets and identified some promising candidate compounds. Docking results showed that norgestrel (a progestin) has multiple binding sites on NOV, with the top pocket (C1) yielding a Vina score of -7.7 kcal/mol. Meanwhile, GSTM3 displayed high-affinity binding with medroxyprogesterone acetate (MPA) in its primary pocket, suggesting a strong protein-ligand interaction. These compounds might play an important role in bladder cancer therapy. Focusing on NOV, which is known to regulate angiogenesis, our docking study found that norgestrel could occupy the heparin-binding domain of NOV. This implies that norgestrel might inhibit tumor angiogenesis by blocking NOV-mediated VEGF signaling. It is noteworthy that NOV has a bidirectional regulatory function in TGF-β signaling 65. Norgestrel's down-regulation of TGF-β family member expression could form a dynamic balance with NOV's stage-dependent functions during tumor progression - potentially limiting early tumor proliferation while avoiding excessive activation of TGF-β's later pro-metastatic effects 66, 67. Although GSTM3 did not show significant binding with norgestrel in our docking analysis, it did show high-affinity binding with medroxyprogesterone acetate (MPA), suggesting a new therapeutic strategy. GSTM3, being an enzyme related to oxidative stress response, may contribute to tumor cell resistance to chemotherapy through the NF-κB signaling pathway 68. If a progestogenic drug like MPA can bind to and inhibit GSTM3's activity, it could impair the enzyme's ability to clear reactive oxygen species, thereby making tumor cells more susceptible to apoptosis. At the same time, the pro-inflammatory activation of NF-κB by progestogens 69 might complement this effect by enhancing the tumor microenvironment's sensitivity to treatment.

As research on the molecular docking of these targets and drugs progresses, we can further explore their practical applications in bladder cancer therapy, potentially leading to more effective and personalized treatment regimens. Recent studies have identified novel therapeutic targets in bladder cancer, such as the ETS transcription factor ETV4 driving neutrophil-mediated metastasis 70, a pathogenic SLC2A11-MIF fusion protein promoting tumor progression 71, and the ubiquitination enzyme UBE2S facilitating lymphatic spread 72. These discoveries, along with our proteomics-based findings (NOV, GSTM3), highlight the expanding landscape of potential targets for bladder cancer therapy. It should be noted that the drug-target interactions identified in silico still require experimental validation to ensure their feasibility and safety in a clinical context. In the future, with the incorporation of more experimental data (e.g., cell-based assays and clinical studies), we may be able to more definitively confirm the clinical utility of these drug candidates for bladder cancer, paving the way for drug repurposing opportunities and novel targeted therapies.

## 5. Conclusion

In summary, through a proteome-wide large-scale two-sample MR analysis combined with colocalization analysis, we identified SLURP1, LY6D, WFDC1, NOV, and GSTM3 as potential plasma proteins associated with bladder cancer. These proteins may play key roles in tumor progression, immune evasion, and chemotherapy resistance in bladder cancer. Additionally, we found that NOV and GSTM3 can bind with hormone-regulating agents, such as estrogen/progesterone analogues. These discoveries provide new insights into the molecular mechanisms of bladder cancer and offer potential targets for precision therapy and drug repositioning.

## Supplementary Material

Supplementary figure and tables.

## Figures and Tables

**Table 1:** Target proteins identified through MR, SMR, and HEIDI analyses in the Olink dataset

Gene	topSNP	b_SMR	se_SMR	p_SMR	p_HEIDI	FDR
CD59	rs704701	0.247669	0.121393	0.04132839	0.9551517	0.922248634
THBD	rs1042579	-0.323552	0.161533	0.04517623	0.9177106	0.922248634
CTHRC1	rs827592	0.383496	0.178732	0.03190078	0.8221778	0.922248634
HYAL1	rs116482870	0.22468	0.108824	0.03895948	0.8215192	0.922248634
NUDT16	rs150437674	0.238524	0.11553	0.03896035	0.6957886	0.922248634
CRYZL1	rs13050238	0.618195	0.275708	0.02494784	0.6684821	0.90129742
NAGPA	rs12599777	0.255501	0.099485	0.01022189	0.6519896	0.711350111
TNFRSF10C	rs6557616	0.2604	0.11194	0.02000539	0.6218319	0.82813979
FKBP5	rs2817032	-0.377032	0.156637	0.01608206	0.572252	0.769398107
GIPC3	rs34722692	-0.254322	0.120897	0.03541075	0.567346	0.922248634
PNMA1	rs544453779	0.778192	0.354046	0.02794947	0.5556171	0.922248634
TCN2	rs740234	0.131615	0.0585145	0.0244957	0.3741333	0.90129742
DNAJB6	rs12668458	0.307772	0.153609	0.04511251	0.3730661	0.922248634
VSTM2L	rs2273349	-0.567071	0.27269	0.03756722	0.325614	0.922248634
IL1RAP	rs6444442	-0.0589711	0.0295678	0.04610422	0.3132146	0.922248634
CLEC4G	rs76560987	0.353137	0.14834	0.01728486	0.2544816	0.769398107
SUGP1	rs17751061	-0.335589	0.146143	0.02165805	0.2205672	0.878256028
OPLAH	rs55646585	-0.485038	0.158313	0.002185566	0.1537261	0.482524405
PTX3	rs56025932	-1.05649	0.500063	0.034625	0.111485	0.922248634
CXCL8	rs80154360	1.3497	0.468346	0.003953461	0.1000846	0.526270625
CELA2A	rs188524036	1.03831	0.486523	0.03283157	0.09343088	0.922248634
TNFRSF19	rs61947047	0.249152	0.118299	0.03519323	0.07236322	0.922248634
NINJ1	rs7033638	-0.314766	0.12704	0.01322328	0.06405632	0.766160155

**Table 2:** Target proteins identified through MR, SMR, and HEIDI analyses in the Decode dataset

Gene	topSNP	b_SMR	se_SMR	p_SMR	p_HEIDI	FDR
HEXB	rs13164140	0.300085	0.117384	0.01057492	0.0500748	0.78737568
IGDCC4	rs35223184	-0.209638	0.0985616	0.03342238	0.09033423	0.867927119
PLAT	rs2020921	-0.571203	0.271343	0.03528278	0.09968545	0.867927119
**GSTM3**	**rs4970772**	**-0.18263**	**0.0503645**	**0.00028766**	**0.1031404**	**0.234417238**
**NOV**	**rs11779998**	**1.83052**	**0.517887**	**0.00040839**	**0.2116264**	**0.234417238**
GPD1	rs11619196	-1.61201	0.784549	0.03990797	0.3131	0.867927119
MFGE8	rs34239095	0.3661	0.129172	0.00459412	0.3149904	0.78737568
BPHL	rs9503407	0.656715	0.258754	0.01114905	0.4747127	0.78737568
PTPRU	rs2179795	0.465239	0.222898	0.03686792	0.5691693	0.867927119
JAG1	rs2423507	0.941225	0.45212	0.03736022	0.5942881	0.867927119
VASN	rs757593	-1.28247	0.419623	0.00224119	0.8061312	0.551333724
HYAL1	rs11648287	0.514518	0.252096	0.04125478	0.8839827	0.867927119
PSMB1	rs756519	0.192116	0.0936547	0.04023559	0.8986812	0.867927119
RHOC	rs10745330	-0.924833	0.297119	0.00185401	0.9614418	0.532102592
CD59	rs831630	0.336234	0.163498	0.03973439	0.9757196	0.867927119

**Table 3:** NOV_related_drugs_DSigDB.

Gene	Source	Chemical Name
NOV	D4 CTD	LUCANTHONE
NOV	D4 CTD	N-NITROSODIETHYLAMINE
NOV	D4 CTD	Fulvestrant
NOV	D4 CTD	TERT-BUTYL HYDROPEROXIDE
NOV	D4 CTD	tamoxifen
NOV	D4 CTD	ARSENIC
NOV	D4 CTD	5-Fluorouracil
NOV	D4 CTD	4-Hydroxytamoxifen
NOV	D4 CTD	cycloheximide
NOV	D4 CTD	hydrogen peroxide
NOV	D4 CTD	raloxifene
NOV	D4 CTD	estradiol
NOV	D4 CTD	Andriol
NOV	D4 CTD	trichostatin A
NOV	D4 CTD	norgestrel
NOV	D4 CTD	Arsenenous acid
NOV	D4 CTD	VALPROIC ACID
NOV	D4 CTD	8-Bromo-cAMP, Na
NOV	D4 CTD	progesterone

**Table 4:** GSTM3_related_drugs_DSigDB.

Gene	Source	Chemical Name
GSTM3	D4 CTD	D-Penicillamine
GSTM3	D4 CTD	PARAQUAT
GSTM3	D4 CTD	emodin
GSTM3	D4 CTD	diethylstilbestrol
GSTM3	D4 CTD	FCCP
GSTM3	D4 CTD	carmustine
GSTM3	D4 CTD	2,6-DICHLOROINDOPHENOL
GSTM3	D4 CTD	tetracycline
GSTM3	D4 CTD	DL-Homocysteine
GSTM3	D4 CTD	1-chloro-2,4-dinitrobenzene
GSTM3	D4 CTD	Decitabine
GSTM3	D4 CTD	troglitazone
GSTM3	D4 CTD	ARSENIC
GSTM3	D4 CTD	Cylindrospermopsin
GSTM3	D4 CTD	CUMENE HYDROPEROXIDE
GSTM3	D4 CTD	ZERANOL
GSTM3	D4 CTD	Tetradioxin
GSTM3	D4 CTD	CADMIUM
GSTM3	D4 CTD	estradiol
GSTM3	D4 CTD	NICKEL
GSTM3	D4 CTD	PHENCYCLIDINE
GSTM3	D4 CTD	diazinon
GSTM3	D4 CTD	Retinoic acid
GSTM3	D4 CTD	glutathione
GSTM3	D4 CTD	Disodium selenite
GSTM3	D4 CTD	42935-17-1
GSTM3	D4 CTD	benzo[a]pyrene
GSTM3	D4 CTD	Nonenal
GSTM3	D4 CTD	trichostatin A
GSTM3	D4 CTD	Arsenenous acid
GSTM3	D4 CTD	VALPROIC ACID
GSTM3	D4 CTD	Dinoprostone
GSTM3	D4 CTD	Medroxyprogesterone acetate
GSTM3	D4 CTD	L-methionine

**Table 5:** Docking results of NOV and Norethisterone.

CurPocket ID	Vina score	Cavity volume (Å)	Center (x, y, z)	Docking size (x, y, z)
C1	-7.7	1071	8, 5, 6	21, 21, 21
C3	-7.2	317	8, 11, -4	21, 21, 21
C2	-6.7	396	-14, 21, 21	21, 21, 21
C5	-6.4	201	-16, 30, 29	21, 21, 21
C4	-6.2	219	3, 19, 13	21, 21, 21

**Table 6:** Docking results of GSTM3 and Medroxyprogesterone Acetate.

CurPocket ID	Vina score	Cavity volume (Å)	Center (x, y, z)	Docking size (x, y, z)
C1	-8.5	2456	-10, -22, 83	31, 21, 21
C3	-7.3	702	-24, -28, 54	27, 21, 21
C4	-7.1	643	-1, -15, 106	21, 21, 21
C2	-6.3	853	-8, -27, 60	21, 21, 21
C5	-6.3	531	-17, -13, 103	21, 21, 21
