# Supplementary material for: Comprehensive Mendelian randomization and colocalization analysis of plasma proteomics to identify new therapeutic targets for bladder cancer
Source: J Cancer. 2025 Jul 11;16(10):3163–79. doi: 10.7150/jca.116402 (PMC12305583; doi:10.7150/jca.116402)

Supplementary Table 1.R11_BLADDER_CANCER.olink_MR.sig

protein	b	se	method	pval	adjp
ADAMTS15	-0.171584708	0.0540812391042546	IVW	0.001510173	0.02991581 4
ADGRE1	0.118420216694284	0.0357468539177951	IVW	0.000923851	0.01960827 1
ANXA2	-0.13509392	0.0295402748766186	IVW	4.80E-06	0.00017224 7
AOC3	-0.184210798	0.0474746340654504	IVW	0.000104373	0.00297391 9
ARG2	1.19773431544021	0.0183342469323023	IVW	0	0
BCAT1	-0.052242772	0.00954712279441304	IVW	4.45E-08	2.01E-06
BCL2L15	-0.161228599	0.0166465789332504	IVW	3.48E-22	3.15E-20
BOLA2	0.632500298984342	0.170953348123554	IVW	0.000215734	0.00560908 7
CA9	0.177562738647464	0.0512578196099805	IVW	0.000531988	0.01257427

					2
CC2D1A	-0.910276664	0.234914375333681	IVW	0.000106656	0.002997885
CD163	-0.354128393	0.106429151534828	IVW	0.000876722	0.019268738
CD27	0.209095477647414	0.0511436822762082	IVW	4.34E-05	0.001368929
CD40	0.12334018379817	0.0395480357765482	IVW	0.001816239	0.034979424
CD59	0.232333632073691	0.036108414010474	IVW	1.24E-10	6.97E-09
CDH23	0.161609171594586	0.0197385924028499	IVW	2.67E-16	1.85E-14
CEACAM19	-0.14749201	0.0293693905648211	IVW	5.11E-07	2.01E-05
CELA2A	0.805865378222348	0.253703594068721	IVW	0.001491126	0.029822518
CHCHD10	0.067181408631974	0.0104861274175567	IVW	1.49E-10	7.93E-09
CLEC4G	0.329430214467772	0.0846240606472164	IVW	9.91E-05	0.00286192
COL15A1	0.276573446775184	0.0318652587137678	IVW	3.98E-18	2.95E-16

CRYZL1	0.657189077449012	0.219034808179539	IVW	0.002696372	0.04876917
CSF1R	0.211990838035037	0.0607051431297431	IVW	0.000479168	0.011455962
CST6	0.273601480698931	0.0254373763672102	IVW	5.56E-27	5.51E-25
CTHRC1	0.751208603277708	0.214990560346621	IVW	0.000475579	0.011455962
DNAJB6	0.311834576321783	0.0175156003971254	IVW	6.67E-71	9.91E-69
ECI2	0.18058925267285	0.0291465731635771	IVW	5.80E-10	3.01E-08
ENTR1	-0.687123268	0.0757170017327822	IVW	1.14E-19	8.77E-18
EPHA1	0.16600737928858	0.0181007660469745	IVW	4.67E-20	3.89E-18
FABP1	-0.076398237	0.000582413980214646	IVW	0	0
FABP5	-0.182184021	0.031626004508838	IVW	8.38E-09	4.05E-07
FCRL1	-0.115584254	0.0201014967872333	IVW	8.92E-09	4.12E-07
FGFBP3	-0.202143863	0.053464317377939	IVW	0.000156255	0.004276455
FKBP5	-0.368999355	0.0426248871600964	IVW	4.85E-18	3.48E-16
FKBP7	-0.308782943	0.0427997488630826	IVW	5.41E-13	3.41E-11

FOXO1	0.222838324197552	0.0128010142867021	IVW	7.19E-68	9.97E-66
GAL	-0.301353469	0.0312349320697566	IVW	5.01E-22	4.35E-20
GBP1	-0.141201671	0.00256254464608124	IVW	0	0
GFRA1	0.0767852214382158	0.0241001176742651	IVW	0.001442081	0.029121641
GIMAP8	-0.284915763	0.0933661097820722	IVW	0.002276273	0.042273643
GIPC3	-0.248617231	0.0331467593315186	IVW	6.36E-14	4.13E-12
GLT8D2	-0.059826335	0.00653211110921973	IVW	5.25E-20	4.20E-18
GUSB	-0.269448202	0.0739780170766261	IVW	0.00027024	0.006939504
HCG22	-0.096115803	0.00158284328631523	IVW	0	0
HPCAL1	-0.116702321	0.00188321220080559	IVW	0	0
HYAL1	0.230891325625108	0.0619110343137974	IVW	0.000191932	0.005184667
IDO1	-0.16744347	0.0450041194931802	IVW	0.00019873	0.005232386

IDO1	-0.153679781	0.0462419674354341	IVW	0.000889326	0.019268738
IFNAR1	0.0971700986213467	0.0194650782213147	IVW	5.98E-07	2.30E-05
IL1RAP	-0.084063826	0.025101379137173	IVW	0.000811119	0.018141151
INSL5	-0.168544925	0.0558416238495648	IVW	0.002542249	0.046384892
ITGB6	0.177968093116159	0.0526046401123347	IVW	0.000716659	0.016380779
JAM2	-0.060813818	0.013561363543421	IVW	7.31E-06	0.000253547
KIT	0.228988426852979	0.0100899034693573	IVW	5.04E-114	8.07E-112
LAG3	0.288926314990548	0.0256766437281957	IVW	2.25E-29	2.46E-27
LRTM2	0.069825844848468	0.0103343458546557	IVW	1.41E-11	8.39E-10
LTBP2	0.375001251550079	0.0147015093731555	IVW	1.62E-143	2.81E-141
LY6D	0.448546378909126	0.125506402261898	IVW	0.000351713	0.00870909

					6
MASP1	-0.124583915	0.0318378246933393	IVW	9.11E-05	0.00266963
MTSS2	0.586446913245916	0.139819193881882	IVW	2.74E-05	0.000889484
NAGPA	0.27148428266362	0.0769821327566306	IVW	0.000420951	0.010300916
NBL1	-0.22363695	0.0280537234288284	IVW	1.56E-15	1.05E-13
NBN	0.314172236986114	0.0765608576741392	IVW	4.07E-05	0.001301879
NID1	0.042256963693485	0.00813396049471499	IVW	2.05E-07	8.18E-06
NINJ1	-0.304449683	0.0613694337456264	IVW	7.02E-07	2.65E-05
NPDC1	0.136771108453491	0.0254596429832737	IVW	7.78E-08	3.30E-06
NRP2	0.264482981825651	0.0485471271988904	IVW	5.09E-08	2.25E-06
NTRK2	0.154789502811216	0.0465634060321955	IVW	0.000886492	0.019268738
NUDT16	0.253856069022054	0.0544878713487283	IVW	3.18E-06	0.000115981

OGA	0.0463214576096985	0.0115438480647778	IVW	6.00E-05	0.001809905
OPLAH	-0.485037789	0.157060755775137	Wald ratio	0.00201361	0.038424847
PAPPA	-0.269003709	0.0797847583289848	IVW	0.000747281	0.016895044
PBLD	0.163250729414275	0.049963076033106	IVW	0.001085297	0.022730494
PDE5A	-0.315777238	0.0117443478628073	IVW	3.07E-159	5.80E-157
PDIA5	0.0077510669341651	0.000473842203416335	IVW	3.82E-60	4.97E-58
PLEKHO1	0.891045916585434	0.0266090686368847	IVW	7.57E-246	1.57E-243
PNMA1	0.770496658702945	0.018249921075693	IVW	0	0
PROC	0.210267250185702	0.0159045895650278	IVW	6.68E-40	7.72E-38
PROK1	-0.052163313	0.015311867839317	IVW	0.000657475	0.015194976
PRSS2	-0.176828014	0.00465208400236552	IVW	0	0

PRSS8	-0.04148176	0.0127031377116187	IVW	0.001092812	0.022730494
PSAPL1	0.249519702628767	0.0573198663519533	IVW	1.34E-05	0.000443138
PSRC1	-0.251978059	0.043807527073465	IVW	8.82E-09	4.12E-07
PTPRM	0.633013271335674	0.139284958539847	IVW	5.50E-06	0.000193914
PTS	0.114911780027202	0.0105363936611895	IVW	1.08E-27	1.12E-25
PTX3	-0.936145858	0.176850116268913	IVW	1.20E-07	4.99E-06
REG4	-0.160228285	0.0466170056930067	IVW	0.000587933	0.013740458
RELT	0.201393242763715	0.045837708596106	IVW	1.11E-05	0.000374001
RIDA	0.0315140961909655	0.00200135364714929	IVW	7.27E-56	8.90E-54
RNASE1	-0.147563957	0.0409375391545186	IVW	0.000312619	0.007834314

SBSN	0.0940404415432466	0.0299831546062214	IVW	0.001710109	0.033525794
SERPINA6	0.155855731488201	0.0386754931131582	IVW	5.58E-05	0.001707337
SERPINF2	-0.170281855	0.0314507012971251	IVW	6.15E-08	2.67E-06
SERPING1	0.0685606905498257	0.0130357866645596	IVW	1.45E-07	5.89E-06
SFTPA1	0.12196593579336	0.0120173741608576	IVW	3.34E-24	3.16E-22
SLA2	-0.502567696	0.156426019734394	IVW	0.001314414	0.027069123
SLURP1	0.442751776602581	0.100260741237061	IVW	1.01E-05	0.000342833
SMPD1	-1.186409951	0.357995523814684	IVW	0.000919598	0.019608271
SNX9	-0.166472874	0.045750472068251	IVW	0.000274003	0.006950323
STX16	-0.748701317	0.00153443292534928	IVW	0	0

SUGP1	-0.331484718	0.0460824301448701	IVW	6.32E-13	3.87E-11
SUMF2	0.152856055880909	0.0495789532713778	IVW	0.002048676	0.038738604
SUOX	-1.383677162	0.215530458782675	IVW	1.36E-10	7.47E-09
SUSD1	-0.129851451	0.0275309928983858	IVW	2.40E-06	8.91E-05
TBCC	0.117563287865727	0.0384574759573455	IVW	0.002235879	0.041897545
TCN2	0.116868362708453	0.0385346009844612	IVW	0.002422831	0.044597245
THBD	-0.278644245	0.0748658793144074	IVW	0.00019772	0.005232386
THTPA	-0.034269448	0.00531587987242073	IVW	1.14E-10	6.61E-09
TNFRSF10C	0.217698812709505	0.0679149914511734	IVW	0.001348466	0.027498134
TNFRSF12A	0.504043423034292	0.0817340846634728	IVW	6.97E-10	3.53E-08
TNFRSF19	0.265789847786271	0.0453050998480762	IVW	4.45E-09	2.20E-07
TRIM5	0.049870297082587	0.0124538730309173	IVW	6.22E-05	0.00184748

	1				3
VCPKMT	-0.756675723	0.185419046731498	IVW	4.49E-05	0.001392748
VSNL1	-0.034927284	0.0111447770759388	IVW	0.001724644	0.033525794
VSTM2L	-0.463648678	0.121502478292982	IVW	0.000135654	0.003762141
WFDC1	0.327995857147173	0.00883067182277297	IVW	5.73E-302	1.32E-299

Supplementary Table 2.R11_BLADDER_CANCER.decode_MR.sig

protein_ID	b	se	method	pval	adjp
PDGFRA	-0.362603925	0.048387042	IVW	6.69E-14	5.36E-12
NUCB1	-0.120965075	0.003037484	IVW	0	0
TMEM106A	-0.137810687	0.009602234	IVW	1.04E-46	1.99E-44
PLOD3	0.361444616	0.03114239	IVW	3.83E-31	5.67E-29
STX4	1.062243764	0.077511699	IVW	9.57E-43	1.67E-40

KLRB1	-0.167924174	0.014884404	IVW	1.61E-29	1.94E-27
CD59	0.296776811	0.064639486	IVW	4.41E-06	0.000162843
S100A2	0.079168863	0.022231312	IVW	0.000369245	0.009721769
CRYGD	-0.599101258	0.159424229	IVW	0.000171341	0.005145592
MPG	0.618335419	0.103354325	IVW	2.20E-09	1.21E-07
SAT2	-0.235118041	0.071117836	IVW	0.000946263	0.022566259
PSMB1	0.205279256	0.043962148	IVW	3.02E-06	0.000118458
CRAT	0.12807381	0.007735784	IVW	1.45E-61	3.98E-59
ACLY	-0.912347325	0.104786664	IVW	3.13E-18	2.86E-16
EIF1AD	0.279865596	0.012940813	IVW	1.01E-103	4.85E-101
UAP1	-0.279791988	0.059625163	IVW	2.70E-06	0.000110358

GPD1	0.43930043	0.071461788	IVW	7.88E-10	4.92E-08
BDNF	-0.244243487	0.02152385	IVW	7.62E-30	9.77E-28
EFNB2	0.36848225	0.055830214	IVW	4.11E-11	2.93E-09
RAB6B	0.048088007	0.009585199	IVW	5.25E-07	2.35E-05
CST6	0.331479678	0.065893665	IVW	4.89E-07	2.24E-05
APOC1	-0.451560703	0.01921172	IVW	3.67E-122	2.35E-119
BRD2	-1.996924025	0.592634497	Wald ratio	0.000752853	0.018551067
LOXL3	0.33298192	0.100762283	IVW	0.000951023	0.022566259
CD8A	0.157453595	0.043044869	IVW	0.000254296	0.007106812
HEXB	0.298960137	0.008780099	IVW	4.09E-254	3.93E-251
EPHB4	-0.451469274	0.046663197	IVW	3.85E-22	3.89E-20
PNLIP	-0.056212703	0.016115049	IVW	0.000486263	0.012137624
RBP4	0.085115316	0.023813692	IVW	0.00035127	0.00937714

				7	
CD14	-0.127393252	0.024363541	IVW	1.71E-07	8.20E-06
ADPRHL2	-0.114190831	0.01844642	IVW	6.00E-10	3.98E-08
HMOX1	1.456299079	0.239080509	IVW	1.12E-09	6.73E-08
RHOC	-0.924832665	0.287293587	Wald ratio	0.001285845	0.029775822
BPHL	0.612055742	0.074454045	IVW	2.03E-16	1.77E-14
PSAT1	-0.183252813	0.011770898	IVW	1.20E-54	2.87E-52
GMPR	0.135896753	0.03674176	IVW	0.000216706	0.006310753
UROD	0.172087688	0.043767258	IVW	8.43E-05	0.002745641
PLAT	-0.458854939	0.092369716	IVW	6.78E-07	2.96E-05
TIMP2	0.187128003	0.052978191	IVW	0.000412164	0.010562377
VEGFA	-0.155964271	0.046451272	IVW	0.000786255	0.019128881

NOV	1.830522293	0.469103185	Wald ratio	9.53E-05	0.003053819
CD36	0.260748292	0.043029634	IVW	1.36E-09	7.71E-08
IL1RL2	0.281933073	0.024308169	IVW	4.20E-31	5.77E-29
CCL3	0.075389969	0.01136205	IVW	3.24E-11	2.39E-09
PIGR	-0.103655101	0.028627141	IVW	0.000293616	0.007948315
ACY1	0.042887908	0.009306905	IVW	4.06E-06	0.000156145
TNFSF8	-0.044769184	0.004163071	IVW	5.68E-27	6.42E-25
EPHA5	0.349643661	0.098347148	IVW	0.000377693	0.009809821
LDHB	0.263588679	0.064980592	IVW	4.98E-05	0.001651272
NTF3	-0.412298186	0.080583729	IVW	3.11E-07	1.46E-05
CRP	0.096642248	0.007303103	IVW	5.66E-40	9.06E-38
MFGE8	0.248247013	0.066480798	IVW	0.00018837	0.00557012

				6	3
EPB41	-0.225666902	0.037143976	IVW	1.24E-09	7.20E-08
SMPDL3A	0.117649097	0.00657557	IVW	1.37E-71	4.37E-69
SHBG	0.246857518	0.02507142	IVW	7.12E-23	7.60E-21
CD163	-0.469580596	0.095154207	IVW	8.02E-07	3.42E-05
JAG1	0.855404751	0.178031755	IVW	1.55E-06	6.47E-05
KIR2DL4	-0.166121976	0.052465946	IVW	0.001544011	0.035328436
KIR3DS1	0.725997468	0.126673465	IVW	9.97E-09	5.32E-07
FOLH1	-0.333363876	0.037554768	IVW	6.88E-19	6.62E-17
RGMA	-0.249518258	0.040597503	IVW	7.94E-10	4.92E-08
POFUT1	-0.215338172	0.058883037	IVW	0.000255135	0.007106812
VASN	-1.282474619	0.392181472	Wald ratio	0.001075075	0.02519872
GZMM	0.364063433	0.08520788	IVW	1.93E-05	0.000662965

PSAP	0.036993018	0.008324821	IVW	8.84E-06	0.000314725
SEMA7A	0.083355019	0.020403373	IVW	4.40E-05	0.00148397
ST6GALNAC6	-0.817883508	0.224233267	IVW	0.000264838	0.007271695
TPST2	0.227912434	0.041827217	IVW	5.07E-08	2.63E-06
HYAL1	0.525153077	0.036158606	IVW	8.59E-48	1.83E-45
PTPRU	0.401962891	0.088138762	IVW	5.10E-06	0.000185002
QSOX2	0.201701827	0.05473409	IVW	0.000228593	0.006557538
CD14	-0.166264347	0.043807383	IVW	0.000147435	0.004570497
MAN1A2	0.286282642	0.015709392	IVW	3.35E-74	1.29E-71
POMC	-0.438158064	0.115634364	IVW	0.000151153	0.004611351
TACO1	1.880739329	0.354348776	IVW	1.11E-07	5.47E-06

PTK7	-0.180101359	0.027761675	IVW	8.73E-11	5.99E-09
MANSC1	0.124055183	0.035201357	IVW	0.000424829	0.010743705
B4GALT2	0.104099867	0.013867576	IVW	6.06E-14	5.07E-12
GSTM3	-0.170433901	0.039571225	IVW	1.65E-05	0.000578248
IGDCC4	-0.174678641	0.045054446	IVW	0.000105728	0.003331291
FBP2	-0.233762529	0.049908866	IVW	2.82E-06	0.000112766
KRT1	0.333138875	0.072439959	IVW	4.25E-06	0.00016012
LAG3	0.348829058	0.065144051	IVW	8.57E-08	4.33E-06
KIAA1467	-0.148748324	0.020068762	IVW	1.24E-13	9.57E-12

Supplement table3.R11_BLADDER_CANCER.olink.coloc.results

protein	SNP	PP.H0	PP.H1	PP.H2	PP.H3	PP.H4	p1	p2	PP.H3+PP.H4
SLURP1	rs150372266	1.04E-306	9.22E-05	1.06E-303	0.093468148	0.906439697	3.16E-308	5.80E-09	0.999907845
SLURP1	rs150571771	1.03E-306	0.000101967	1.05E-303	0.103769438	0.896128596	3.16E-308	5.80E-09	0.999898034
LY6D	rs189511655	1.01E-306	0.000123364	1.04E-303	0.125761158	0.874115479	3.16E-308	5.80E-09	0.999876637
SLURP1	rs587754933	9.69E-307	0.000149648	9.95E-304	0.152793428	0.847056924	3.16E-308	5.80E-09	0.999850352
SLURP1	rs78999750	9.69E-307	0.000149648	9.95E-304	0.152793321	0.847057031	3.16E-308	5.80E-09	0.999850352
SLURP1	rs9297976	9.69E-307	0.000149648	9.95E-304	0.152793302	0.84705705	3.16E-308	5.80E-09	0.999850352
SLURP1	rs146070037	9.69E-307	0.000149648	9.95E-304	0.152792742	0.847057611	3.16E-308	5.80E-09	0.999850353
SLURP1	rs186061604	9.69E-307	0.000149648	9.95E-304	0.152791879	0.847058473	3.16E-308	5.80E-09	0.999850352
SLURP1	rs139509793	9.69E-307	0.000149648	9.95E-304	0.152791877	0.847058475	3.16E-308	5.80E-09	0.999850352
LY6D	rs78999750	9.76E-307	0.000151344	1.00E-303	0.154536728	0.845311928	3.16E-308	5.80E-09	0.999848656
LY6D	rs2976384	9.76E-307	0.000151344	1.00E-303	0.154536707	0.845311949	3.16E-308	5.80E-09	0.999848656
LY6D	rs72690905	9.76E-307	0.000151344	1.00E-303	0.154536561	0.845312095	3.16E-308	5.80E-09	0.999848656
SLURP1	rs28418310	1.10E-306	0.000162698	1.00E-303	0.147838977	0.851998325	3.16E-308	5.80E-09	0.999837302
LY6D	rs28418310	1.09E-306	0.000164825	9.99E-304	0.149784389	0.850050786	3.16E-308	5.80E-09	0.999835175

**WFDC1**	**rs183277843**	**2.56E-274**	**0.241675815**	**3.25E-274**	**0.306062972**	**0.452261213**	**2.18E-281**	**0.000290376**	**0.758324185**
**WFDC1**	**rs400345**	**2.56E-274**	**0.241733121**	**3.25E-274**	**0.305898428**	**0.452368451**	**2.18E-281**	**0.000290376**	**0.758266879**
**WFDC1**	**rs244783**	**2.56E-274**	**0.241765454**	**3.25E-274**	**0.305805589**	**0.452428957**	**2.18E-281**	**0.000290376**	**0.758234546**
NME3	rs12599644	1.13E-144	0.354309662	9.71E-146	0.029943176	0.615747162	1.25E-168	0.00239359	0.645690338
NME3	rs572568515	1.13E-144	0.355198563	8.94E-146	0.027509475	0.617291963	1.25E-168	0.00239359	0.644801438
NME3	rs140703991	1.13E-144	0.355211307	8.93E-146	0.027474582	0.617314111	1.25E-168	0.00239359	0.644788693
NME3	rs552834368	1.13E-144	0.355213488	8.93E-146	0.027468611	0.617317901	1.25E-168	0.00239359	0.644786512
NME3	rs192671531	1.13E-144	0.355275799	8.87E-146	0.02729801	0.617426191	1.25E-168	0.00239359	0.644724201
NME3	rs533463510	1.13E-144	0.355397797	8.77E-146	0.026963994	0.617638208	1.25E-168	0.00239359	0.644602202
NME3	rs529829827	4.07E-124	0.393504781	3.22E-125	0.030579627	0.575915592	1.04E-147	0.00302991	0.606495219
NME3	rs561234506	4.07E-124	0.393729704	3.16E-125	0.030025518	0.576244778	1.04E-147	0.00302991	0.606270296
NME3	rs182725865	4.07E-124	0.393811279	3.14E-125	0.029824552	0.576364169	1.04E-147	0.00302991	0.606188721
NME3	rs187239742	4.07E-124	0.394023551	3.09E-125	0.029301609	0.57667484	1.04E-147	0.00302991	0.605976449
CCN3	rs2279112	1.21E-246	0.510549873	9.42E-247	0.398136862	0.091313265	1.91E-253	0.00436385	0.489450127
CCN3	rs139806626	1.21E-246	0.510596969	9.41E-247	0.398081343	0.091321688	1.91E-253	0.00436385	0.489403031
CCN3	rs117560584	1.22E-246	0.514191884	9.31E-247	0.393843468	0.091964648	1.91E-253	0.00436385	0.485808116
CCN3	rs545022235	1.22E-246	0.514669781	9.30E-247	0.393280098	0.092050122	1.91E-253	0.00436385	0.48533022

CCN3	rs534145431	1.22E-246	0.516109275	9.26E-247	0.391583145	0.092307579	1.91E-253	0.00436385	0.483890724
CCN3	rs13279941	1.23E-246	0.52065635	9.13E-247	0.386222813	0.093120836	1.91E-253	0.00436385	0.479343649
CCN3	rs140495261	2.06E-246	0.557525824	1.28E-246	0.345772561	0.096701615	1.91E-253	0.00436385	0.442474176
CCN3	rs543197801	1.32E-246	0.558988482	8.07E-247	0.341032712	0.099978806	1.91E-253	0.00436385	0.441011518
PKD1	rs180831566	2.91E-206	0.585394228	1.74E-207	0.034652629	0.379953143	1.24E-220	0.00788806	0.414605772
PKD1	rs148501094	2.92E-206	0.586010397	1.69E-207	0.033636532	0.380353071	1.24E-220	0.00788806	0.413989603
PKD1	rs575316301	2.92E-206	0.586037625	1.69E-207	0.033591632	0.380370743	1.24E-220	0.00788806	0.413962375
PKD1	rs199476099	2.92E-206	0.587417354	1.58E-207	0.031316383	0.381266263	1.24E-220	0.00788806	0.412582646
PKD1	rs141274774	2.92E-206	0.587492128	1.57E-207	0.031193077	0.381314795	1.24E-220	0.00788806	0.412507872
PKD1	rs150545244	2.93E-206	0.588297282	1.51E-207	0.029865334	0.381837385	1.24E-220	0.00788806	0.411702719
TCN2	rs117825799	4.83E-153	0.643218559	2.55E-153	0.33889753	0.017883912	4.73E-160	0.0339508	0.356781442
SLURP1	rs4073672	1.26E-302	0.70513513	3.36E-303	0.187496019	0.107368851	3.16E-308	0.000710526	0.29486487
IL1RAP	rs138134491	9.99E-302	0.716117004	3.80E-302	0.272385683	0.011497313	3.16E-308	0.0122882	0.283882996
IL1RAP	rs62286312	6.92E-300	0.71806031	2.60E-300	0.269933335	0.012006355	3.16E-308	0.0768706	0.28193969
PCDH9	rs571044032	5.68E-56	0.725460995	2.52E-57	0.031945819	0.242593186	1.65E-65	0.0116359	0.274539005
PCDH9	rs138637737	5.68E-56	0.725537927	2.51E-57	0.031843161	0.242618912	1.65E-65	0.0116359	0.274462073
GPHA2	rs111479117	3.58E-167	0.730153225	2.20E-168	0.044705403	0.225141372	4.02E-174	0.0088369	0.269846775

GPHA2	rs112310647	3.58E-167	0.730237979	2.20E-168	0.044594516	0.225167506	4.02E-174	0.0088369	0.269762022
GPHA2	rs61736624	3.58E-167	0.730490409	2.18E-168	0.044264249	0.225245342	4.02E-174	0.0088369	0.269509591
GPHA2	rs114391421	3.59E-167	0.732977094	2.02E-168	0.0410108	0.226012106	4.02E-174	0.0088369	0.267022906
GPHA2	rs142007717	3.59E-167	0.733003195	2.02E-168	0.04097665	0.226020155	4.02E-174	0.0088369	0.266996805
IL1RAP	rs112634485	1.25E-299	0.733012127	4.41E-300	0.257909062	0.009078811	3.16E-308	0.129966	0.266987873
GPHA2	rs528923036	3.60E-167	0.735145077	1.88E-168	0.038174323	0.2266806	4.02E-174	0.0088369	0.264854923
MICB	rs183924128	6.67E-304	0.735561105	2.22E-304	0.244590743	0.019848153	3.16E-308	0.000494265	0.264438896
MICB	rs182579367	6.68E-304	0.736264074	2.21E-304	0.243888746	0.019847181	3.16E-308	0.000494265	0.263735927
MICB	rs146335452	6.68E-304	0.737995029	2.19E-304	0.24214126	0.019863711	3.16E-308	0.000494265	0.262004971
MICB	rs570197742	6.66E-304	0.738983868	2.17E-304	0.241148543	0.019867589	3.16E-308	0.000494265	0.261016132
MICB	rs71563313	6.67E-304	0.73898721	2.18E-304	0.241143612	0.019869178	3.16E-308	0.000494265	0.26101279
MICB	rs2395474	6.67E-304	0.739063927	2.17E-304	0.241062291	0.019873782	3.16E-308	0.000494265	0.260936073
MICB	rs189334061	6.67E-304	0.739918266	2.17E-304	0.240173178	0.019908555	3.16E-308	0.000494265	0.260081733
MICB	rs567272618	6.69E-304	0.740810245	2.16E-304	0.239241199	0.019948556	3.16E-308	0.000494265	0.259189755
MICB	rs149197376	6.88E-304	0.743215753	2.19E-304	0.236354713	0.020429534	3.16E-308	0.000494265	0.256784247
MICB	rs185443623	6.78E-304	0.743555612	2.16E-304	0.236217616	0.020226772	3.16E-308	0.000494265	0.256444388
MICB	rs11962863	6.80E-304	0.743690568	2.16E-304	0.236036539	0.020272893	3.16E-308	0.000494265	0.256309432

LY6D	rs76595729	1.34E-302	0.745157369	3.43E-303	0.189935817	0.064906814	3.16E-308	0.00076498	0.254842631
MICB	rs72863211	6.49E-304	0.745355535	2.05E-304	0.235194125	0.01945034	3.16E-308	0.000494265	0.254644465
MICB	rs185701317	7.34E-304	0.749083497	2.25E-304	0.229599065	0.021317438	3.16E-308	0.000494265	0.250916503
CDON	rs7104745	8.81E-41	0.750004921	4.62E-42	0.039068033	0.210927046	4.80E-47	0.00350018	0.249995079
MICB	rs148181670	7.35E-304	0.751128739	2.23E-304	0.227575173	0.021296088	3.16E-308	0.000494265	0.248871261
MICB	rs114834343	6.66E-304	0.751525264	2.03E-304	0.228867208	0.019607528	3.16E-308	0.000494265	0.248474736
MICB	rs41545712	6.66E-304	0.752445655	2.02E-304	0.227928348	0.019625998	3.16E-308	0.000494265	0.247554346
CDON	rs607980	8.85E-41	0.75276007	4.20E-42	0.035538042	0.211701888	4.80E-47	0.00350018	0.24723993
MICB	rs540165558	7.26E-304	0.755142302	2.15E-304	0.223779962	0.021077737	3.16E-308	0.000494265	0.244857699
MICB	rs534557603	6.86E-304	0.756788977	2.02E-304	0.223407354	0.019803669	3.16E-308	0.000494265	0.243211023
MICB	rs551312866	7.02E-304	0.758449602	2.05E-304	0.221432458	0.02011794	3.16E-308	0.000494265	0.241550398
MICB	rs200665392	7.94E-304	0.761230032	2.26E-304	0.216246247	0.022523721	3.16E-308	0.000494265	0.238769968
ORM1	rs142414765	2.75E-300	0.762026484	8.32E-301	0.230266649	0.007706867	3.16E-308	0.356768	0.237973516
ORM1	rs116994374	2.75E-300	0.76211283	8.32E-301	0.230179429	0.00770774	3.16E-308	0.356768	0.237887169
ORM1	rs145653978	2.75E-300	0.762334121	8.31E-301	0.229955901	0.007709978	3.16E-308	0.356768	0.237665879
MICB	rs181086161	8.56E-304	0.76254643	2.40E-304	0.213664606	0.023788963	3.16E-308	0.000494265	0.237453569
MICB	rs545056843	8.71E-304	0.765346146	2.40E-304	0.210509604	0.02414425	3.16E-308	0.000494265	0.234653854

CELA2A	rs115985482	2.40E-32	0.766791269	1.27E-33	0.040475122	0.192733609	8.98E-40	0.0257419	0.233208731
MICB	rs147355206	7.84E-304	0.773631462	2.08E-304	0.204969689	0.021398848	3.16E-308	0.000494265	0.226368537
MICB	rs9267158	7.91E-304	0.776207745	2.07E-304	0.202836582	0.020955673	3.16E-308	0.000494265	0.223792255
LY6D	rs74697140	2.03E-302	0.778431701	4.86E-303	0.18665631	0.03491199	3.16E-308	0.00117136	0.2215683
MICB	rs9689414	8.24E-304	0.779985136	2.10E-304	0.199056001	0.020958863	3.16E-308	0.000494265	0.220014864
CELA2A	rs190159165	2.57E-44	0.787307198	1.76E-45	0.053811286	0.158881516	6.26E-52	0.031115	0.212692802
CELA2A	rs188524036	2.57E-44	0.78832749	1.72E-45	0.052585096	0.159087414	6.26E-52	0.031115	0.21167251
CELA2A	rs11579489	2.57E-44	0.788344485	1.72E-45	0.052564671	0.159090844	6.26E-52	0.031115	0.211655515
MICB	rs565938259	1.15E-303	0.790469024	2.79E-304	0.192102155	0.017428822	3.16E-308	0.000494265	0.209530977
CTSS	rs587692431	2.11E-302	0.795189068	4.00E-303	0.151092866	0.053718066	3.16E-308	0.00507399	0.204810932
CTSS	rs150225445	8.45E-302	0.797415793	1.49E-302	0.140269206	0.062315001	3.16E-308	0.00507399	0.202584207
PDCD1LG2	rs4742122	3.67E-301	0.798099173	8.10E-302	0.176154102	0.025746725	3.16E-308	0.0233609	0.201900827
CTSS	rs141023117	4.66E-302	0.798133964	8.46E-303	0.144784321	0.057081715	3.16E-308	0.00507399	0.201866036
CTSS	rs146859881	4.90E-302	0.798218893	8.87E-303	0.144418216	0.057362891	3.16E-308	0.00507399	0.201781107
CTSS	rs61817561	2.65E-302	0.798465609	4.88E-303	0.147116296	0.054418095	3.16E-308	0.00507399	0.201534391
CTSS	rs587755955	3.82E-302	0.79851685	6.96E-303	0.145370067	0.056113083	3.16E-308	0.00507399	0.20148315
CTSS	rs41266899	3.66E-302	0.79874981	6.66E-303	0.145361202	0.055888988	3.16E-308	0.00507399	0.20125019

CTSS	rs72700829	6.22E-302	0.798942345	1.10E-302	0.141637991	0.059419664	3.16E-308	0.00507399	0.201057655
PDCD1LG2	rs148201597	1.28E-90	0.803052519	3.11E-91	0.194972635	0.001974846	1.83E-97	0.892609	0.196947481
CTSS	rs114221421	1.29E-302	0.805283014	2.37E-303	0.147569557	0.047147429	3.16E-308	0.00507399	0.194716986
CTSS	rs115539008	1.47E-302	0.805317572	2.66E-303	0.145918694	0.048763734	3.16E-308	0.00507399	0.194682428
PDCD1LG2	rs183729173	2.80E-301	0.805925333	5.82E-302	0.167283201	0.026791467	3.16E-308	0.0224864	0.194074668
PDCD1LG2	rs185501274	2.25E-301	0.807904091	4.62E-302	0.165765287	0.026330623	3.16E-308	0.0224864	0.19209591
CTSS	rs6668850	1.92E-300	0.811347902	3.35E-301	0.141667607	0.046984491	3.16E-308	0.0127359	0.188652098
AOC3	rs12948911	4.75E-302	0.813470594	6.21E-303	0.106149159	0.080380247	3.16E-308	0.00302134	0.186529406
AOC3	rs550197631	3.71E-302	0.815846481	4.50E-303	0.098802271	0.085351248	3.16E-308	0.00168791	0.184153519
AOC3	rs143662977	3.62E-302	0.818424102	4.35E-303	0.098239668	0.08333623	3.16E-308	0.00168791	0.181575898
CTSS	rs41271965	1.06E-302	0.822771392	1.68E-303	0.130539265	0.046689343	3.16E-308	0.00507399	0.177228608
TCN2	rs191176689	3.40E-301	0.82382997	6.66E-302	0.161490869	0.014679161	3.16E-308	0.0409723	0.17617003
TCN2	rs185479315	3.40E-301	0.823890503	6.66E-302	0.161429257	0.01468024	3.16E-308	0.0409723	0.176109497
AOC3	rs545983765	5.85E-302	0.826881882	6.74E-303	0.095284983	0.077833136	3.16E-308	0.00387606	0.173118119
PDCD1LG2	rs76173761	3.32E-68	0.828025643	6.79E-69	0.169192594	0.002781763	7.83E-75	0.532086	0.171974357
CTSS	rs587723357	2.87E-300	0.83140022	4.80E-301	0.139229529	0.029370251	3.16E-308	0.0453858	0.16859978
CTSS	rs151171909	3.88E-300	0.831854167	6.47E-301	0.138627679	0.029518154	3.16E-308	0.0453858	0.168145833

MMP3	rs145626826	1.07E-302	0.832373977	1.97E-303	0.153334563	0.01429146	3.16E-308	0.0161741	0.167626023
MMP3	rs12808148	1.07E-302	0.832717587	1.97E-303	0.152985053	0.01429736	3.16E-308	0.0161741	0.167282413
MMP3	rs1144393	1.07E-302	0.832794536	1.97E-303	0.152906783	0.014298681	3.16E-308	0.0161741	0.167205464
MICB	rs573163339	1.96E-303	0.834348127	3.46E-304	0.147487448	0.018164425	3.16E-308	0.00196612	0.165651873
MMP3	rs117470978	4.51E-302	0.835833317	8.46E-303	0.156956551	0.007210132	3.16E-308	0.0250749	0.164166683
MMP3	rs192030279	1.08E-302	0.836368313	1.92E-303	0.149271646	0.014360041	3.16E-308	0.0161741	0.163631687
MMP3	rs182844815	1.08E-302	0.83656544	1.92E-303	0.149071135	0.014363425	3.16E-308	0.0161741	0.16343456
MMP3	rs187772022	1.21E-302	0.836902877	2.14E-303	0.148030697	0.015066426	3.16E-308	0.0161741	0.163097123
MMP3	rs532050154	3.96E-302	0.838213038	7.29E-303	0.154107257	0.007679705	3.16E-308	0.0250749	0.161786962
MMP3	rs146605342	8.15E-302	0.838926949	1.50E-302	0.15397575	0.007097301	3.16E-308	0.0250749	0.161073051
BRSK2	rs72847992	5.36E-179	0.839059432	4.50E-180	0.070340948	0.09059962	5.24E-186	0.00655028	0.160940568
CPOX	rs137960554	1.26E-07	0.839459426	2.16E-08	0.144382314	0.016158112	1.40E-13	0.697347	0.160540426
MICB	rs537177733	2.49E-303	0.84073912	4.25E-304	0.14330733	0.01595355	3.16E-308	0.00196612	0.15926088
MICB	rs115978492	2.58E-303	0.841212226	4.59E-304	0.149593892	0.009193881	3.16E-308	0.00578229	0.158787773
MMP3	rs181828286	8.94E-302	0.842757699	1.61E-302	0.151746854	0.005495447	3.16E-308	0.0660846	0.157242301
CHAD	rs144030994	5.92E-235	0.846973992	4.28E-236	0.061100976	0.091925032	1.68E-257	0.0357495	0.153026008
CHAD	rs9907393	5.93E-235	0.848431734	4.17E-236	0.05948502	0.092083246	1.68E-257	0.0357495	0.151568266

EFNA4	rs189497019	2.00E-48	0.849319372	2.01E-49	0.085392754	0.065287873	1.67E-62	0.730898	0.150680627
EFNA4	rs72704114	2.00E-48	0.849375203	2.01E-49	0.085332632	0.065292165	1.67E-62	0.730898	0.150624797
MICB	rs79342353	5.79E-303	0.850676017	9.37E-304	0.137568782	0.011755202	3.16E-308	0.00578229	0.149323984
SOD3	rs73252516	2.54E-60	0.851695048	1.43E-61	0.04794234	0.100362612	1.43E-88	0.131097	0.148304952
EFNA4	rs150010313	2.00E-48	0.852111583	1.94E-49	0.082385904	0.065502513	1.67E-62	0.730898	0.147888417
PDCD1LG2	rs546249299	1.04E-302	0.852844115	1.73E-303	0.141831731	0.005324154	3.16E-308	0.0224864	0.147155885
PDCD1LG2	rs76508446	1.04E-302	0.85467158	1.70E-303	0.139992857	0.005335562	3.16E-308	0.0224864	0.145328419
PDCD1LG2	rs10975172	1.04E-302	0.854832705	1.70E-303	0.139830726	0.005336568	3.16E-308	0.0224864	0.145167294
CTSS	rs9325978	1.10E-302	0.857779594	1.20E-303	0.093613312	0.048607094	3.16E-308	0.00507399	0.142220406
NME3	rs193272659	5.96E-75	0.861519512	4.50E-76	0.065017906	0.073462583	1.75E-90	0.350011	0.138480489
PIK3AP1	rs41317268	4.37E-129	0.861564167	6.75E-130	0.133242827	0.005193006	6.50E-136	0.233944	0.138435833
PIK3AP1	rs61858553	4.37E-129	0.86178419	6.74E-130	0.133021477	0.005194333	6.50E-136	0.233944	0.13821581
CCN3	rs191251099	4.86E-27	0.862049908	6.76E-28	0.119836809	0.018113283	1.96E-33	0.744962	0.137950092
MICB	rs75509151	7.64E-303	0.862973708	1.13E-303	0.127390326	0.009635966	3.16E-308	0.0167198	0.137026292
NAGPA	rs150975275	1.66E-300	0.864564359	1.06E-301	0.055045791	0.08038985	3.16E-308	0.0100781	0.135435641
NAGPA	rs2972282	1.66E-300	0.86463124	1.06E-301	0.054972691	0.080396069	3.16E-308	0.0100781	0.13536876
NAGPA	rs12599777	1.66E-300	0.864656159	1.06E-301	0.054945455	0.080398386	3.16E-308	0.0100781	0.135343841

CTSS	rs587648799	1.11E-302	0.864706242	1.11E-303	0.086277878	0.04901588	3.16E-308	0.00507399	0.135293758
NAGPA	rs78705060	1.66E-300	0.864730064	1.05E-301	0.054864677	0.080405258	3.16E-308	0.0100781	0.135269935
NAGPA	rs149654487	1.66E-300	0.8655214	1.04E-301	0.053999761	0.080478839	3.16E-308	0.0100781	0.1344786
BMPER	rs1861365	5.09E-50	0.865917001	6.90E-51	0.117315773	0.016767227	1.42E-56	0.0492697	0.134083
CTSS	rs10305663	1.11E-302	0.86727833	1.07E-303	0.08354278	0.049178889	3.16E-308	0.00507399	0.132721669
NAGPA	rs118094816	1.66E-300	0.867518488	9.95E-302	0.051816978	0.080664534	3.16E-308	0.0100781	0.132481512
CTSS	rs587621222	1.27E-299	0.869296271	1.47E-300	0.100801742	0.029901987	3.16E-308	0.0498919	0.130703729
CTSS	rs187722388	1.12E-302	0.870916224	1.02E-303	0.079698601	0.049385176	3.16E-308	0.00507399	0.129083777
ASRGL1	rs12798658	1.06E-302	0.871279202	1.43E-303	0.117409487	0.011311311	3.16E-308	0.175168	0.128720798
SERPINF2	rs111926537	8.84E-101	0.871578942	1.26E-101	0.124564124	0.003856934	9.91E-108	0.390576	0.128421058
SERPINF2	rs11078597	8.84E-101	0.871786798	1.26E-101	0.124355349	0.003857853	9.91E-108	0.390576	0.128213202
CTSS	rs181705031	1.12E-302	0.873590724	9.88E-304	0.076853873	0.049555403	3.16E-308	0.00507399	0.126409276
CRYZL1	rs111332667	1.79E-59	0.873922342	6.98E-61	0.034023108	0.09205455	1.60E-66	0.0237312	0.126077658
EPO	rs11976235	2.76E-21	0.87403721	2.54E-22	0.080478741	0.04548405	1.07E-28	0.995164	0.125962791
CRYZL1	rs13050238	1.79E-59	0.875157719	6.70E-61	0.032657603	0.092184678	1.60E-66	0.0237312	0.124842281
IL1RAP	rs187070008	1.34E-302	0.87548669	1.74E-303	0.113688233	0.010825076	3.16E-308	0.0122882	0.124513309
GIPC3	rs200371465	9.97E-283	0.87567016	1.02E-283	0.089874005	0.034455835	1.09E-290	0.0351059	0.12432984

IL1RAP	rs34879831	1.34E-302	0.876346874	1.72E-303	0.112817413	0.010835712	3.16E-308	0.0122882	0.123653125
EPO	rs543967674	2.77E-21	0.876926202	2.45E-22	0.077439408	0.04563439	1.07E-28	0.995164	0.123073798
GIPC3	rs541850199	9.99E-283	0.877305421	1.00E-283	0.088174399	0.034520179	1.09E-290	0.0351059	0.122694578
CTSS	rs139755856	1.13E-302	0.87734652	9.40E-304	0.072960754	0.049692726	3.16E-308	0.00507399	0.12265348
PIK3AP1	rs56052045	4.45E-129	0.8778646	5.92E-130	0.116844144	0.005291256	6.50E-136	0.233944	0.1221354
NECTIN4	rs191299407	3.25E-300	0.877957568	2.24E-301	0.060360206	0.061682226	3.16E-308	0.014258	0.122042432
C7orf50	rs143148836	7.57E-220	0.878294965	5.77E-221	0.066888498	0.054816537	4.88E-230	0.0582264	0.121705035
NECTIN4	rs533813718	3.25E-300	0.878783684	2.21E-301	0.05947605	0.061740266	3.16E-308	0.014258	0.121216316
MFGE8	rs7174387	2.44E-302	0.878908868	1.49E-303	0.053797003	0.067294129	3.16E-308	0.00170821	0.121091132
NME3	rs370246389	6.08E-75	0.878931277	3.20E-76	0.046121423	0.0749473	1.75E-90	0.350011	0.121068723
MFGE8	rs187678386	2.44E-302	0.879080466	1.49E-303	0.053612266	0.067307268	3.16E-308	0.00170821	0.120919534
CLEC4G	rs76560987	1.53E-144	0.87923432	9.17E-146	0.052814719	0.067950961	2.30E-151	0.016826	0.12076568
CLEC4G	rs186428113	1.53E-144	0.879759152	9.08E-146	0.052249326	0.067991522	2.30E-151	0.016826	0.120240848
TNFSF14	rs62125119	4.67E-231	0.879884259	5.93E-232	0.111720146	0.008395595	1.63E-239	0.266252	0.120115741
NECTIN4	rs181003709	3.26E-300	0.880146566	2.15E-301	0.058017417	0.061836017	3.16E-308	0.014258	0.119853434
EPO	rs2075672	2.78E-21	0.880194331	2.34E-22	0.074001209	0.04580446	1.07E-28	0.995164	0.119805669
NECTIN4	rs35434391	3.26E-300	0.880501571	2.14E-301	0.05763747	0.061860958	3.16E-308	0.014258	0.119498428

CLEC4G	rs73491778	1.53E-144	0.880532183	8.93E-146	0.051416552	0.068051265	2.30E-151	0.016826	0.119467817
BRSK2	rs189976547	2.64E-302	0.880681532	2.66E-303	0.088731282	0.030587186	3.16E-308	0.00369973	0.119318468
BRSK2	rs76315991	2.68E-302	0.880843085	2.69E-303	0.088180289	0.030976626	3.16E-308	0.00369973	0.119156915
GIPC3	rs76422466	1.00E-282	0.880953963	9.61E-284	0.084382295	0.034663742	1.09E-290	0.0351059	0.119046037
GIPC3	rs34722692	1.00E-282	0.881070881	9.60E-284	0.084260776	0.034668342	1.09E-290	0.0351059	0.118929118
BRSK2	rs78131782	2.42E-302	0.881326679	2.47E-303	0.090059679	0.028613642	3.16E-308	0.00369973	0.118673321
BRSK2	rs147197826	2.38E-302	0.881383655	2.44E-303	0.090335583	0.028280763	3.16E-308	0.00369973	0.118616346
BRSK2	rs185752629	2.38E-302	0.881500363	2.43E-303	0.090215129	0.028284507	3.16E-308	0.00369973	0.118499636
CLEC4G	rs475896	1.53E-144	0.881512336	8.75E-146	0.050360649	0.068127015	2.30E-151	0.016826	0.118487664
MFGE8	rs2280213	2.45E-302	0.884182767	1.34E-303	0.048119305	0.067697928	3.16E-308	0.00170821	0.115817233
METAP1D	rs146517576	8.36E-10	0.884926021	3.78E-11	0.039895937	0.07517804	2.10E-18	0.459927	0.115073977
MFGE8	rs72759040	2.46E-302	0.885045909	1.31E-303	0.047190076	0.067764015	3.16E-308	0.00170821	0.114954091
MFGE8	rs181167783	2.46E-302	0.885381145	1.30E-303	0.046829172	0.067789683	3.16E-308	0.00170821	0.114618855
MFGE8	rs76980967	2.46E-302	0.885429383	1.30E-303	0.046777241	0.067793376	3.16E-308	0.00170821	0.114570617
SUOX	rs773107	6.22E-10	0.887240665	2.83E-11	0.040341797	0.072417538	2.03E-16	0.317151	0.112759335
METAP1D	rs62183777	8.38E-10	0.887488755	3.51E-11	0.037115611	0.075395633	2.10E-18	0.459927	0.112511244
SUOX	rs141735896	6.23E-10	0.887881397	2.79E-11	0.039648767	0.072469835	2.03E-16	0.317151	0.112118602

APRT	rs111433410	3.50E-14	0.888305931	3.27E-15	0.08287536	0.028818709	4.27E-20	0.429914	0.111694069
APRT	rs150156607	3.51E-14	0.888676961	3.25E-15	0.082492293	0.028830746	4.27E-20	0.429914	0.111323039
APRT	rs8191468	3.51E-14	0.888681474	3.25E-15	0.082487634	0.028830892	4.27E-20	0.429914	0.111318526
APRT	rs79512303	3.51E-14	0.8887662	3.25E-15	0.08240016	0.028833641	4.27E-20	0.429914	0.111233801
AGT	rs191699388	1.31E-38	0.888805478	1.08E-39	0.073283608	0.037910913	3.43E-45	0.0233158	0.111194521
PKD1	rs201158469	8.22E-34	0.889627721	6.48E-35	0.070095568	0.040276711	1.87E-41	0.816197	0.110372279
HLA-E	rs61043470	1.70E-301	0.889766542	1.92E-302	0.100344723	0.009888735	3.16E-308	0.134691	0.110233458
MICB	rs535883826	1.56E-302	0.890056343	1.73E-303	0.099039857	0.0109038	3.16E-308	0.0047596	0.109943657
MICB	rs1632863	3.70E-303	0.890174573	4.15E-304	0.09990474	0.009920687	3.16E-308	0.0046302	0.109825427
HLA-E	rs576083415	2.55E-301	0.89023646	2.85E-302	0.099555323	0.010208218	3.16E-308	0.134691	0.109763541
NECTIN4	rs546792444	3.30E-300	0.890307295	1.75E-301	0.04714283	0.062549874	3.16E-308	0.014258	0.109692704
ADGRE1	rs147811291	9.04E-153	0.892011664	1.00E-153	0.099091494	0.008896842	4.19E-166	0.474862	0.107988336
MICB	rs115884658	2.19E-302	0.892090441	2.32E-303	0.094557736	0.013351823	3.16E-308	0.0047596	0.107909559
CTSS	rs138605093	2.85E-302	0.892196376	2.11E-303	0.066172689	0.041630934	3.16E-308	0.0112585	0.107803623
HLA-E	rs61754472	3.17E-301	0.892298856	3.46E-302	0.097212079	0.010489066	3.16E-308	0.134691	0.107701145
EFNA4	rs181536617	2.71E-15	0.893274395	1.94E-16	0.063712529	0.043013076	1.86E-22	0.798205	0.106725605
ADGRE1	rs111955281	1.79E-300	0.894352887	1.97E-301	0.098510495	0.007136618	3.16E-308	0.195173	0.105647113

ADGRE1	rs61733001	1.79E-300	0.894991259	1.96E-301	0.097867029	0.007141712	3.16E-308	0.195173	0.105008741
CHAD	rs551595938	2.39E-48	0.895423874	1.08E-49	0.040394226	0.0641819	8.12E-59	0.0528652	0.104576126
IL1RAP	rs7628250	1.37E-302	0.896440287	1.41E-303	0.092472826	0.011086887	3.16E-308	0.0122882	0.103559713
ADGRE1	rs59055395	1.80E-300	0.896455154	1.93E-301	0.096391453	0.007153393	3.16E-308	0.195173	0.103544846
SMOC2	rs542654764	1.34E-177	0.89671892	1.44E-178	0.096600058	0.006681022	1.58E-184	0.247694	0.10328108
ADGRE1	rs62636889	1.80E-300	0.897527635	1.91E-301	0.095310414	0.007161951	3.16E-308	0.195173	0.102472365
TNFSF14	rs139652996	4.77E-231	0.89985428	4.86E-232	0.091559576	0.008586144	1.63E-239	0.266252	0.10014572
BOLA2B	rs111894927	1.05E-14	0.900100311	2.63E-16	0.022409866	0.077489823	3.28E-21	0.0221473	0.099899689
SMOC2	rs143806544	3.42E-174	0.900649096	3.63E-175	0.095503015	0.00384789	1.42E-180	0.442295	0.099350905
MMP3	rs2155053	1.27E-302	0.901051307	1.16E-303	0.082479885	0.016468808	3.16E-308	0.0161741	0.098948693
TNFSF14	rs72990326	4.78E-231	0.901281661	4.78E-232	0.090118576	0.008599763	1.63E-239	0.266252	0.098718339
EPO	rs555237664	1.09E-11	0.901375968	6.11E-13	0.050456648	0.048167384	1.09E-18	0.963592	0.098624032
TNFSF14	rs191776679	4.79E-231	0.901996527	4.74E-232	0.089396889	0.008606584	1.63E-239	0.266252	0.098003473
TNFSF14	rs344560	4.79E-231	0.902011283	4.74E-232	0.089381992	0.008606725	1.63E-239	0.266252	0.097988717
TNFSF14	rs8113119	4.79E-231	0.902344845	4.73E-232	0.089045248	0.008609908	1.63E-239	0.266252	0.097655156
HLA-E	rs2530715	1.68E-302	0.903103858	1.68E-303	0.090207033	0.006689108	3.16E-308	0.215442	0.096896141
HLA-E	rs114000484	1.68E-302	0.903107986	1.68E-303	0.090202875	0.006689139	3.16E-308	0.215442	0.096892014

TCN2	rs4820867	1.33E-302	0.903470708	1.11E-303	0.075183354	0.021345938	3.16E-308	0.00171945	0.096529292
SUMF2	rs117086105	3.99E-296	0.903503988	1.40E-297	0.031675983	0.064820029	3.16E-308	0.0325604	0.096496012
SUMF2	rs4245575	3.99E-296	0.903524353	1.40E-297	0.031654158	0.064821489	3.16E-308	0.0325604	0.096475647
LY75	rs144577910	5.08E-302	0.903547594	4.48E-303	0.079599986	0.016852419	3.16E-308	0.0196006	0.096452405
TCN2	rs575116744	1.33E-302	0.90383501	1.10E-303	0.074810445	0.021354545	3.16E-308	0.00171945	0.09616499
TCN2	rs2267158	1.33E-302	0.903929205	1.10E-303	0.074714025	0.021356771	3.16E-308	0.00171945	0.096070796
SUMF2	rs149087624	3.99E-296	0.903936281	1.38E-297	0.031212677	0.064851042	3.16E-308	0.0325604	0.096063719
LY75	rs147074648	6.17E-302	0.903976125	5.43E-303	0.079532429	0.016491446	3.16E-308	0.0242019	0.096023875
MMP3	rs145610646	1.73E-121	0.904056879	1.66E-122	0.087162373	0.008780748	1.58E-128	0.0950452	0.095943121
BCAM	rs10409076	2.54E-87	0.904269041	1.88E-88	0.067022316	0.028708642	1.87E-95	0.121359	0.095730958
ENTR1	rs11145904	1.40E-176	0.904849266	1.40E-177	0.090328795	0.004821939	5.89E-184	0.521146	0.095150734
SERPINF2	rs149889520	9.18E-101	0.904889797	9.24E-102	0.091105861	0.004004342	9.91E-108	0.390576	0.095110203
CSF3R	rs148363675	7.21E-186	0.905137316	3.94E-187	0.049395062	0.045467622	1.86E-199	0.0322552	0.094862684
ANXA2	rs145847883	2.28E-301	0.905593191	2.27E-302	0.090149336	0.004257473	3.16E-308	0.231541	0.094406809
CDHR5	rs370247985	2.90E-302	0.905674868	2.71E-303	0.08462057	0.009704562	3.16E-308	0.0228786	0.094325132
BRSK2	rs368114023	2.35E-302	0.905746236	1.71E-303	0.065997625	0.028256139	3.16E-308	0.00369973	0.094253764
SERPINE2	rs191234149	1.95E-302	0.905974869	1.92E-303	0.089117796	0.004907335	3.16E-308	0.0568696	0.094025131

MMP3	rs72971591	1.73E-121	0.906029689	1.63E-122	0.085170402	0.008799909	1.58E-128	0.0950452	0.093970311
CRYZL1	rs200823066	7.75E-58	0.906072619	6.52E-59	0.076215972	0.017711409	6.25E-65	0.243464	0.093927381
ENTR1	rs200698119	1.40E-176	0.906096263	1.38E-177	0.089075152	0.004828585	5.89E-184	0.521146	0.093903737
TNFRSF19	rs61947005	1.74E-239	0.906177298	1.40E-240	0.07278054	0.021042161	1.32E-246	0.0348329	0.093822701
CDHR5	rs148332379	2.91E-302	0.906363098	2.69E-303	0.083924965	0.009711937	3.16E-308	0.0228786	0.093636902
CDHR5	rs531412831	2.91E-302	0.906367797	2.69E-303	0.083920216	0.009711987	3.16E-308	0.0228786	0.093632203
TNFRSF19	rs184751212	1.75E-239	0.906401004	1.40E-240	0.072551641	0.021047356	1.32E-246	0.0348329	0.093598997
CDHR5	rs12363914	2.91E-302	0.906405394	2.69E-303	0.083882217	0.00971239	3.16E-308	0.0228786	0.093594607
TNFRSF12A	rs183549801	3.39E-12	0.906531869	3.04E-13	0.081352943	0.012115188	2.35E-18	0.19165	0.093468131
MMP3	rs118049989	1.73E-121	0.906565357	1.62E-122	0.084629531	0.008805112	1.58E-128	0.0950452	0.093434643
CDHR5	rs936461	2.91E-302	0.906567631	2.68E-303	0.08371824	0.009714128	3.16E-308	0.0228786	0.093432368
CDHR5	rs201081599	2.91E-302	0.906602832	2.68E-303	0.083682662	0.009714505	3.16E-308	0.0228786	0.093397167
TNFRSF12A	rs74758164	3.39E-12	0.906880885	3.03E-13	0.080999262	0.012119852	2.35E-18	0.19165	0.093119114
TNFRSF12A	rs55699988	3.39E-12	0.90700052	3.02E-13	0.080878029	0.012121451	2.35E-18	0.19165	0.09299948
ASRGL1	rs55906750	1.10E-302	0.907067768	9.88E-304	0.081156298	0.011775933	3.16E-308	0.175168	0.092932231
CSF3R	rs3917922	7.23E-186	0.907238197	3.76E-187	0.047188648	0.045573155	1.86E-199	0.0322552	0.092761803
TCN2	rs191049731	1.33E-302	0.90737291	1.04E-303	0.071262043	0.021365046	3.16E-308	0.00171945	0.092627089

CSF3R	rs142999683	7.23E-186	0.907460899	3.75E-187	0.046954759	0.045584342	1.86E-199	0.0322552	0.092539101
CSF3R	rs3918018	7.23E-186	0.907718585	3.72E-187	0.046684129	0.045597286	1.86E-199	0.0322552	0.092281415
TNFRSF19	rs61947047	1.75E-239	0.90787386	1.37E-240	0.071044583	0.021081557	1.32E-246	0.0348329	0.09212614
CSF3R	rs3917999	7.24E-186	0.907998329	3.70E-187	0.046390333	0.045611339	1.86E-199	0.0322552	0.092001672
TCN2	rs190188440	5.38E-302	0.908039971	2.79E-303	0.047040683	0.044919345	3.16E-308	0.00171945	0.091960028
CDHR5	rs58761034	2.91E-302	0.908121473	2.63E-303	0.082147749	0.009730778	3.16E-308	0.0228786	0.091878527
SERPINE2	rs573829890	1.96E-302	0.908321842	1.87E-303	0.08675811	0.004920048	3.16E-308	0.0568696	0.091678158
TACSTD2	rs77740907	7.40E-303	0.909142598	6.09E-304	0.074777304	0.016080099	3.16E-308	0.031209	0.090857403
CSF3R	rs143009827	7.25E-186	0.909183858	3.60E-187	0.045145251	0.045670891	1.86E-199	0.0322552	0.090816142
ENTR1	rs149384840	1.41E-176	0.909249466	1.33E-177	0.085905146	0.004845388	5.89E-184	0.521146	0.090750534
ENTR1	rs35873563	1.41E-176	0.909562421	1.32E-177	0.085590523	0.004847056	5.89E-184	0.521146	0.090437579
RELT	rs113689386	1.34E-302	0.910625674	6.69E-304	0.045304807	0.044069518	3.16E-308	0.00860221	0.089374325
ADAMTS16	rs6555331	1.49E-54	0.910734501	1.41E-55	0.086378475	0.002887024	5.30E-61	0.53419	0.089265499
LY75	rs537198250	1.99E-302	0.910759115	1.70E-303	0.077445338	0.011795547	3.16E-308	0.0196006	0.089240885
SERPINE2	rs76338368	1.96E-302	0.910816662	1.81E-303	0.084249776	0.004933562	3.16E-308	0.0568696	0.089183338
TCN2	rs183180076	3.88E-302	0.911036021	2.01E-303	0.047137646	0.041826333	3.16E-308	0.00171945	0.088963979
ADGRD1	rs12369478	2.86E-297	0.911270771	2.10E-298	0.067141259	0.02158797	3.16E-308	0.100266	0.088729229

SLA2	rs113102870	2.40E-51	0.911422157	7.25E-53	0.027520215	0.061057628	2.28E-60	0.141319	0.088577843
DEFB104B	rs139329620	4.93E-25	0.911513103	3.88E-26	0.071748348	0.016738549	1.71E-31	0.282379	0.088486897
ADGRD1	rs116945914	2.86E-297	0.911657583	2.09E-298	0.066745283	0.021597134	3.16E-308	0.100266	0.088342417
PCDH9	rs184886175	7.68E-30	0.911747262	3.72E-31	0.044109709	0.044143029	1.92E-36	0.0142709	0.088252738
HLA-E	rs374228048	3.73E-302	0.911808446	3.20E-303	0.078221435	0.009970119	3.16E-308	0.106809	0.088191554
LY75	rs184156996	1.79E-302	0.911884723	1.51E-303	0.07693433	0.011180947	3.16E-308	0.0196006	0.088115277
SERPINE2	rs560855311	1.96E-302	0.912092886	1.79E-303	0.08296664	0.004940475	3.16E-308	0.0568696	0.087907115
ADAMTS16	rs16875264	7.42E-38	0.912260268	6.69E-39	0.082246878	0.005492855	4.78E-44	0.873922	0.087739733
PTPRH	rs187217214	8.06E-302	0.912306338	5.33E-303	0.060293125	0.027400536	3.16E-308	0.0052342	0.087693661
ADGRD1	rs140409584	2.86E-297	0.912317439	2.07E-298	0.066069796	0.021612766	3.16E-308	0.100266	0.087682562
AGT	rs7553239	2.41E-302	0.912474838	2.03E-303	0.076745434	0.010779728	3.16E-308	0.0130737	0.087525162
ST6GAL1	rs4686837	2.80E-106	0.912602061	1.46E-107	0.047388182	0.040009757	2.90E-113	0.0175542	0.087397939
TCN2	rs571138277	1.91E-302	0.912627049	1.60E-303	0.076419978	0.010952973	3.16E-308	0.0146187	0.087372951
SERPINE2	rs7602769	1.96E-302	0.912730003	1.77E-303	0.082326071	0.004943926	3.16E-308	0.0568696	0.087269997
IL32	rs111497883	3.55E-208	0.912770841	3.16E-209	0.081268902	0.005960257	6.71E-216	0.569552	0.087229159
IL32	rs45499297	3.55E-208	0.91278025	3.16E-209	0.081259431	0.005960319	6.71E-216	0.569552	0.08721975
PTPRH	rs78021046	8.07E-302	0.912896673	5.28E-303	0.05968506	0.027418267	3.16E-308	0.0052342	0.087103327

ADAMTS16	rs16875213	7.43E-38	0.912961588	6.63E-39	0.081541335	0.005497077	4.78E-44	0.873922	0.087038412
TACSTD2	rs141801895	1.02E-50	0.913115631	8.64E-52	0.077277048	0.009607321	2.81E-58	0.562337	0.086884369
FN1	rs62182927	7.72E-302	0.913250829	6.10E-303	0.072225038	0.014524133	3.16E-308	0.0116113	0.086749171
TCN2	rs191118935	3.01E-302	0.913314941	2.49E-303	0.075388087	0.011296971	3.16E-308	0.0220714	0.086685058
IL32	rs141583132	3.55E-208	0.913396875	3.13E-209	0.08063878	0.005964345	6.71E-216	0.569552	0.086603125
F3	rs6666213	1.05E-138	0.913614216	9.21E-140	0.080160358	0.006225426	7.76E-145	0.166331	0.086385784
F3	rs11586546	1.05E-138	0.913794724	9.18E-140	0.07997862	0.006226656	7.76E-145	0.166331	0.086205276
ASRGL1	rs10897270	1.11E-302	0.913834555	9.04E-304	0.074301663	0.011863782	3.16E-308	0.175168	0.086165445
TCN2	rs183696935	3.49E-302	0.914088912	1.76E-303	0.045925416	0.039985672	3.16E-308	0.00171945	0.085911088
ASRGL1	rs2513045	1.11E-302	0.914325275	8.98E-304	0.073804572	0.011870153	3.16E-308	0.175168	0.085674725
TCN2	rs538727957	2.45E-302	0.9143846	2.03E-303	0.075711857	0.009903543	3.16E-308	0.0220714	0.0856154
PDCD1LG2	rs143929517	1.11E-302	0.914554576	9.70E-304	0.079736023	0.005709401	3.16E-308	0.0224864	0.085445424
CAPS	rs147363394	4.58E-47	0.914651927	3.98E-48	0.079339581	0.006008493	1.32E-53	0.306802	0.085348074
CDHR5	rs142202229	2.93E-302	0.914781685	2.42E-303	0.075416171	0.009802144	3.16E-308	0.0228786	0.085218315
BRSK2	rs56325034	2.46E-302	0.914831495	1.65E-303	0.061293117	0.023875388	3.16E-308	0.00471965	0.085168505
PTPRH	rs45493898	8.08E-302	0.914874612	5.10E-303	0.057647716	0.027477673	3.16E-308	0.0052342	0.085125389
PDCD1LG2	rs4143815	1.11E-302	0.915041526	9.64E-304	0.079246033	0.005712441	3.16E-308	0.0224864	0.084958474

CAPS	rs274786	4.58E-47	0.915047838	3.96E-48	0.078941068	0.006011093	1.32E-53	0.306802	0.084952161
CAPS	rs117517033	4.58E-47	0.915081989	3.95E-48	0.078906694	0.006011318	1.32E-53	0.306802	0.084918012
PDCD1LG2	rs565656171	1.11E-302	0.915221469	9.62E-304	0.079064967	0.005713564	3.16E-308	0.0224864	0.084778531
TCN2	rs189138786	4.76E-302	0.915285715	3.88E-303	0.074537268	0.010177017	3.16E-308	0.0599363	0.084714285
DEFB104B	rs185881426	4.95E-25	0.915406973	3.67E-26	0.067782973	0.016810054	1.71E-31	0.282379	0.084593027
PTPRH	rs184973746	8.09E-302	0.915482208	5.04E-303	0.05702187	0.027495922	3.16E-308	0.0052342	0.084517792
F3	rs12132188	1.05E-138	0.915541138	8.98E-140	0.078220306	0.006238556	7.76E-145	0.166331	0.084458862
LY75	rs554723881	1.20E-302	0.915567771	9.82E-304	0.074900656	0.009531574	3.16E-308	0.0196006	0.08443223
PTPRH	rs564216512	8.09E-302	0.915742503	5.02E-303	0.056753758	0.027503739	3.16E-308	0.0052342	0.084257497
SOD3	rs2284659	1.46E-301	0.915880734	8.13E-303	0.050926568	0.033192698	3.16E-308	0.00915019	0.084119266
PTPRH	rs116877753	8.09E-302	0.916007292	4.99E-303	0.056481015	0.027511692	3.16E-308	0.0052342	0.083992707
THBD	rs13044901	1.72E-18	0.916207016	1.52E-19	0.080739696	0.003053289	2.31E-23	0.697988	0.083792985
SOD3	rs182732759	1.46E-301	0.916323039	8.06E-303	0.050468234	0.033208727	3.16E-308	0.00915019	0.083676961
FBLN2	rs149340876	4.45E-51	0.916379118	2.12E-52	0.043626819	0.039994063	9.25E-63	0.922358	0.083620882
SOD3	rs182701447	1.46E-301	0.916454289	8.04E-303	0.050332227	0.033213484	3.16E-308	0.00915019	0.083545711
PTPRH	rs61704307	8.10E-302	0.91653001	4.95E-303	0.055942598	0.027527392	3.16E-308	0.0052342	0.08346999
SOD3	rs74870948	1.46E-301	0.91692879	7.96E-303	0.04984053	0.033230681	3.16E-308	0.00915019	0.083071211

FGFBP3	rs61754655	5.51E-163	0.916937065	2.11E-164	0.035107795	0.04795514	3.18E-170	0.0165128	0.083062935
RELT	rs180795055	1.02E-302	0.916947556	5.29E-304	0.047512614	0.03553983	3.16E-308	0.00860221	0.083052444
CDHR5	rs147270893	2.94E-302	0.917126582	2.34E-303	0.073046148	0.00982727	3.16E-308	0.0228786	0.082873418
BCAM	rs559111606	7.53E-33	0.917170134	6.25E-34	0.0760797	0.006750166	1.99E-39	0.185284	0.082829866
FGFBP3	rs56826659	5.51E-163	0.917170331	2.10E-164	0.03486233	0.04796734	3.18E-170	0.0165128	0.08282967
MICB	rs9296004	3.21E-302	0.917219904	2.31E-303	0.065776858	0.017003238	3.16E-308	0.0047596	0.082780096
RELT	rs7928848	1.01E-302	0.917335976	5.22E-304	0.047515171	0.035148853	3.16E-308	0.00860221	0.082664024
BCAM	rs8100120	8.74E-226	0.917389203	6.36E-227	0.066708222	0.015902575	3.29E-235	0.289236	0.082610797
SOD3	rs191111481	1.46E-301	0.91752717	7.86E-303	0.049220464	0.033252367	3.16E-308	0.00915019	0.082472831
BCAM	rs1135062	8.75E-226	0.917547655	6.34E-227	0.066547024	0.015905322	3.29E-235	0.289236	0.082452346
SOD3	rs144229051	1.46E-301	0.917607699	7.85E-303	0.049137016	0.033255285	3.16E-308	0.00915019	0.082392301
HLA-E	rs3094054	3.53E-302	0.917686193	2.78E-303	0.07221925	0.010094556	3.16E-308	0.106809	0.082313806
EFNA4	rs548043641	2.79E-15	0.917766236	1.16E-16	0.038041353	0.044192411	1.86E-22	0.798205	0.082233764
TCN2	rs188669062	1.32E-302	0.917838325	8.74E-304	0.060614148	0.021547526	3.16E-308	0.00171945	0.082161674
ST6GAL1	rs4686807	2.82E-106	0.918227546	1.28E-107	0.041516067	0.040256387	2.90E-113	0.0175542	0.081772454
SUMF2	rs112462338	3.08E-296	0.918506502	1.22E-297	0.036470835	0.045022663	3.16E-308	0.0325604	0.081493498
SMPD1	rs11040949	6.72E-09	0.918559635	4.81E-10	0.065740751	0.015699607	5.53E-16	0.35421	0.081440358

CSF1R	rs112770824	4.13E-241	0.918600218	2.39E-242	0.053016496	0.028383286	2.79E-262	0.463089	0.081399782
PTPRH	rs201456668	1.05E-301	0.918696755	5.48E-303	0.047735324	0.033567921	3.16E-308	0.0052342	0.081303245
NCAM1	rs10749999	1.95E-54	0.918742592	1.67E-55	0.078649156	0.002608251	1.27E-60	0.572817	0.081257407
TCN2	rs141948589	1.31E-302	0.91876639	8.49E-304	0.059707753	0.021525858	3.16E-308	0.00171945	0.081233611
BCAM	rs28399654	8.76E-226	0.918834678	6.22E-227	0.065237691	0.015927632	3.29E-235	0.289236	0.081165323
PTPRH	rs111814558	8.12E-302	0.918919766	4.73E-303	0.053481068	0.027599166	3.16E-308	0.0052342	0.081080234
TCN2	rs56142640	1.31E-302	0.919052657	8.45E-304	0.059414778	0.021532565	3.16E-308	0.00171945	0.080947343
TCN2	rs139909952	2.64E-301	0.919171116	2.15E-302	0.074789518	0.006039366	3.16E-308	0.0404027	0.080828884
FN1	rs147831535	7.77E-302	0.919359053	5.58E-303	0.066019671	0.014621276	3.16E-308	0.0116113	0.080640947
AAMDC	rs12419340	2.83E-298	0.919398954	2.24E-299	0.07282904	0.007772006	3.16E-308	0.73853	0.080601046
FN1	rs1250248	7.77E-302	0.919421978	5.57E-303	0.065955745	0.014622277	3.16E-308	0.0116113	0.080578022
HS1BP3	rs137999973	1.79E-12	0.919532692	8.97E-14	0.045923904	0.034543403	1.32E-19	0.560104	0.080467307
TCN2	rs572288473	1.31E-302	0.91955415	8.38E-304	0.058901536	0.021544314	3.16E-308	0.00171945	0.08044585
AKR1B1	rs563239541	4.63E-13	0.919692843	2.10E-14	0.041798423	0.038508734	3.66E-20	0.0936138	0.080307157
TNFRSF10C	rs62501097	7.66E-301	0.919746854	4.03E-302	0.048347521	0.031905625	3.16E-308	0.0197989	0.080253146
NCAM1	rs184493043	1.52E-30	0.919808544	1.28E-31	0.077519999	0.002671457	9.36E-37	0.711659	0.080191456
CDHR5	rs140634178	3.22E-301	0.919943549	2.32E-302	0.066223035	0.013833416	3.16E-308	0.0612802	0.080056451

CDHR5	rs143255211	1.11E-301	0.920071444	8.47E-303	0.070213235	0.009715321	3.16E-308	0.0612802	0.079928556
CD27	rs25680	7.50E-246	0.920192465	3.84E-247	0.047080947	0.032726589	7.18E-253	0.878453	0.079807536
TCN2	rs185788920	1.31E-302	0.920206525	8.28E-304	0.058233877	0.021559599	3.16E-308	0.00171945	0.079793476
AKR1B1	rs71535769	4.63E-13	0.920219539	2.08E-14	0.041249674	0.038530788	3.66E-20	0.0936138	0.079780462
CD27	rs11064153	7.50E-246	0.92036948	3.83E-247	0.046897636	0.032732884	7.18E-253	0.878453	0.07963052
FN1	rs188547985	7.78E-302	0.920423739	5.49E-303	0.064938052	0.014638209	3.16E-308	0.0116113	0.079576261
AOC3	rs76305463	8.85E-302	0.920462832	2.96E-303	0.030709116	0.048828052	3.16E-308	0.00168791	0.079537168
CDHR5	rs186146426	7.81E-301	0.920548113	5.49E-302	0.064701509	0.014750379	3.16E-308	0.0670456	0.079451888
CDHR5	rs113725585	1.16E-301	0.92057938	8.77E-303	0.069807849	0.009612771	3.16E-308	0.0612802	0.07942062
AGT	rs11568019	2.43E-302	0.920706363	1.81E-303	0.068416665	0.010876973	3.16E-308	0.0130737	0.079293638
CD27	rs7132503	7.51E-246	0.920765632	3.79E-247	0.046487394	0.032746973	7.18E-253	0.878453	0.079234367
AGT	rs56073403	2.43E-302	0.920805696	1.81E-303	0.068316158	0.010878146	3.16E-308	0.0130737	0.079194304
AGT	rs61731497	2.43E-302	0.920838052	1.81E-303	0.06828342	0.010878528	3.16E-308	0.0130737	0.079161948
AGT	rs12046036	2.43E-302	0.920912747	1.80E-303	0.068207842	0.010879411	3.16E-308	0.0130737	0.079087253
C7orf50	rs117928563	8.13E-302	0.921042859	6.72E-303	0.07615387	0.002803271	3.16E-308	0.519769	0.078957141
AGT	rs185247805	2.44E-302	0.921117907	1.80E-303	0.068000258	0.010881835	3.16E-308	0.0130737	0.078882093
IL32	rs35057952	3.58E-208	0.921302995	2.82E-209	0.072681034	0.006015971	6.71E-216	0.569552	0.078697005

BMPER	rs188316828	6.82E-107	0.921320361	5.35E-108	0.07223813	0.00644151	5.27E-114	0.306719	0.07867964
BMPER	rs75306052	6.82E-107	0.921369794	5.34E-108	0.072188351	0.006441855	5.27E-114	0.306719	0.078630206
C7orf50	rs12701220	2.86E-302	0.921738708	2.17E-303	0.069860535	0.008400757	3.16E-308	0.0549794	0.078261292
BMPER	rs191810047	6.82E-107	0.92186486	5.31E-108	0.071689824	0.006445317	5.27E-114	0.306719	0.078135141
PSAPL1	rs538902686	2.94E-302	0.922031701	2.23E-303	0.069909619	0.00805868	3.16E-308	0.00966207	0.077968299
BRSK2	rs56321310	2.68E-302	0.92205763	1.73E-303	0.059391615	0.018550754	3.16E-308	0.00710804	0.077942369
FBLN2	rs79270273	5.38E-231	0.922154798	2.92E-232	0.049952775	0.027892427	2.44E-252	0.214941	0.077845202
RNASE1	rs56216219	1.37E-156	0.922255947	1.10E-157	0.07437606	0.003367992	1.05E-163	0.436277	0.077744052
SOD3	rs185376677	6.66E-34	0.922266183	3.35E-35	0.046320476	0.03141334	5.81E-43	0.882475	0.077733816
BMPER	rs201897458	6.83E-107	0.92229536	5.27E-108	0.071256314	0.006448326	5.27E-114	0.306719	0.07770464
TNFRSF10C	rs183881733	7.68E-301	0.922347972	3.80E-302	0.045656171	0.031995857	3.16E-308	0.0197989	0.077652028
SERPINE2	rs34195309	9.93E-29	0.922416196	7.64E-30	0.070928055	0.006655748	3.35E-35	0.462351	0.077583803
PSAPL1	rs36076856	2.94E-302	0.922423467	2.22E-303	0.069514429	0.008062104	3.16E-308	0.00966207	0.077576533
CD27	rs530402699	7.52E-246	0.922460986	3.65E-247	0.044731746	0.032807268	7.18E-253	0.878453	0.077539014
FBLN2	rs557175061	5.39E-231	0.922464922	2.90E-232	0.04963327	0.027901807	2.44E-252	0.214941	0.077535077
FBLN2	rs77218549	5.39E-231	0.922526543	2.90E-232	0.049569785	0.027903671	2.44E-252	0.214941	0.077473456
TNFRSF10C	rs78082767	7.68E-301	0.922579355	3.78E-302	0.045416761	0.032003883	3.16E-308	0.0197989	0.077420644

FBLN2	rs79945387	5.39E-231	0.9227352	2.88E-232	0.049354818	0.027909982	2.44E-252	0.214941	0.0772648
TNFRSF10C	rs4871849	7.68E-301	0.922749512	3.77E-302	0.045240702	0.032009786	3.16E-308	0.0197989	0.077250488
TNFRSF10C	rs11987606	7.69E-301	0.922864275	3.76E-302	0.045121957	0.032013767	3.16E-308	0.0197989	0.077135724
C7orf50	rs551761454	2.86E-302	0.922934884	2.13E-303	0.068653457	0.008411659	3.16E-308	0.0549794	0.077065116
TNFRSF10C	rs76970501	7.69E-301	0.92307737	3.74E-302	0.04490147	0.032021159	3.16E-308	0.0197989	0.076922629
TNFRSF10C	rs149683200	7.69E-301	0.923108497	3.74E-302	0.044869264	0.032022239	3.16E-308	0.0197989	0.076891503
SLA2	rs113284352	2.26E-48	0.92313747	6.12E-50	0.024946082	0.051916448	1.95E-57	0.177033	0.07686253
AKR1B1	rs535642059	2.06E-44	0.923151012	9.36E-46	0.041819948	0.03502904	1.49E-52	0.15023	0.076848988
AGT	rs78497703	2.44E-302	0.923170805	1.74E-303	0.065923108	0.010906087	3.16E-308	0.0130737	0.076829195
C7orf50	rs114958928	2.86E-302	0.923293306	2.12E-303	0.068291769	0.008414926	3.16E-308	0.0549794	0.076706695
CDHR5	rs188021865	1.45E-301	0.923381129	7.91E-303	0.050282291	0.02633658	3.16E-308	0.0228786	0.076618871
TNFRSF10C	rs117020629	7.69E-301	0.923567974	3.70E-302	0.044393848	0.032038178	3.16E-308	0.0197989	0.076432026
CDHR5	rs559896102	3.26E-302	0.923627652	2.36E-303	0.066877713	0.009494634	3.16E-308	0.0228786	0.076372347
RNASE1	rs17254387	1.37E-156	0.923769387	1.08E-157	0.072857094	0.003373519	1.05E-163	0.436277	0.076230613
AXL	rs338583	1.09E-111	0.923824439	8.40E-113	0.071401754	0.004773807	2.00E-118	0.226352	0.076175561
FBLN2	rs527495685	5.39E-231	0.923839954	2.82E-232	0.048216648	0.027943398	2.44E-252	0.214941	0.076160046
HLA-E	rs568422886	3.56E-302	0.923883988	2.54E-303	0.065990291	0.010125721	3.16E-308	0.106809	0.076116012

IL1RAP	rs147147940	1.51E-302	0.924002459	1.07E-303	0.065058635	0.010938906	3.16E-308	0.0308951	0.075997541
CDHR5	rs138630099	3.26E-302	0.924035188	2.35E-303	0.06646578	0.009499032	3.16E-308	0.0228786	0.075964812
TCN2	rs11704165	1.31E-302	0.924100366	7.72E-304	0.054248806	0.021650828	3.16E-308	0.00171945	0.075899634
C7orf50	rs554133434	2.98E-302	0.924199411	2.17E-303	0.067312606	0.008487982	3.16E-308	0.0549794	0.075800588
CEMIP2	rs558370446	5.56E-21	0.92420718	2.78E-22	0.046128287	0.029664533	6.37E-32	0.164668	0.07579282
PTPRH	rs573856022	8.17E-302	0.924296996	4.24E-303	0.047942336	0.027760668	3.16E-308	0.0052342	0.075703004
AXL	rs11083613	1.09E-111	0.924374999	8.33E-113	0.070848348	0.004776652	2.00E-118	0.226352	0.075625
PSAPL1	rs73208486	2.95E-302	0.924438557	2.15E-303	0.067481727	0.008079717	3.16E-308	0.00966207	0.075561444
PTPRH	rs79398811	8.17E-302	0.924451057	4.23E-303	0.047783647	0.027765295	3.16E-308	0.0052342	0.075548942
SERPINE2	rs10184087	1.15E-301	0.92449145	8.62E-303	0.069348948	0.006159602	3.16E-308	0.0568696	0.07550855
TCN2	rs569031504	1.31E-302	0.924502269	7.66E-304	0.053837487	0.021660244	3.16E-308	0.00171945	0.075497731
PTPRH	rs144234283	8.17E-302	0.924581825	4.21E-303	0.047648953	0.027769223	3.16E-308	0.0052342	0.075418176
AXL	rs66841352	1.09E-111	0.924664022	8.30E-113	0.070557832	0.004778146	2.00E-118	0.226352	0.075335978
TCN2	rs561079211	1.95E-302	0.924743233	1.01E-303	0.048077076	0.027179691	3.16E-308	0.00171945	0.075256767
PSAPL1	rs79527344	2.95E-302	0.924807891	2.14E-303	0.067109164	0.008082945	3.16E-308	0.00966207	0.075192109
PSAPL1	rs556939603	2.95E-302	0.924813596	2.14E-303	0.06710341	0.008082994	3.16E-308	0.00966207	0.075186404
FBLN2	rs7623097	5.40E-231	0.924945567	2.75E-232	0.047077593	0.02797684	2.44E-252	0.214941	0.075054433

TACSTD2	rs61779230	1.51E-154	0.925139033	1.18E-155	0.072286103	0.002574864	1.06E-161	0.653489	0.074860967
PSAPL1	rs74491793	2.95E-302	0.92539626	2.12E-303	0.066515653	0.008088087	3.16E-308	0.00966207	0.07460374
FBLN2	rs189700289	8.07E-134	0.925470076	3.45E-135	0.039509394	0.03502053	2.35E-149	0.157699	0.074529924
HLA-E	rs144809585	3.45E-302	0.925872847	2.39E-303	0.064021715	0.010105439	3.16E-308	0.106809	0.074127154
SBSN	rs79170781	4.80E-302	0.92587898	3.68E-303	0.070917286	0.003203734	3.16E-308	0.0992042	0.07412102
LGALS3BP	rs182610403	7.65E-57	0.92595521	4.83E-58	0.058470899	0.015573891	2.45E-64	0.435602	0.07404479
FBLN2	rs2655226	5.41E-231	0.925969973	2.69E-232	0.046022202	0.028007825	2.44E-252	0.214941	0.074030027
TCN2	rs571192363	6.89E-299	0.925987166	4.84E-300	0.065097573	0.008915262	1.86E-306	0.110435	0.074012835
LGALS3BP	rs60653700	7.65E-57	0.926063082	4.82E-58	0.058361213	0.015575705	2.45E-64	0.435602	0.073936918
LGALS3BP	rs111526614	7.65E-57	0.926297862	4.80E-58	0.058122484	0.015579654	2.45E-64	0.435602	0.073702138
TACSTD2	rs187318642	1.51E-302	0.926334324	9.71E-304	0.059535731	0.014129945	3.16E-308	0.0328466	0.073665676
TACSTD2	rs80000309	1.25E-302	0.926464244	7.66E-304	0.056927428	0.016608328	3.16E-308	0.0328466	0.073535756
LGALS3BP	rs12450407	7.65E-57	0.926676808	4.77E-58	0.057737165	0.015586027	2.45E-64	0.435602	0.073323192
SBSN	rs2733743	4.80E-302	0.926719222	3.63E-303	0.070074136	0.003206642	3.16E-308	0.0992042	0.073280778
SBSN	rs117619676	4.80E-302	0.926774301	3.63E-303	0.070018867	0.003206832	3.16E-308	0.0992042	0.073225699
CCN3	rs1601394	4.79E-66	0.926793931	3.60E-67	0.069576952	0.003629117	7.58E-73	0.582488	0.073206069
SBSN	rs73043062	4.81E-302	0.926817448	3.63E-303	0.069975571	0.003206982	3.16E-308	0.0992042	0.073182553

TCN2	rs146572352	1.82E-118	0.926891218	1.26E-119	0.064330945	0.008777837	8.63E-126	0.670128	0.073108782
PSAPL1	rs12647103	2.95E-302	0.926996483	2.07E-303	0.064901443	0.008102073	3.16E-308	0.00966207	0.073003516
LGALS3BP	rs145516713	7.65E-57	0.927009643	4.74E-58	0.057398732	0.015591625	2.45E-64	0.435602	0.072990357
SBSN	rs4609973	4.81E-302	0.92726802	3.60E-303	0.069523439	0.003208541	3.16E-308	0.0992042	0.07273198
CDHR5	rs144867013	3.78E-270	0.927339913	2.59E-271	0.063534298	0.009125789	9.23E-278	0.13878	0.072660087
PBLD	rs116480797	6.76E-303	0.92765633	4.88E-304	0.066907797	0.005435873	3.16E-308	0.00918015	0.07234367
SERPINE2	rs62185104	6.11E-84	0.927732362	4.50E-85	0.06826748	0.004000159	7.52E-91	0.633973	0.072267639
HLA-E	rs114055010	3.56E-302	0.92801902	2.38E-303	0.062096976	0.009884004	3.16E-308	0.143719	0.07198098
RNASE1	rs7157189	1.38E-156	0.928036037	1.02E-157	0.068574863	0.003389101	1.05E-163	0.436277	0.071963964
SMOC2	rs536467303	3.26E-302	0.928209423	2.16E-303	0.061510488	0.010280089	3.16E-308	0.0393251	0.071790577
SLA2	rs114514960	4.18E-18	0.928248288	1.03E-19	0.022878629	0.048873084	1.89E-25	0.418975	0.071751713
TACSTD2	rs143581506	1.04E-302	0.928539751	6.09E-304	0.054234896	0.017225353	3.16E-308	0.031209	0.071460249
SMOC2	rs141581435	2.95E-302	0.928551423	1.95E-303	0.061392012	0.010056565	3.16E-308	0.0393251	0.071448577
SOD3	rs529059785	3.30E-99	0.92864218	1.85E-100	0.052072977	0.019284843	2.82E-111	0.455424	0.07135782
CCN1	rs71502514	2.63E-39	0.928755712	1.80E-40	0.063348343	0.007895945	9.88E-46	0.134575	0.071244288
FLT4	rs539677629	2.99E-09	0.928875515	2.03E-10	0.063024075	0.008100408	2.01E-15	0.28239	0.071124483
SMOC2	rs189620209	2.96E-302	0.929016778	1.94E-303	0.060921617	0.010061605	3.16E-308	0.0393251	0.070983222

FCN2	rs147539232	2.79E-302	0.929322798	1.95E-303	0.064815965	0.005861237	3.16E-308	0.0397027	0.070677202
TCN2	rs117838589	1.37E-302	0.929378543	7.14E-304	0.048228999	0.022392458	3.16E-308	0.00171945	0.070621457
NME3	rs184866855	3.37E-27	0.929513958	1.77E-28	0.048910319	0.021575723	2.76E-34	0.471119	0.070486042
CA9	rs183985235	4.63E-52	0.929596589	2.06E-53	0.041305184	0.029098228	2.53E-67	0.75127	0.070403412
FCN2	rs547523372	2.79E-302	0.929635697	1.94E-303	0.064501092	0.005863211	3.16E-308	0.0397027	0.070364303
FCN2	rs142943184	2.79E-302	0.929661746	1.94E-303	0.064474879	0.005863375	3.16E-308	0.0397027	0.070338254
FCN2	rs141342665	2.79E-302	0.92966377	1.94E-303	0.064472842	0.005863388	3.16E-308	0.0397027	0.07033623
FCN2	rs546940984	2.79E-302	0.929711031	1.94E-303	0.064425283	0.005863686	3.16E-308	0.0397027	0.070288969
FCN2	rs145168059	2.80E-302	0.92992302	1.93E-303	0.064211957	0.005865023	3.16E-308	0.0397027	0.07007698
TCN2	rs5753387	9.01E-302	0.929978585	4.53E-303	0.046781544	0.02323987	3.16E-308	0.029136	0.070021414
FCN2	rs113909851	2.80E-302	0.930011038	1.93E-303	0.064123384	0.005865578	3.16E-308	0.0397027	0.069988962
PRSS8	rs564106654	1.75E-15	0.930046233	6.96E-17	0.036930219	0.033023548	1.18E-23	0.99528	0.069953767
TACSTD2	rs72674184	7.57E-303	0.930065132	4.36E-304	0.053484711	0.016450158	3.16E-308	0.031209	0.069934869
TACSTD2	rs150578624	7.57E-303	0.930132915	4.35E-304	0.053415728	0.016451357	3.16E-308	0.031209	0.069867085
TACSTD2	rs12082192	7.59E-303	0.930333666	4.34E-304	0.053185979	0.016480355	3.16E-308	0.031209	0.069666334
CA9	rs10441686	4.64E-52	0.930430424	2.02E-53	0.040445247	0.029124328	2.53E-67	0.75127	0.069569575
SMOC2	rs11757441	2.96E-302	0.93046292	1.89E-303	0.059459813	0.010077267	3.16E-308	0.0393251	0.06953708

HLA-E	rs114563151	1.84E-302	0.930534347	1.22E-303	0.061405263	0.00806039	3.16E-308	0.143719	0.069465653
CLEC11A	rs34734775	1.02E-275	0.930562395	6.18E-277	0.056374936	0.013062669	2.92E-286	0.250365	0.069437605
LGALS3BP	rs573988072	7.68E-57	0.930645519	4.44E-58	0.053701702	0.015652778	2.45E-64	0.435602	0.06935448
CLEC11A	rs182722517	1.02E-275	0.930765714	6.16E-277	0.056168763	0.013065523	2.92E-286	0.250365	0.069234286
COL5A1	rs12002679	4.33E-161	0.930866535	3.07E-162	0.06609497	0.003038495	2.79E-168	0.478669	0.069133465
HLA-E	rs141031092	2.01E-302	0.93113358	1.31E-303	0.06056994	0.00829648	3.16E-308	0.143719	0.06886642
FBLN2	rs185938007	5.44E-231	0.931170156	2.38E-232	0.040664729	0.028165115	2.44E-252	0.214941	0.068829844
NECTIN4	rs139121685	2.30E-16	0.931419374	8.27E-18	0.033399625	0.035181001	7.64E-23	0.232705	0.068580626
DEFB104B	rs537488634	5.02E-25	0.931682861	2.76E-26	0.051246578	0.017070561	1.71E-31	0.282379	0.068317139
FCN2	rs547702337	2.80E-302	0.931948366	1.87E-303	0.062173837	0.005877797	3.16E-308	0.0397027	0.068051634
ADGRD1	rs79551144	1.90E-300	0.932017716	1.20E-301	0.058718575	0.00926371	3.16E-308	0.0674435	0.067982285
FCN2	rs11103545	2.80E-302	0.932092361	1.86E-303	0.062028934	0.005878705	3.16E-308	0.0397027	0.067907639
FCN2	rs183486037	2.80E-302	0.932139774	1.86E-303	0.061981223	0.005879004	3.16E-308	0.0397027	0.067860227
TCN2	rs193136448	2.02E-301	0.932158178	1.03E-302	0.047522773	0.020319049	3.16E-308	0.0402383	0.067841822
SERPINE2	rs72960461	1.08E-26	0.932596923	6.27E-28	0.054219004	0.013184073	1.68E-33	0.984755	0.067403077
HS1BP3	rs72782390	1.15E-66	0.932637998	6.32E-68	0.051071633	0.016290369	6.52E-77	0.335512	0.067362002
ADGRD1	rs139456901	1.90E-300	0.932647436	1.18E-301	0.058082595	0.009269969	3.16E-308	0.0674435	0.067352564

ADGRD1	rs530757116	1.90E-300	0.932647436	1.18E-301	0.058082595	0.009269969	3.16E-308	0.0674435	0.067352564
CLEC11A	rs141369989	1.02E-275	0.932767754	5.94E-277	0.05413862	0.013093627	2.92E-286	0.250365	0.067232247
CA9	rs7040391	4.65E-52	0.932814234	1.89E-53	0.03798682	0.029198946	2.53E-67	0.75127	0.067185766
ADGRD1	rs10773846	1.90E-300	0.93284278	1.18E-301	0.05788531	0.00927191	3.16E-308	0.0674435	0.06715722
ADAMTS15	rs73044885	2.27E-22	0.933061874	1.50E-23	0.061594346	0.00534378	9.43E-29	0.7643	0.066938126
ACY1	rs549023283	1.66E-193	0.93314758	8.68E-195	0.048699142	0.018153278	8.93E-218	0.794501	0.06685242
PTPRH	rs141964302	9.93E-302	0.933360508	4.97E-303	0.046755334	0.019884158	3.16E-308	0.0137448	0.066639492
SMPD1	rs77690773	4.23E-301	0.933503888	2.90E-302	0.063958778	0.002537334	3.16E-308	0.753744	0.066496112
SMPD1	rs555882632	4.23E-301	0.933522341	2.90E-302	0.063940275	0.002537385	3.16E-308	0.753744	0.06647766
SMOC2	rs143484111	2.97E-302	0.933666816	1.79E-303	0.056221217	0.010111967	3.16E-308	0.0393251	0.066333184
ADAMTS15	rs116897071	2.27E-22	0.933707026	1.48E-23	0.060945499	0.005347475	9.43E-29	0.7643	0.066292974
HS1BP3	rs77551133	1.15E-66	0.933713506	6.18E-68	0.049977339	0.016309155	6.52E-77	0.335512	0.066286494
CDHR5	rs182466501	1.17E-112	0.933782871	6.34E-114	0.050474876	0.015742252	1.04E-122	0.752903	0.066217128
FCN2	rs139679881	2.81E-302	0.933855866	1.81E-303	0.060254307	0.005889827	3.16E-308	0.0397027	0.066144134
CSF1R	rs2569204	4.20E-241	0.933881615	1.68E-242	0.037262928	0.028855457	2.79E-262	0.463089	0.066118385
IL1RAP	rs562085408	2.50E-40	0.933922864	1.36E-41	0.050873041	0.015204096	2.92E-48	0.454389	0.066077137
ACY1	rs533271384	7.96E-61	0.933936439	4.20E-62	0.049241534	0.016822027	2.56E-68	0.478474	0.066063561

CSF1R	rs10079250	4.20E-241	0.933950175	1.67E-242	0.03719225	0.028857575	2.79E-262	0.463089	0.066049825
SOD3	rs146222638	8.29E-25	0.933978614	4.30E-26	0.04843917	0.017582216	3.33E-32	0.607949	0.066021386
CSF1R	rs13178760	4.20E-241	0.934006834	1.67E-242	0.03713384	0.028859326	2.79E-262	0.463089	0.065993166
ADGRD1	rs1195895	1.90E-300	0.934043149	1.15E-301	0.056673009	0.009283841	3.16E-308	0.0674435	0.06595685
CSF1R	rs55721216	4.20E-241	0.934072667	1.67E-242	0.037065973	0.02886136	2.79E-262	0.463089	0.065927333
CTSS	rs114420409	1.17E-256	0.93419997	6.04E-258	0.048305783	0.017494248	5.21E-265	0.0886605	0.065800031
PBLD	rs150546107	6.81E-303	0.934231227	4.40E-304	0.060294373	0.0054744	3.16E-308	0.00918015	0.065768773
SCARA5	rs189091656	2.13E-38	0.934235422	1.24E-39	0.054480972	0.011283606	1.68E-44	0.120313	0.065764578
ACY1	rs121912698	1.66E-193	0.934321185	8.46E-195	0.047502706	0.018176109	8.93E-218	0.794501	0.065678815
ACY1	rs121912701	1.66E-193	0.934352072	8.46E-195	0.047471218	0.01817671	8.93E-218	0.794501	0.065647928
TCN2	rs181903593	2.63E-112	0.934456873	1.56E-113	0.055171091	0.010372035	2.44E-119	0.107352	0.065543126
CSF1R	rs56048668	4.20E-241	0.934511218	1.65E-242	0.036613871	0.028874911	2.79E-262	0.463089	0.065488782
CRYZL1	rs140811901	7.76E-23	0.934777235	3.45E-24	0.041480386	0.023742379	3.16E-29	0.08686	0.065222765
SPOCK1	rs558632389	1.71E-23	0.934900132	6.33E-25	0.03452162	0.030578247	4.05E-30	0.22097	0.065099867
CAMKK1	rs79414225	1.48E-264	0.934906974	8.70E-266	0.054937799	0.010155227	4.68E-272	0.0892833	0.065093026
CAMKK1	rs138204273	1.48E-264	0.934912634	8.70E-266	0.054932077	0.010155289	4.68E-272	0.0892833	0.065087366
NCAM1	rs57250383	1.22E-191	0.934943632	8.21E-193	0.062678693	0.002377675	6.67E-198	0.763029	0.065056368

ADGRD1	rs117117232	1.90E-300	0.935044002	1.13E-301	0.055662209	0.009293789	3.16E-308	0.0674435	0.064955998
ULBP2	rs11753644	5.80E-53	0.935060106	3.35E-54	0.053970669	0.010969224	1.44E-61	0.346584	0.064939893
SUOX	rs186410694	2.64E-06	0.93519581	1.51E-07	0.053649088	0.011152311	2.21E-12	0.0771045	0.064801399
NTRK2	rs10746752	8.18E-14	0.935209863	5.34E-15	0.061091036	0.0036991	4.73E-20	0.527831	0.064790136
CAMKK1	rs753513	1.48E-264	0.935260338	8.65E-266	0.054580596	0.010159066	4.68E-272	0.0892833	0.064739662
CAMKK1	rs181339120	1.48E-264	0.935260338	8.65E-266	0.054580596	0.010159066	4.68E-272	0.0892833	0.064739662
AAMDC	rs113379883	2.88E-298	0.93539469	1.75E-299	0.056698118	0.007907192	3.16E-308	0.73853	0.06460531
SMOC2	rs578140460	2.98E-302	0.935405098	1.73E-303	0.054464109	0.010130793	3.16E-308	0.0393251	0.064594902
SMOC2	rs149250393	2.98E-302	0.935415055	1.73E-303	0.054454044	0.010130901	3.16E-308	0.0393251	0.064584945
SMOC2	rs59291571	2.98E-302	0.935419594	1.73E-303	0.054449456	0.01013095	3.16E-308	0.0393251	0.064580406
CA9	rs10972590	4.66E-52	0.935720295	1.75E-53	0.034989793	0.029289912	2.53E-67	0.75127	0.064279705
SPOCK1	rs116465136	1.71E-23	0.935775197	6.16E-25	0.033617935	0.030606868	4.05E-30	0.22097	0.064224803
PBLD	rs540432535	7.69E-303	0.935893783	4.90E-304	0.05967265	0.004433567	3.16E-308	0.0698072	0.064106217
SMOC2	rs6937356	2.98E-302	0.936002743	1.71E-303	0.053859992	0.010137266	3.16E-308	0.0393251	0.063997258
SMOC2	rs2342545	2.98E-302	0.936138521	1.71E-303	0.053722743	0.010138736	3.16E-308	0.0393251	0.063861479
HBZ	rs557816382	3.22E-302	0.936402022	1.97E-303	0.057420941	0.006177037	3.16E-308	0.00766885	0.063597978
NTRK2	rs12005792	2.25E-19	0.936410572	1.18E-20	0.049094943	0.014494485	1.97E-26	0.673682	0.063589428

F3	rs841692	1.46E-58	0.936718897	9.51E-60	0.061027336	0.002253768	3.72E-65	0.940554	0.063281104
FCN2	rs142313124	2.82E-302	0.936828884	1.72E-303	0.057262538	0.005908578	3.16E-308	0.0397027	0.063171116
SMPD1	rs1050228	4.24E-301	0.936836356	2.75E-302	0.060617252	0.002546392	3.16E-308	0.753744	0.063163644
SMPD1	rs117210486	4.24E-301	0.936838935	2.75E-302	0.060614665	0.002546399	3.16E-308	0.753744	0.063161064
CCN1	rs4949896	2.66E-39	0.93691467	1.56E-40	0.05512002	0.007965309	9.88E-46	0.134575	0.063085329
MASP1	rs145427249	1.37E-163	0.936988588	8.69E-165	0.059396958	0.003614454	8.87E-171	0.364575	0.063011412
SCRN1	rs76710064	2.98E-40	0.937034793	1.59E-41	0.049806439	0.013158768	7.37E-47	0.255981	0.062965207
HS1BP3	rs115108428	1.16E-66	0.93705743	5.76E-68	0.046575007	0.016367563	6.52E-77	0.335512	0.06294257
RELT	rs140221013	1.46E-302	0.937068978	6.63E-304	0.04247099	0.020460032	3.16E-308	0.0039965	0.062931022
COL5A1	rs138033448	4.36E-161	0.937221295	2.78E-162	0.059719468	0.003059238	2.79E-168	0.478669	0.062778706
PBLD	rs74769472	7.16E-303	0.937299945	4.37E-304	0.057178833	0.005521222	3.16E-308	0.00918015	0.062700055
PBLD	rs183570467	1.20E-302	0.937307225	7.52E-304	0.058879507	0.003813268	3.16E-308	0.0842306	0.062692775
CNDP1	rs1149883	1.12E-300	0.9373205	7.16E-302	0.060078959	0.002600542	3.16E-308	0.65974	0.062679501
MASP1	rs35224605	1.37E-163	0.937394123	8.63E-165	0.058989858	0.003616019	8.87E-171	0.364575	0.062605877
NTRK2	rs77562929	2.25E-19	0.937503954	1.15E-20	0.047984658	0.014511388	1.97E-26	0.673682	0.062496046
SMPD1	rs527749153	4.25E-301	0.937714604	2.71E-302	0.059736617	0.002548779	3.16E-308	0.753744	0.062285396
AKR1B1	rs782545	3.50E-61	0.937964455	1.89E-62	0.050702339	0.011333206	3.05E-69	0.613667	0.062035545

SCRN1	rs143640655	2.99E-40	0.937988341	1.56E-41	0.048839501	0.013172159	7.37E-47	0.255981	0.06201166
HBZ	rs184465511	1.44E-302	0.938086371	8.58E-304	0.055744922	0.006168707	3.16E-308	0.00766885	0.061913629
FCRLB	rs61809569	1.24E-300	0.938722979	7.60E-302	0.057372449	0.003904572	3.16E-308	0.705053	0.061277021
CD40	rs117191103	1.23E-89	0.938747629	5.75E-91	0.044047539	0.017204833	1.41E-96	0.0443466	0.061252372
PBLD	rs184930153	9.47E-302	0.938756946	5.66E-303	0.056141279	0.005101775	3.16E-308	0.243068	0.061243054
SUMF2	rs190415510	6.28E-296	0.938873195	2.63E-297	0.039313025	0.02181378	3.16E-308	0.199848	0.061126805
CAPS	rs138201599	6.89E-46	0.938934059	4.15E-47	0.05653309	0.004532851	1.52E-52	0.598175	0.061065941
THBD	rs1042579	3.91E-100	0.93900344	1.75E-101	0.042002847	0.018993713	4.30E-107	0.0442853	0.06099656
FCRLB	rs573476077	1.24E-300	0.939122792	7.55E-302	0.056969779	0.003907429	3.16E-308	0.705053	0.060877208
TNFRSF10C	rs540178390	1.61E-128	0.939141263	8.46E-130	0.049391386	0.011467351	4.68E-142	0.751467	0.060858737
HBZ	rs560304049	1.26E-302	0.9394799	7.24E-304	0.05412032	0.00639978	3.16E-308	0.00766885	0.0605201
FLT4	rs533220024	1.29E-300	0.939518183	7.37E-302	0.053861351	0.006620466	3.16E-308	0.157559	0.060481817
ASGR2	rs8071851	3.02E-301	0.939534812	1.51E-302	0.046840505	0.013624683	3.16E-308	0.0521495	0.060465188
ASGR2	rs540759413	3.02E-301	0.939671126	1.50E-302	0.046702215	0.013626659	3.16E-308	0.0521495	0.060328874
PBLD	rs138250735	3.01E-85	0.939720163	1.85E-86	0.057811599	0.002468238	3.68E-92	0.71856	0.060279837
MASP1	rs17040	1.38E-163	0.939833337	8.27E-165	0.056541235	0.003625428	8.87E-171	0.364575	0.060166663
CDHR5	rs139783880	1.58E-161	0.939877272	9.10E-163	0.054296483	0.005826245	1.06E-169	0.89314	0.060122728

HBZ	rs145102818	8.27E-303	0.939932326	4.73E-304	0.053707252	0.006360422	3.16E-308	0.00766885	0.060067674
HBZ	rs150541949	8.43E-303	0.94003886	4.81E-304	0.053614279	0.006346861	3.16E-308	0.00766885	0.05996114
AKR1B1	rs2229542	3.51E-61	0.940155686	1.81E-62	0.048484632	0.011359682	3.05E-69	0.613667	0.059844314
HBZ	rs118170172	8.43E-303	0.940259302	4.79E-304	0.053392349	0.006348349	3.16E-308	0.00766885	0.059740698
MASP1	rs698090	1.38E-163	0.940298515	8.21E-165	0.056074262	0.003627222	8.87E-171	0.364575	0.059701484
C1QA	rs140942977	4.86E-29	0.940308917	2.87E-30	0.055621257	0.004069826	1.59E-35	0.692306	0.059691083
FCRLB	rs2490419	1.25E-300	0.940357596	7.39E-302	0.055729258	0.003913146	3.16E-308	0.705053	0.059642404
HBZ	rs573904835	1.07E-302	0.940442278	6.07E-304	0.053298371	0.006259352	3.16E-308	0.00766885	0.059557723
COL5A1	rs3132304	4.37E-161	0.940619391	2.62E-162	0.056310279	0.003070329	2.79E-168	0.478669	0.059380608
CPOX	rs9836703	1.89E-37	0.940683476	9.06E-39	0.044955997	0.014360527	4.86E-44	0.0677938	0.059316524
HBZ	rs11644631	9.46E-303	0.940781348	5.30E-304	0.052759748	0.006458904	3.16E-308	0.00766885	0.059218652
NCAM1	rs11214489	2.36E-276	0.94081537	1.28E-277	0.051092967	0.008091663	5.66E-284	0.132662	0.05918463
C1QA	rs77962086	1.67E-276	0.940944228	6.75E-278	0.037917468	0.021138304	1.43E-284	0.0881333	0.059055772
HBZ	rs144980572	8.28E-303	0.941011203	4.63E-304	0.052621074	0.006367723	3.16E-308	0.00766885	0.058988797
SCRN1	rs531615379	3.00E-40	0.94105117	1.46E-41	0.04573366	0.01321517	7.37E-47	0.255981	0.05894883
HBZ	rs144007563	8.28E-303	0.941147698	4.62E-304	0.052483656	0.006368646	3.16E-308	0.00766885	0.058852302
ASAH2	rs185349522	1.72E-297	0.941214811	9.20E-299	0.050420275	0.008364915	3.16E-308	0.759007	0.05878519

COL5A1	rs3118555	4.38E-161	0.941405585	2.58E-162	0.055521519	0.003072896	2.79E-168	0.478669	0.058594415
SCRN1	rs12531533	3.00E-40	0.941602597	1.44E-41	0.045174489	0.013222914	7.37E-47	0.255981	0.058397403
CPOX	rs1729988	1.90E-37	0.941603093	8.87E-39	0.044022341	0.014374566	4.86E-44	0.0677938	0.058396907
HBZ	rs9940455	3.91E-302	0.941640364	2.25E-303	0.054197812	0.004161825	3.16E-308	0.0271619	0.058359637
CDON	rs74612335	1.21E-22	0.941813503	7.07E-24	0.05515532	0.003031178	2.61E-28	0.638669	0.058186498
AKR1B1	rs118087724	4.51E-12	0.941832322	2.02E-13	0.042049507	0.016118172	2.07E-18	0.307744	0.058167679
THBD	rs138167689	3.93E-100	0.941919998	1.63E-101	0.039027294	0.019052708	4.30E-107	0.0442853	0.058080002
BMPER	rs187596433	6.97E-107	0.941949166	3.81E-108	0.051465096	0.006585738	5.27E-114	0.306719	0.058050834
CNDP1	rs117190257	1.21E-10	0.941956764	3.82E-12	0.029711329	0.028331907	4.94E-17	0.129945	0.058043236
CD59	rs10836121	7.72E-222	0.941966567	3.23E-223	0.039408444	0.018624988	6.50E-229	0.0409232	0.058033432
NCAM1	rs17115160	2.36E-276	0.942122482	1.25E-277	0.049774613	0.008102905	5.66E-284	0.132662	0.057877518
FN1	rs188014784	2.01E-82	0.942227201	1.14E-83	0.053635518	0.004137281	2.65E-89	0.352952	0.057772799
CD59	rs4756046	7.72E-222	0.942302795	3.20E-223	0.039065569	0.018631636	6.50E-229	0.0409232	0.057697205
LY75	rs72947569	6.52E-303	0.942373007	3.50E-304	0.050610458	0.007016535	3.16E-308	0.0196006	0.057626993
AAMDC	rs181353854	6.76E-302	0.942378888	3.78E-303	0.052756947	0.004864165	3.16E-308	0.110683	0.057621112
LY75	rs532749810	6.35E-303	0.942446422	3.41E-304	0.050620058	0.006933519	3.16E-308	0.0196006	0.057553577
NCAM1	rs150818668	2.36E-276	0.942446878	1.24E-277	0.049447427	0.008105695	5.66E-284	0.132662	0.057553122

CD59	rs704701	7.73E-222	0.942520366	3.19E-223	0.038843696	0.018635938	6.50E-229	0.0409232	0.057479634
SCRN1	rs17324153	3.00E-40	0.94252169	1.41E-41	0.044242489	0.01323582	7.37E-47	0.255981	0.057478309
SCRN1	rs151249549	3.00E-40	0.94252169	1.41E-41	0.044242489	0.01323582	7.37E-47	0.255981	0.057478309
FLT4	rs72816969	1.29E-300	0.942574335	6.95E-302	0.050783663	0.006642001	3.16E-308	0.157559	0.057425664
C1QA	rs576405362	3.54E-302	0.942685359	1.85E-303	0.049203937	0.008110704	3.16E-308	0.0165871	0.057314641
ST6GAL1	rs143709054	8.40E-27	0.942714264	4.01E-28	0.044987795	0.012297942	4.69E-34	0.293081	0.057285737
METAP1D	rs148302929	0.000317661	0.94239462	1.60E-05	0.04753146	0.009740234	9.14E-10	0.903444	0.057271694
PBLD	rs16925142	7.59E-303	0.942779627	4.16E-304	0.051661864	0.005558508	3.16E-308	0.00918015	0.057220372
ASGR2	rs145127644	3.03E-301	0.942919428	1.40E-302	0.043406807	0.013673765	3.16E-308	0.0521495	0.057080572
FCRLB	rs537469177	4.59E-301	0.942929777	2.56E-302	0.0526681	0.004402123	3.16E-308	0.762304	0.057070223
CLMP	rs10790538	7.45E-106	0.942947788	3.80E-107	0.04814504	0.008907173	4.51E-114	0.664232	0.057052213
FCN2	rs186673560	4.81E-300	0.942952235	2.70E-301	0.053021184	0.004026581	3.16E-308	0.967833	0.057047765
ASGR2	rs150983647	3.03E-301	0.942967144	1.40E-302	0.043358399	0.013674457	3.16E-308	0.0521495	0.057032856
CNDP1	rs147132449	1.12E-300	0.942997311	6.48E-302	0.054386397	0.002616292	3.16E-308	0.65974	0.057002689
FCRLB	rs553630704	4.70E-301	0.943045411	2.63E-302	0.052642855	0.004311734	3.16E-308	0.762304	0.056954589
WASF1	rs1980532	1.21E-301	0.943048767	6.11E-303	0.047761819	0.009189414	3.16E-308	0.0859409	0.056951233
ASGR2	rs2304980	3.03E-301	0.943115396	1.39E-302	0.043207997	0.013676607	3.16E-308	0.0521495	0.056884604

LY75	rs192961413	4.85E-303	0.943142107	2.60E-304	0.050598428	0.006259465	3.16E-308	0.0196006	0.056857893
LY75	rs189150429	4.88E-303	0.943224393	2.61E-304	0.050504	0.006271607	3.16E-308	0.0196006	0.056775607
HBZ	rs551276029	1.09E-301	0.943332358	6.11E-303	0.052850454	0.003817188	3.16E-308	0.180569	0.056667642
TACSTD2	rs72680103	3.31E-144	0.943451774	1.72E-145	0.049055109	0.007493116	2.40E-151	0.138893	0.056548225
EDN1	rs5370	1.13E-190	0.943692124	6.28E-192	0.052512034	0.003795842	5.35E-198	0.456542	0.056307876
HBZ	rs185296806	9.25E-303	0.943853756	4.87E-304	0.049735986	0.006410258	3.16E-308	0.00766885	0.056146244
HBZ	rs568736001	9.36E-303	0.943907164	4.92E-304	0.049650575	0.006442262	3.16E-308	0.00766885	0.056092837
NCAM1	rs118003718	2.37E-276	0.944085135	1.20E-277	0.04779508	0.008119785	5.66E-284	0.132662	0.055914865
HBZ	rs181309991	9.41E-303	0.944111487	4.93E-304	0.049429601	0.006458913	3.16E-308	0.00766885	0.055888514
ALDH3A1	rs79885209	7.84E-105	0.944214243	3.92E-106	0.047234665	0.008551092	3.16E-111	0.108483	0.055785757
LEPR	rs10399687	7.98E-167	0.944549319	3.84E-168	0.045453977	0.009996704	1.66E-173	0.143945	0.055450681
HBZ	rs145355462	8.31E-303	0.944686865	4.30E-304	0.048920515	0.006392621	3.16E-308	0.00766885	0.055313136
ALDH3A1	rs576583123	7.85E-105	0.944777271	3.88E-106	0.046666538	0.008556191	3.16E-111	0.108483	0.055222729
OXT	rs6139014	5.82E-302	0.94484126	3.20E-303	0.051985396	0.003173343	3.16E-308	0.155594	0.055158739
CSF3R	rs191631784	5.81E-74	0.944875323	2.90E-75	0.047194562	0.007930115	8.03E-81	0.129777	0.055124677
LY75	rs60343914	4.77E-303	0.944880154	2.47E-304	0.048885988	0.006233858	3.16E-308	0.0196006	0.055119846
AGRP	rs28765128	6.12E-18	0.94488661	2.28E-19	0.035112194	0.020001196	1.06E-24	0.92843	0.05511339

PROK1	rs190851423	3.73E-68	0.944895403	1.80E-69	0.045667219	0.009437378	1.92E-75	0.388466	0.055104597
WASF1	rs56197201	1.21E-301	0.945011978	5.86E-303	0.045779477	0.009208545	3.16E-308	0.0859409	0.054988022
OXT	rs6051570	5.82E-302	0.945204519	3.18E-303	0.051620918	0.003174563	3.16E-308	0.155594	0.054795481
ULBP2	rs567926948	1.85E-302	0.945261264	1.00E-303	0.051279963	0.003458773	3.16E-308	0.779866	0.054738736
AGRP	rs35714475	6.13E-18	0.945313808	2.25E-19	0.034676235	0.020009957	1.06E-24	0.92843	0.054686192
OXT	rs59478431	5.82E-302	0.945442988	3.17E-303	0.051381647	0.003175364	3.16E-308	0.155594	0.054557011
CEMIP2	rs147032151	1.10E-15	0.945564409	3.66E-17	0.03155902	0.022876571	4.34E-24	0.243158	0.054435591
HBZ	rs147346513	9.05E-303	0.945701477	4.59E-304	0.047955146	0.006343377	3.16E-308	0.00766885	0.054298523
CLMP	rs11219040	2.35E-302	0.945735809	1.28E-303	0.051258404	0.003005787	3.16E-308	0.2911	0.054264191
FLT4	rs1565818	1.29E-300	0.945749464	6.51E-302	0.04758616	0.006664375	3.16E-308	0.157559	0.054250535
FLT4	rs564100331	1.29E-300	0.945807296	6.51E-302	0.047527921	0.006664783	3.16E-308	0.157559	0.054192704
ULBP2	rs191033750	2.12E-45	0.945808463	1.14E-46	0.050810099	0.003381438	4.66E-52	0.864744	0.054191537
AGRP	rs188707867	6.14E-18	0.945873335	2.21E-19	0.034104864	0.020021801	1.06E-24	0.92843	0.054126665
CNDP1	rs17817077	1.13E-300	0.945913676	6.13E-302	0.051461941	0.002624383	3.16E-308	0.65974	0.054086324
CNDP1	rs78939422	1.13E-300	0.945920387	6.13E-302	0.051455211	0.002624402	3.16E-308	0.65974	0.054079613
LY75	rs189812553	1.98E-303	0.945966797	1.01E-304	0.04841147	0.005621733	3.16E-308	0.0248193	0.054033203
CLMP	rs11822892	2.36E-302	0.946107749	1.27E-303	0.050885282	0.003006969	3.16E-308	0.2911	0.053892251

CLEC4C	rs61742705	1.58E-79	0.946129136	7.20E-81	0.04307794	0.010792924	1.88E-86	0.171847	0.053870864
RNASET2	rs145814949	5.48E-303	0.946247599	2.96E-304	0.051001877	0.002750524	3.16E-308	0.0693538	0.053752401
IFNAR1	rs144986404	5.24E-302	0.946492873	2.62E-303	0.047286026	0.006221101	3.16E-308	0.125534	0.053507127
OXT	rs6107227	5.83E-302	0.946494314	3.10E-303	0.050326791	0.003178895	3.16E-308	0.155594	0.053505686
LY75	rs186640093	1.91E-303	0.9465093	9.68E-305	0.048032265	0.005458435	3.16E-308	0.0358443	0.0534907
CLMP	rs4435015	2.36E-302	0.946513365	1.26E-303	0.050478377	0.003008258	3.16E-308	0.2911	0.053486635
IFNAR1	rs62654645	5.24E-302	0.946533502	2.62E-303	0.047245131	0.006221368	3.16E-308	0.125534	0.053466499
FCRLB	rs569527056	9.42E-302	0.94659315	4.88E-303	0.048992418	0.004414432	3.16E-308	0.122387	0.05340685
COMT	rs117246610	7.03E-302	0.946604657	3.54E-303	0.04763724	0.005758103	3.16E-308	0.0698747	0.053395343
AAMDC	rs571879396	1.86E-302	0.94660488	9.59E-304	0.048703334	0.004691787	3.16E-308	0.110683	0.053395121
ADAMTS15	rs3794132	2.39E-17	0.946613902	1.28E-18	0.050489023	0.002897075	1.66E-22	0.54137	0.053386098
LY75	rs111340541	1.90E-303	0.94663265	9.64E-305	0.047912659	0.005454691	3.16E-308	0.0358443	0.05336735
ULBP2	rs537369219	1.85E-302	0.946683269	9.76E-304	0.049852755	0.003463976	3.16E-308	0.779866	0.053316731
LY75	rs146328612	1.92E-303	0.946683464	9.70E-305	0.047871827	0.005444709	3.16E-308	0.0358443	0.053316536
ULBP2	rs10457857	1.85E-302	0.946706169	9.75E-304	0.049829771	0.00346406	3.16E-308	0.779866	0.053293831
LY75	rs13407818	1.91E-303	0.946730076	9.64E-305	0.047823491	0.005446433	3.16E-308	0.0358443	0.053269924
LY75	rs140690255	1.90E-303	0.946782879	9.59E-305	0.047802144	0.005414977	3.16E-308	0.0358443	0.053217121

LY75	rs75345955	1.90E-303	0.946800239	9.60E-305	0.047787318	0.005412443	3.16E-308	0.0358443	0.053199761
AAMDC	rs71469599	1.23E-302	0.946855384	6.22E-304	0.047977356	0.005167259	3.16E-308	0.166555	0.053144615
LY75	rs558056881	2.38E-303	0.947038711	1.18E-304	0.047102784	0.005858505	3.16E-308	0.0248193	0.052961289
TNFRSF10C	rs573892334	2.32E-162	0.947040806	1.16E-163	0.04711509	0.005844104	1.43E-169	0.184673	0.052959194
IL32	rs550008175	1.68E-08	0.947061464	6.61E-10	0.037177986	0.015760532	1.32E-14	0.382671	0.052938518
ULBP2	rs78912369	1.85E-302	0.947093195	9.68E-304	0.049441329	0.003465476	3.16E-308	0.779866	0.052906805
LY75	rs539859475	1.94E-303	0.94719512	9.70E-305	0.047468341	0.005336539	3.16E-308	0.0358443	0.05280488
ORM1	rs1408524	3.42E-300	0.947269586	1.56E-301	0.043150064	0.00958035	3.16E-308	0.356768	0.052730414
CLMP	rs530402446	2.36E-302	0.947285421	1.24E-303	0.049703867	0.003010712	3.16E-308	0.2911	0.052714579
LY75	rs72954858	1.92E-303	0.947317654	9.62E-305	0.047341365	0.005340981	3.16E-308	0.0358443	0.052682346
LY75	rs193264414	2.10E-303	0.947359742	1.05E-304	0.047540396	0.005099862	3.16E-308	0.0358443	0.052640258
LY75	rs139914382	2.41E-303	0.947365728	1.19E-304	0.046781114	0.005853158	3.16E-308	0.0248193	0.052634272
LY75	rs78169282	2.06E-303	0.947520063	1.03E-304	0.047330373	0.005149565	3.16E-308	0.0358443	0.052479938
AAMDC	rs61900190	1.29E-302	0.947549989	6.44E-304	0.047322628	0.005127383	3.16E-308	0.166555	0.052450011
EDN1	rs571803515	1.05E-36	0.947591431	5.53E-38	0.049828867	0.002579701	2.20E-43	0.630028	0.052408568
PTPRH	rs564928326	3.49E-301	0.947649494	1.64E-302	0.044392773	0.007957733	3.16E-308	0.0455848	0.052350506
ORM1	rs564000636	3.42E-300	0.94765922	1.55E-301	0.042756489	0.009584291	3.16E-308	0.356768	0.05234078

RNASET2	rs36015563	1.13E-302	0.947671651	5.94E-304	0.049780164	0.002548185	3.16E-308	0.248891	0.052328349
LY75	rs140657634	2.50E-303	0.947733029	1.23E-304	0.046399729	0.005867242	3.16E-308	0.0248193	0.052266971
CNDP1	rs58692747	1.13E-300	0.947738615	5.91E-302	0.049631939	0.002629446	3.16E-308	0.65974	0.052261385
CD40	rs12624433	8.06E-302	0.947743555	4.02E-303	0.04733775	0.004918696	3.16E-308	0.152183	0.052256446
LY75	rs181947337	2.22E-303	0.94784669	1.11E-304	0.047208716	0.004944594	3.16E-308	0.0358443	0.05215331
TRIM5	rs10742818	3.73E-301	0.947861972	1.89E-302	0.048129384	0.004008644	3.16E-308	0.600904	0.052138028
FLT4	rs115838135	1.30E-300	0.947962217	6.21E-302	0.045357815	0.006679968	3.16E-308	0.157559	0.052037783
PDCD1LG2	rs532069557	1.15E-302	0.947974603	5.61E-304	0.046107361	0.005918036	3.16E-308	0.0224864	0.052025397
RELT	rs78973710	2.28E-302	0.947988294	9.36E-304	0.038980061	0.013031646	3.16E-308	0.0115359	0.052011707
FLT4	rs538639730	1.30E-300	0.948063675	6.20E-302	0.045255642	0.006680683	3.16E-308	0.157559	0.051936325
PDCD1LG2	rs193181528	4.99E-51	0.948064198	2.34E-52	0.044443699	0.007492102	3.03E-58	0.829399	0.051935801
WASF1	rs2078890	1.21E-301	0.948082571	5.46E-303	0.042678963	0.009238466	3.16E-308	0.0859409	0.051917429
ORM1	rs149509114	3.43E-300	0.948166241	1.53E-301	0.04224434	0.009589419	3.16E-308	0.356768	0.051833759
EDN1	rs76259907	1.05E-36	0.948239681	5.46E-38	0.049179216	0.002581103	2.20E-43	0.630028	0.051760319
AAMDC	rs556128179	1.17E-302	0.948250687	5.73E-304	0.04655679	0.005192523	3.16E-308	0.166555	0.051749313
ADAMTS16	rs40468	1.55E-54	0.948258683	7.95E-56	0.048735342	0.003005976	5.30E-61	0.53419	0.051741318
GUSB	rs149606212	2.23E-59	0.948380803	6.48E-61	0.027515111	0.024104086	1.25E-76	0.522503	0.051619197

ALCAM	rs183258379	6.36E-73	0.948384447	2.88E-74	0.0429004	0.008715153	5.54E-80	0.396392	0.051615553
LY75	rs114450674	2.24E-303	0.94839596	1.10E-304	0.046710732	0.004893309	3.16E-308	0.0358443	0.051604041
IDO1	rs2729468	1.49E-252	0.948515402	7.38E-254	0.046868529	0.004616069	7.29E-260	0.267346	0.051484598
ULBP2	rs190861751	1.86E-302	0.948538538	9.39E-304	0.047990697	0.003470765	3.16E-308	0.779866	0.051461462
ULBP2	rs183414256	1.86E-302	0.948538538	9.39E-304	0.047990697	0.003470765	3.16E-308	0.779866	0.051461462
IDO1	rs1362846	1.58E-234	0.948583834	7.81E-236	0.04687884	0.004537326	1.26E-241	0.267346	0.051416166
LY75	rs555105506	2.64E-303	0.948595221	1.27E-304	0.045484382	0.005920397	3.16E-308	0.0248193	0.051404779
IFNAR1	rs2834175	5.25E-302	0.948625206	2.50E-303	0.045139678	0.006235116	3.16E-308	0.125534	0.051374794
ULBP2	rs4555924	1.86E-302	0.948654914	9.37E-304	0.047873895	0.003471191	3.16E-308	0.779866	0.051345086
FCRLB	rs139776327	4.08E-302	0.948785952	1.99E-303	0.046249957	0.00496409	3.16E-308	0.125692	0.051214047
ULBP2	rs189016334	1.86E-302	0.948809606	9.34E-304	0.047718637	0.003471757	3.16E-308	0.779866	0.051190394
IDO1	rs1362846	5.32E-244	0.948845767	2.62E-245	0.04678268	0.004371553	2.02E-251	0.294061	0.051154233
IDO1	rs1035280	4.06E-230	0.948901431	1.99E-231	0.046553784	0.004544785	3.23E-237	0.267346	0.051098569
RELT	rs149832240	2.41E-302	0.948982632	9.64E-304	0.0380201	0.012997268	3.16E-308	0.0115359	0.051017368
CNDP1	rs148000502	1.13E-300	0.948999494	5.76E-302	0.048367562	0.002632944	3.16E-308	0.65974	0.051000506
COMT	rs174695	7.05E-302	0.949065506	3.36E-303	0.045161422	0.005773072	3.16E-308	0.0698747	0.050934494
ALCAM	rs7647426	6.37E-73	0.949068198	2.83E-74	0.042210366	0.008721436	5.54E-80	0.396392	0.050931802

ALCAM	rs114487521	6.37E-73	0.949092566	2.83E-74	0.042185774	0.00872166	5.54E-80	0.396392	0.050907434
COMT	rs5993819	1.58E-200	0.94931577	7.91E-202	0.047410726	0.003273504	1.46E-207	0.391288	0.05068423
IDO1	rs7010461	1.49E-252	0.949518423	7.22E-254	0.045860627	0.00462095	7.29E-260	0.267346	0.050481577
FGFBP3	rs182892216	1.09E-301	0.949518762	4.17E-303	0.036410857	0.014070381	3.16E-308	0.0269495	0.050481238
PDCD1LG2	rs12341823	1.16E-302	0.949614437	5.41E-304	0.044457289	0.005928274	3.16E-308	0.0224864	0.050385563
AOC3	rs72831075	8.26E-163	0.949645645	2.63E-164	0.030158063	0.020196291	1.12E-171	0.246762	0.050354354
C1QA	rs12060537	3.56E-302	0.949652844	1.58E-303	0.042176506	0.008170651	3.16E-308	0.0165871	0.050347157
LY75	rs182765113	2.22E-303	0.949695951	1.06E-304	0.045448338	0.004855711	3.16E-308	0.0358443	0.050304049
ALCAM	rs565661354	6.37E-73	0.949725716	2.79E-74	0.041546805	0.008727478	5.54E-80	0.396392	0.050274283
IDO1	rs7846217	1.49E-252	0.94973128	7.19E-254	0.045646734	0.004621986	7.29E-260	0.267346	0.05026872
ALCAM	rs77996868	6.37E-73	0.949753561	2.79E-74	0.041518705	0.008727734	5.54E-80	0.396392	0.050246439
LY75	rs553143511	3.21E-303	0.949803425	1.49E-304	0.044152299	0.006044276	3.16E-308	0.0248193	0.050196575
SHISA5	rs4075082	2.13E-56	0.949851131	6.85E-58	0.030574762	0.019574107	1.44E-63	0.513801	0.050148869
IDO1	rs7820268	1.58E-234	0.949971262	7.57E-236	0.045484776	0.004543962	1.26E-241	0.267346	0.050028738
C1QA	rs35477594	3.56E-302	0.950031183	1.57E-303	0.041794911	0.008173906	3.16E-308	0.0165871	0.049968817
SHISA5	rs186501638	2.13E-56	0.950047184	6.80E-58	0.030374871	0.019577945	1.44E-63	0.513801	0.049952816
IDO1	rs7010461	4.07E-230	0.950063397	1.94E-231	0.045386253	0.00455035	3.23E-237	0.267346	0.049936603

ALCAM	rs34926152	6.38E-73	0.950075024	2.77E-74	0.041194288	0.008730688	5.54E-80	0.396392	0.049924976
IFNAR1	rs187992350	5.26E-302	0.950233318	2.41E-303	0.043520996	0.006245686	3.16E-308	0.125534	0.049766682
IDO1	rs7820268	5.33E-244	0.950233962	2.54E-245	0.04538809	0.004377949	2.02E-251	0.294061	0.049766039
SHISA5	rs114743756	2.13E-56	0.950265059	6.75E-58	0.030152303	0.019582637	1.44E-63	0.513801	0.04973494
IDO1	rs7846217	4.07E-230	0.950276498	1.93E-231	0.045172131	0.004551371	3.23E-237	0.267346	0.049723502
SHISA5	rs11706087	2.13E-56	0.950292088	6.75E-58	0.030124718	0.019583194	1.44E-63	0.513801	0.049707912
SNCG	rs147223437	1.98E-301	0.950332244	9.72E-303	0.046572391	0.003095365	3.16E-308	0.190811	0.049667756
LY75	rs548556986	4.52E-303	0.950365326	2.07E-304	0.043502441	0.006132233	3.16E-308	0.0196006	0.049634674
ALCAM	rs549451872	6.38E-73	0.950403355	2.74E-74	0.040862939	0.008733705	5.54E-80	0.396392	0.049596644
LY75	rs537086678	2.19E-303	0.950483923	1.03E-304	0.044673499	0.004842579	3.16E-308	0.0358443	0.049516078
LY75	rs188480085	2.20E-303	0.950502883	1.03E-304	0.044647123	0.004849994	3.16E-308	0.0358443	0.049497117
HBZ	rs3785309	8.79E-303	0.9505763	3.97E-304	0.042866474	0.006557226	3.16E-308	0.00766885	0.0494237
HBZ	rs150224431	8.79E-303	0.950615917	3.96E-304	0.042826584	0.0065575	3.16E-308	0.00766885	0.049384084
CLEC4C	rs186026650	9.37E-303	0.950670116	4.31E-304	0.043740527	0.005589357	3.16E-308	0.821586	0.049329884
CLEC4C	rs183882761	9.37E-303	0.950673071	4.31E-304	0.043737555	0.005589374	3.16E-308	0.821586	0.049326929
HBZ	rs570634846	8.79E-303	0.950741137	3.95E-304	0.0427005	0.006558363	3.16E-308	0.00766885	0.049258863
AAMDC	rs117804781	8.84E-303	0.950792773	4.07E-304	0.043791446	0.005415781	3.16E-308	0.166555	0.049207227

FLRT2	rs2747005	2.44E-302	0.950805465	1.17E-303	0.045622332	0.003572203	3.16E-308	0.18097	0.049194535
C1QA	rs80352808	3.57E-302	0.950903269	1.53E-303	0.040915322	0.008181409	3.16E-308	0.0165871	0.049096731
CEMIP2	rs117071203	2.05E-66	0.950916869	9.68E-68	0.044866728	0.004216403	9.03E-73	0.7141	0.049083131
LY75	rs186219585	3.35E-303	0.95092774	1.52E-304	0.043095469	0.00597679	3.16E-308	0.0248193	0.049072259
GZMB	rs61976815	8.26E-302	0.950939369	3.76E-303	0.04332486	0.005735771	3.16E-308	0.159244	0.049060631
ULBP2	rs573094432	1.87E-302	0.950950362	8.99E-304	0.045613651	0.003435988	3.16E-308	0.779866	0.049049639
SNCG	rs551714373	1.19E-301	0.950973944	5.80E-303	0.04617494	0.002851116	3.16E-308	0.190811	0.049026056
ADGRD1	rs189568518	3.03E-178	0.951133228	1.45E-179	0.045449205	0.003417567	1.78E-185	0.370662	0.048866772
CD40	rs45456397	3.56E-165	0.951142488	1.54E-166	0.041262867	0.007594645	1.44E-172	0.214691	0.048857512
FLRT2	rs12896539	2.44E-302	0.95116707	1.16E-303	0.045259369	0.003573562	3.16E-308	0.18097	0.048832931
CLEC4C	rs183763502	9.37E-303	0.951169692	4.26E-304	0.043238014	0.005592294	3.16E-308	0.821586	0.048830308
CLEC4C	rs188632604	9.37E-303	0.951182516	4.26E-304	0.043225114	0.005592369	3.16E-308	0.821586	0.048817483
LY75	rs187039299	3.76E-303	0.951211514	1.70E-304	0.042964408	0.005824078	3.16E-308	0.0248193	0.048788486
ORM1	rs548563216	1.81E-166	0.951233289	8.38E-168	0.043951674	0.004815037	5.82E-174	0.830651	0.048766711
FLRT2	rs140128260	1.27E-301	0.951238222	5.94E-303	0.044361147	0.004400631	3.16E-308	0.210972	0.048761778
CLEC4C	rs187207576	9.37E-303	0.951409804	4.24E-304	0.042996491	0.005593705	3.16E-308	0.821586	0.048590196
CLEC4C	rs150032474	9.37E-303	0.951497668	4.23E-304	0.04290811	0.005594222	3.16E-308	0.821586	0.048502332

IFNAR1	rs192304966	5.27E-302	0.951631284	2.33E-303	0.042113841	0.006254875	3.16E-308	0.125534	0.048368716
ASAH2	rs574035798	2.30E-302	0.951646081	9.70E-304	0.040187536	0.008166383	3.16E-308	0.0684573	0.048353919
EPHA2	rs2064595	1.86E-65	0.951720295	8.45E-67	0.04329393	0.004985775	3.12E-72	0.238301	0.048279705
FLRT2	rs115482915	9.36E-302	0.951823562	4.33E-303	0.044001871	0.004174566	3.16E-308	0.210972	0.048176437
PDCD1LG2	rs150600191	1.25E-302	0.951831598	5.73E-304	0.043484889	0.004683513	3.16E-308	0.0409327	0.048168402
CLEC4C	rs182016866	9.38E-303	0.951833827	4.19E-304	0.042569975	0.005596198	3.16E-308	0.821586	0.048166173
OXT	rs2422835	5.86E-302	0.951886135	2.77E-303	0.044916861	0.003197004	3.16E-308	0.155594	0.048113865
CLEC4C	rs12825559	9.38E-303	0.951890331	4.19E-304	0.042513138	0.005596531	3.16E-308	0.821586	0.048109669
CLEC4C	rs189655569	9.38E-303	0.951918535	4.19E-304	0.042484769	0.005596696	3.16E-308	0.821586	0.048081465
EPHA2	rs11543934	1.86E-65	0.951929612	8.41E-67	0.043083517	0.004986871	3.12E-72	0.238301	0.048070388
CLEC4C	rs745925419	9.38E-303	0.951946767	4.18E-304	0.04245637	0.005596862	3.16E-308	0.821586	0.048053232
CLEC4C	rs556507988	9.38E-303	0.951948904	4.18E-304	0.042454221	0.005596875	3.16E-308	0.821586	0.048051096
CLEC4C	rs770603178	9.38E-303	0.951975627	4.18E-304	0.042427341	0.005597032	3.16E-308	0.821586	0.048024373
CLEC4C	rs999075	9.38E-303	0.95198158	4.18E-304	0.042421353	0.005597067	3.16E-308	0.821586	0.04801842
ULBP2	rs557674725	2.00E-302	0.952120764	9.38E-304	0.044630946	0.00324829	3.16E-308	0.779866	0.047879236
EPHA2	rs924204	1.86E-65	0.95216407	8.36E-67	0.04284783	0.0049881	3.12E-72	0.238301	0.04783593
AGT	rs191596362	9.59E-14	0.952170909	4.53E-15	0.044946797	0.002882294	7.67E-19	0.467505	0.047829091

CLEC4C	rs771138675	9.38E-303	0.952195945	4.16E-304	0.042205727	0.005598327	3.16E-308	0.821586	0.047804054
C1QA	rs74414073	3.76E-302	0.95224219	1.59E-303	0.040333935	0.007423875	3.16E-308	0.0197865	0.04775781
FLRT2	rs117677498	3.16E-302	0.952328996	1.46E-303	0.044061452	0.003609552	3.16E-308	0.381586	0.047671004
C1QA	rs150204071	3.57E-302	0.952329382	1.48E-303	0.039476939	0.008193679	3.16E-308	0.0165871	0.047670618
CLEC4C	rs75173186	9.38E-303	0.952334432	4.14E-304	0.042066426	0.005599142	3.16E-308	0.821586	0.047665568
GUSB	rs3112832	2.23E-59	0.952347413	5.52E-61	0.02352792	0.024124667	1.25E-76	0.522503	0.047652587
C1QA	rs537183572	4.05E-302	0.95235728	1.70E-303	0.040083851	0.007558868	3.16E-308	0.0197865	0.047642719
CLEC4C	rs190700056	9.38E-303	0.952384531	4.14E-304	0.042016032	0.005599436	3.16E-308	0.821586	0.047615468
IFNAR1	rs17860183	5.27E-302	0.952466638	2.29E-303	0.041272996	0.006260365	3.16E-308	0.125534	0.047533361
PVALB	rs6000386	5.49E-302	0.95249293	2.47E-303	0.042742121	0.004764949	3.16E-308	0.0760764	0.04750707
HBZ	rs571996052	8.94E-303	0.95250883	3.84E-304	0.040875815	0.006615354	3.16E-308	0.00766885	0.047491169
PDCD1LG2	rs142124284	1.26E-302	0.95254849	5.64E-304	0.04276447	0.00468704	3.16E-308	0.0409327	0.04745151
AAMDC	rs72935507	8.91E-303	0.952614345	3.92E-304	0.041931095	0.00545456	3.16E-308	0.166555	0.047385655
CLEC4C	rs188258470	9.38E-303	0.952618982	4.12E-304	0.041780203	0.005600815	3.16E-308	0.821586	0.047381018
EPHA2	rs147352564	1.86E-65	0.952648217	8.27E-67	0.042361147	0.004990636	3.12E-72	0.238301	0.047351783
LY75	rs183601344	5.08E-303	0.952663714	2.19E-304	0.041050018	0.006286268	3.16E-308	0.0248193	0.047336286
IDO1	rs76640362	1.59E-234	0.952750532	7.11E-236	0.042692212	0.004557256	1.26E-241	0.267346	0.047249468

TRIM5	rs11821656	3.75E-301	0.95280696	1.70E-302	0.043163482	0.004029558	3.16E-308	0.600904	0.04719304
TRIM5	rs1074354	3.75E-301	0.952857692	1.69E-302	0.043112536	0.004029772	3.16E-308	0.600904	0.047142308
HBZ	rs118006258	9.01E-303	0.952918338	3.82E-304	0.040445365	0.006636297	3.16E-308	0.00766885	0.047081662
FGFBP3	rs11186973	2.32E-118	0.952989579	1.03E-119	0.04224672	0.004763701	4.13E-125	0.410348	0.047010421
ANXA2	rs72744887	4.02E-302	0.953011433	1.73E-303	0.040931356	0.006057211	3.16E-308	0.0675632	0.046988567
IDO1	rs76640362	5.34E-244	0.953047792	2.39E-245	0.042561295	0.004390913	2.02E-251	0.294061	0.046952208
PCDH9	rs71446822	6.23E-92	0.953089971	2.86E-93	0.043722095	0.003187935	7.09E-99	0.550739	0.04691003
LEPR	rs1171277	8.05E-167	0.953103046	3.11E-168	0.036809721	0.010087233	1.66E-173	0.143945	0.046896954
ASGR2	rs72842814	2.61E-33	0.953115229	1.08E-34	0.039279215	0.007605557	9.46E-40	0.348796	0.046884772
SNCG	rs192405960	2.31E-132	0.953153042	1.08E-133	0.044554572	0.002292386	1.85E-139	0.96839	0.046846958
FGFBP3	rs10748571	1.09E-301	0.953166455	3.74E-303	0.03270911	0.014124435	3.16E-308	0.0269495	0.046833545
PCDH9	rs9540915	6.23E-92	0.953206761	2.85E-93	0.043604914	0.003188325	7.09E-99	0.550739	0.046793239
PCDH9	rs557410163	6.23E-92	0.953207264	2.85E-93	0.043604408	0.003188327	7.09E-99	0.550739	0.046792735
SCARA5	rs7007499	5.82E-302	0.953286866	2.59E-303	0.042368043	0.004345091	3.16E-308	0.202651	0.046713134
SBSN	rs190874898	1.23E-21	0.953303573	5.55E-23	0.043009887	0.00368654	4.30E-28	0.551047	0.046696427
SNCG	rs544015004	7.98E-302	0.953352165	3.67E-303	0.043897813	0.002750021	3.16E-308	0.190811	0.046647834
AAMDC	rs547866890	1.34E-302	0.953353109	5.70E-304	0.040587574	0.006059317	3.16E-308	0.166555	0.046646891

OXT	rs146693630	3.62E-115	0.953402946	1.68E-116	0.044291564	0.00230549	6.41E-122	0.956646	0.046597054
SBSN	rs141955614	1.23E-21	0.95343877	5.53E-23	0.042874167	0.003687063	4.30E-28	0.551047	0.04656123
CEMIP2	rs72737956	2.06E-66	0.953493249	9.12E-68	0.042278925	0.004227827	9.03E-73	0.7141	0.046506752
FGFBP3	rs192232174	1.09E-301	0.95349406	3.70E-303	0.03237665	0.014129289	3.16E-308	0.0269495	0.046505939
HBZ	rs530834100	9.08E-303	0.953498355	3.79E-304	0.039833273	0.006668372	3.16E-308	0.00766885	0.046501645
PVALB	rs10854693	5.35E-302	0.953523775	2.34E-303	0.041708167	0.004768058	3.16E-308	0.0760764	0.046476225
PVALB	rs145418415	5.35E-302	0.953569956	2.34E-303	0.041661755	0.004768289	3.16E-308	0.0760764	0.046430044
PCDH9	rs183911966	6.24E-92	0.953594623	2.83E-93	0.043215754	0.003189623	7.09E-99	0.550739	0.046405377
CLEC4C	rs111973466	9.39E-303	0.953611725	4.02E-304	0.040781624	0.005606651	3.16E-308	0.821586	0.046388275
COMT	rs11705021	7.09E-302	0.953635383	3.01E-303	0.040563747	0.00580087	3.16E-308	0.0698747	0.046364617
SHBG	rs858519	2.78E-301	0.953656882	1.19E-302	0.040759627	0.005583491	3.16E-308	0.14582	0.046343118
PVALB	rs760521	5.35E-302	0.953728221	2.33E-303	0.041502699	0.00476908	3.16E-308	0.0760764	0.046271779
AAMDC	rs567783044	9.01E-303	0.953751781	3.85E-304	0.040746454	0.005501765	3.16E-308	0.166555	0.046248219
AAMDC	rs566391077	1.30E-302	0.953761228	5.48E-304	0.040226876	0.006011897	3.16E-308	0.166555	0.046238773
CLEC4C	rs140122594	9.40E-303	0.953845608	3.99E-304	0.040546365	0.005608027	3.16E-308	0.821586	0.046154392
ANXA2	rs143857439	1.01E-301	0.953940013	4.21E-303	0.039791671	0.006268315	3.16E-308	0.0675632	0.046059986
SHBG	rs557369023	2.78E-301	0.953969313	1.18E-302	0.040445367	0.00558532	3.16E-308	0.14582	0.046030687

AAMDC	rs377595062	1.63E-302	0.953975341	6.82E-304	0.039774531	0.006250128	3.16E-308	0.166555	0.046024659
LEPR	rs78007650	1.35E-16	0.95398257	5.96E-18	0.04223061	0.00378682	6.53E-23	0.800158	0.04601743
SHBG	rs1050541	2.78E-301	0.954108048	1.18E-302	0.040305819	0.005586132	3.16E-308	0.14582	0.045891951
SCARA5	rs12548086	5.83E-302	0.954113212	2.54E-303	0.04153793	0.004348858	3.16E-308	0.202651	0.045886788
SCARA5	rs2726953	5.83E-302	0.954115434	2.54E-303	0.041535698	0.004348868	3.16E-308	0.202651	0.045884566
HBZ	rs377576756	9.08E-303	0.954277306	3.72E-304	0.039048875	0.00667382	3.16E-308	0.00766885	0.045722695
ANXA2	rs67827628	3.92E-302	0.954310905	1.63E-303	0.039696206	0.005992889	3.16E-308	0.0675632	0.045689095
ALDH3A1	rs540759810	2.17E-302	0.954400415	8.95E-304	0.039333156	0.006266429	3.16E-308	0.0355795	0.045599585
PVALB	rs183420301	5.35E-302	0.954479719	2.29E-303	0.040747444	0.004772838	3.16E-308	0.0760764	0.045520282
IDO1	rs72642842	1.50E-252	0.954577717	6.42E-254	0.040776711	0.004645572	7.29E-260	0.267346	0.045422283
RNASET2	rs2769343	3.20E-303	0.954625234	1.43E-304	0.042721551	0.002653215	3.16E-308	0.0693538	0.045374766
AAMDC	rs553269512	8.90E-303	0.95465195	3.71E-304	0.039845365	0.005502685	3.16E-308	0.166555	0.04534805
RELT	rs548308464	1.05E-301	0.954696501	4.09E-303	0.037051345	0.008252154	3.16E-308	0.880733	0.045303499
ALDH3A1	rs186253238	2.17E-302	0.954773982	8.86E-304	0.038957136	0.006268882	3.16E-308	0.0355795	0.045226018
GZMB	rs8192921	8.29E-302	0.954852285	3.42E-303	0.039388343	0.005759372	3.16E-308	0.159244	0.045147715
ALDH3A1	rs72840375	2.17E-302	0.954870476	8.84E-304	0.038860008	0.006269515	3.16E-308	0.0355795	0.045129523
IDO1	rs72642842	4.09E-230	0.95490892	1.73E-231	0.040517523	0.004573558	3.23E-237	0.267346	0.045091081

GZMB	rs1951597	8.29E-302	0.954954052	3.41E-303	0.039285962	0.005759986	3.16E-308	0.159244	0.045045948
CLEC4C	rs539149918	9.48E-303	0.955044341	3.91E-304	0.039357901	0.005597758	3.16E-308	0.821586	0.044955659
AAMDC	rs187794842	9.12E-303	0.955055263	3.77E-304	0.039472369	0.005472368	3.16E-308	0.166555	0.044944737
CEMIP2	rs191912523	2.06E-66	0.955067087	8.78E-68	0.040698108	0.004234805	9.03E-73	0.7141	0.044932913
CLEC4C	rs79164925	9.00E-302	0.955108166	3.56E-303	0.037789771	0.007102063	3.16E-308	0.0727378	0.044891834
SPOCK1	rs533419807	8.15E-60	0.955185232	3.61E-61	0.042342866	0.002471902	4.49E-66	0.808997	0.044814768
SPOCK1	rs145696269	8.15E-60	0.955190164	3.61E-61	0.042337921	0.002471914	4.49E-66	0.808997	0.044809835
ANXA2	rs9920880	2.21E-301	0.955260195	9.28E-303	0.040112394	0.004627411	3.16E-308	0.231541	0.044739805
RNASET2	rs11159	3.21E-303	0.955280418	1.41E-304	0.042064547	0.002655036	3.16E-308	0.0693538	0.044719583
RNASET2	rs574079969	3.21E-303	0.955324683	1.41E-304	0.042020158	0.002655159	3.16E-308	0.0693538	0.044675317
CEMIP2	rs146439095	2.06E-66	0.955432933	8.70E-68	0.04033064	0.004236427	9.03E-73	0.7141	0.044567067
RNASET2	rs550433007	3.21E-303	0.95548824	1.40E-304	0.041856146	0.002655614	3.16E-308	0.0693538	0.04451176
SNCG	rs554951824	5.14E-303	0.955491238	2.22E-304	0.041301806	0.003206955	3.16E-308	0.514926	0.044508761
ANXA2	rs556386717	1.15E-103	0.955494467	4.40E-105	0.036626532	0.007879001	6.84E-111	0.406228	0.044505533
SPOCK1	rs150462663	8.15E-60	0.955512412	3.59E-61	0.042014839	0.002472748	4.49E-66	0.808997	0.044487587
IFNAR1	rs118032403	6.66E-301	0.955665643	2.76E-302	0.039671383	0.004662974	3.16E-308	0.17744	0.044334357
IFNAR1	rs141910822	1.00E-119	0.955668811	4.20E-121	0.040081262	0.004249927	7.69E-127	0.479779	0.044331189

SPOCK1	rs35662447	8.16E-60	0.95588742	3.55E-61	0.041638862	0.002473719	4.49E-66	0.808997	0.044112581
LY75	rs562045114	4.30E-303	0.955901031	1.71E-304	0.037934892	0.006164077	3.16E-308	0.0196006	0.044098969
PROK1	rs2030013	1.06E-302	0.95593812	4.51E-304	0.040773306	0.003288574	3.16E-308	0.269921	0.04406188
ALDH3A1	rs113005706	2.17E-302	0.955940526	8.59E-304	0.037782932	0.006276541	3.16E-308	0.0355795	0.044059473
LY75	rs184815971	6.03E-303	0.95603554	2.35E-304	0.0371748	0.00678966	3.16E-308	0.0248193	0.04396446
GZMB	rs117110598	8.30E-302	0.956055445	3.32E-303	0.038177926	0.005766629	3.16E-308	0.159244	0.043944555
BOLA2B	rs199954887	7.44E-10	0.956476593	2.33E-11	0.029903511	0.013619896	6.50E-16	0.244654	0.043523407
ANXA2	rs182787432	3.93E-302	0.956577412	1.54E-303	0.037415465	0.006007122	3.16E-308	0.0675632	0.043422587
BOLA2B	rs549777936	7.44E-10	0.956622009	2.32E-11	0.029756016	0.013621975	6.50E-16	0.244654	0.043377991
SNCG	rs545496591	5.03E-303	0.956625468	2.11E-304	0.040170031	0.003204501	3.16E-308	0.514926	0.043374532
LY75	rs539743626	6.17E-303	0.956629205	2.35E-304	0.03649252	0.006878275	3.16E-308	0.0248193	0.043370795
BCAT1	rs10505955	4.41E-253	0.956656542	1.86E-254	0.040329688	0.00301377	2.99E-260	0.640625	0.043343458
LY75	rs191406522	4.14E-303	0.956834689	1.61E-304	0.03709713	0.006068181	3.16E-308	0.0196006	0.043165311
SNCG	rs4933405	1.41E-302	0.95686389	5.97E-304	0.040633614	0.002502496	3.16E-308	0.190811	0.04313611
IDO1	rs183958352	1.51E-252	0.956889452	6.05E-254	0.038453725	0.004656823	7.29E-260	0.267346	0.043110548
ALDH3A1	rs8081130	2.18E-302	0.956892602	8.38E-304	0.036824605	0.006282792	3.16E-308	0.0355795	0.043107397
FCRLB	rs148220562	3.01E-302	0.956922447	1.17E-303	0.037318448	0.005759105	3.16E-308	0.12956	0.043077553

IDO1	rs183958352	1.59E-234	0.956928403	6.41E-236	0.038494357	0.00457724	1.26E-241	0.267346	0.043071597
BOLA2B	rs76840156	7.44E-10	0.956953328	2.29E-11	0.029420003	0.013626668	6.50E-16	0.244654	0.043046671
IDO1	rs183958352	4.10E-230	0.957294169	1.63E-231	0.038120849	0.004584982	3.23E-237	0.267346	0.042705831
IDO1	rs183958352	5.37E-244	0.957308347	2.15E-245	0.03828111	0.004410542	2.02E-251	0.294061	0.042691652
LY75	rs146610977	2.47E-303	0.957443659	9.72E-305	0.037673008	0.004883333	3.16E-308	0.0358443	0.042556341
PROK1	rs17025957	1.16E-302	0.957617632	4.74E-304	0.039128108	0.00325426	3.16E-308	0.269921	0.042382368
AAMDC	rs189625557	2.63E-302	0.957651297	1.00E-303	0.036401493	0.00594721	3.16E-308	0.213588	0.042348703
PCDH9	rs73205668	8.62E-19	0.957827295	3.55E-20	0.039445781	0.002726924	8.62E-25	0.77714	0.042172705
ANXA2	rs190776828	3.94E-302	0.957886486	1.48E-303	0.036098171	0.006015343	3.16E-308	0.0675632	0.042113514
CCN1	rs1324899	4.31E-08	0.95791413	1.79E-09	0.039725358	0.002360467	1.21E-13	0.956128	0.042085825
ASAH2	rs2842131	2.31E-302	0.957941764	8.17E-304	0.033837828	0.008220409	3.16E-308	0.0684573	0.042058237
PROK1	rs12034102	1.06E-302	0.958082021	4.27E-304	0.038622029	0.00329595	3.16E-308	0.269921	0.041917979
ASAH2	rs66941844	2.31E-302	0.9584461	8.04E-304	0.033329164	0.008224736	3.16E-308	0.0684573	0.0415539
SMPD1	rs140578410	4.34E-301	0.958458652	1.76E-302	0.038936185	0.002605163	3.16E-308	0.753744	0.041541348
FCRLB	rs79274796	1.42E-302	0.958689161	5.32E-304	0.035884279	0.00542656	3.16E-308	0.125692	0.041310839
BCAT1	rs2200509	4.42E-253	0.958708303	1.76E-254	0.038271463	0.003020233	2.99E-260	0.640625	0.041291696
LY75	rs190205452	2.46E-303	0.958711698	9.36E-305	0.03653831	0.004749991	3.16E-308	0.0634658	0.041288301

IFNAR1	rs372843123	5.02E-51	0.958728928	1.65E-52	0.031462109	0.009808963	9.76E-58	0.0897243	0.041271072
CLEC4C	rs778658317	2.86E-284	0.958815592	1.10E-285	0.036717782	0.004466626	7.33E-292	0.268069	0.041184408
HBZ	rs191020655	1.06E-302	0.958941883	3.74E-304	0.033764826	0.007293291	3.16E-308	0.00766885	0.041058117
LY75	rs530160229	2.46E-303	0.959029448	9.31E-305	0.036242414	0.004728138	3.16E-308	0.0634658	0.040970552
LY75	rs191815062	2.43E-303	0.959051831	9.15E-305	0.036093634	0.004854535	3.16E-308	0.0257953	0.040948169
SHBG	rs75862036	1.21E-224	0.959311159	4.81E-226	0.038045363	0.002643478	8.45E-232	0.985708	0.040688841
AAMDC	rs546341113	5.34E-302	0.959311473	1.92E-303	0.034570552	0.006117975	3.16E-308	0.0749739	0.040688527
AAMDC	rs527589	4.61E-302	0.959318452	1.66E-303	0.034594844	0.006086704	3.16E-308	0.0749739	0.040681548
BCAT1	rs11047639	4.42E-253	0.959318914	1.74E-254	0.037658929	0.003022157	2.99E-260	0.640625	0.040681086
AAMDC	rs538140362	3.75E-302	0.959330487	1.36E-303	0.034696779	0.005972734	3.16E-308	0.213588	0.040669513
ITGB7	rs11170486	1.91E-35	0.959392399	7.23E-37	0.036292039	0.004315561	5.25E-42	0.481581	0.0406076
LY75	rs183403691	2.98E-303	0.959430266	1.09E-304	0.035237045	0.005332689	3.16E-308	0.0196006	0.040569734
HBZ	rs571504277	1.07E-302	0.959528278	3.72E-304	0.033370565	0.007101158	3.16E-308	0.00766885	0.040471723
B4GALT1	rs190558555	8.90E-160	0.959528798	3.35E-161	0.036094613	0.004376588	3.38E-167	0.513737	0.040471201
B4GALT1	rs7019909	8.90E-160	0.959528798	3.35E-161	0.036094613	0.004376588	3.38E-167	0.513737	0.040471201
LY75	rs193122968	2.98E-303	0.959565666	1.09E-304	0.035100892	0.005333442	3.16E-308	0.0196006	0.040434334
LY75	rs536254819	2.93E-303	0.959588562	1.07E-304	0.035124534	0.005286903	3.16E-308	0.0196006	0.040411437

B4GALT1	rs184789571	8.91E-160	0.959593944	3.34E-161	0.036029171	0.004376886	3.38E-167	0.513737	0.040406057
BCAT1	rs1872647	4.43E-253	0.960211282	1.70E-254	0.036763749	0.003024968	2.99E-260	0.640625	0.039788717
SPON1	rs2618513	1.56E-302	0.960756991	5.94E-304	0.03659325	0.002649758	3.16E-308	0.294901	0.039243008
SERPING1	rs546390554	1.17E-106	0.960789844	4.31E-108	0.035317267	0.003892889	2.76E-113	0.341729	0.039210156
MST1	rs182158322	5.84E-303	0.960854471	2.16E-304	0.035518565	0.003626964	3.16E-308	0.596661	0.039145529
ITGB7	rs7965179	1.91E-35	0.960861842	6.94E-37	0.034815987	0.004322171	5.25E-42	0.481581	0.039138158
SPON1	rs1819084	1.56E-302	0.960976834	5.91E-304	0.036372802	0.002650365	3.16E-308	0.294901	0.039023167
SERPING1	rs12790660	4.24E-85	0.961288845	1.60E-86	0.03629291	0.002418244	8.25E-92	0.884668	0.038711154
MST1	rs141786906	5.64E-303	0.96143459	2.04E-304	0.034821575	0.003743835	3.16E-308	0.596661	0.03856541
SERPING1	rs622969	4.24E-85	0.961672203	1.58E-86	0.035908588	0.002419209	8.25E-92	0.884668	0.038327797
MST1	rs147365565	4.62E-303	0.961704885	1.64E-304	0.034232744	0.004062371	3.16E-308	0.596661	0.038295115
MST1	rs146998023	4.86E-303	0.962006123	1.72E-304	0.034004787	0.003989091	3.16E-308	0.596661	0.037993878
FCRLB	rs61801011	1.26E-302	0.962066125	4.24E-304	0.03247717	0.005456705	3.16E-308	0.125692	0.037933875
MST1	rs188471193	7.86E-303	0.962134384	2.85E-304	0.034861055	0.003004561	3.16E-308	0.596661	0.037865616
HBZ	rs562975124	1.07E-302	0.962138621	3.43E-304	0.030741513	0.007119866	3.16E-308	0.00766885	0.037861379
MST1	rs541522597	8.72E-303	0.962185037	3.15E-304	0.034781079	0.003033884	3.16E-308	0.596661	0.037814963
ITGB7	rs12232003	1.92E-35	0.962220886	6.66E-37	0.03345083	0.004328285	5.25E-42	0.481581	0.037779115

MST1	rs143827255	8.38E-303	0.962461833	3.01E-304	0.034535481	0.003002686	3.16E-308	0.596661	0.037538167
MST1	rs567593792	1.05E-302	0.962487877	3.76E-304	0.034386461	0.003125661	3.16E-308	0.596661	0.037512122
MST1	rs560940989	4.61E-303	0.96264903	1.59E-304	0.033288229	0.004062742	3.16E-308	0.596661	0.037350971
ITGB7	rs61754162	1.92E-35	0.962914329	6.53E-37	0.032754268	0.004331404	5.25E-42	0.481581	0.037085672
MST1	rs537367882	9.83E-303	0.963145216	3.44E-304	0.033758188	0.003096596	3.16E-308	0.596661	0.036854784
AOC3	rs535563803	1.34E-301	0.963419949	4.33E-303	0.031176222	0.005403828	3.16E-308	0.2716	0.03658005
SNCG	rs555434629	4.73E-303	0.963439206	1.64E-304	0.033353086	0.003207708	3.16E-308	0.514926	0.036560794
FCRLB	rs541461596	1.26E-302	0.96349177	4.05E-304	0.031043426	0.005464805	3.16E-308	0.125692	0.036508231
SERPING1	rs577363352	7.78E-133	0.96362039	2.46E-134	0.030485992	0.005893618	1.53E-140	0.587736	0.03637961
SPON1	rs117443940	2.72E-53	0.963774411	8.85E-55	0.031371678	0.004853911	1.10E-59	0.507269	0.036225589
FCRLB	rs35002732	1.25E-302	0.964293326	3.93E-304	0.030244147	0.005462527	3.16E-308	0.125692	0.035706674
MST1	rs7432077	8.57E-303	0.964328982	2.52E-304	0.028386075	0.007284944	3.16E-308	0.914625	0.035671019
MST1	rs141259336	7.63E-303	0.964403375	2.26E-304	0.028569691	0.007026934	3.16E-308	0.914625	0.035596625
FCRLB	rs61803026	1.26E-302	0.964425977	3.93E-304	0.030103908	0.005470114	3.16E-308	0.125692	0.035574022
SNCG	rs7074886	4.76E-303	0.964619928	1.59E-304	0.032143453	0.003236619	3.16E-308	0.514926	0.035380072
FLRT2	rs1625836	4.58E-128	0.964709395	1.54E-129	0.032500536	0.002790069	4.29E-134	0.920923	0.035290605
MST1	rs141507843	6.87E-303	0.964713379	2.02E-304	0.02835896	0.00692766	3.16E-308	0.914625	0.03528662

FCRLB	rs61801194	1.25E-302	0.964752095	3.87E-304	0.029782771	0.005465134	3.16E-308	0.125692	0.035247905
MST1	rs75840552	7.12E-303	0.964770937	2.09E-304	0.02827541	0.006953653	3.16E-308	0.914625	0.035229063
MST1	rs114128877	4.44E-303	0.964868504	1.43E-304	0.031023134	0.004108361	3.16E-308	0.596661	0.035131495
FCRLB	rs72704099	1.25E-302	0.964985828	3.84E-304	0.029547722	0.00546645	3.16E-308	0.125692	0.035014172
MST1	rs533344903	4.53E-303	0.964994732	1.45E-304	0.030842535	0.004162733	3.16E-308	0.596661	0.035005268
MST1	rs578130028	4.38E-303	0.965045083	1.40E-304	0.030861468	0.004093449	3.16E-308	0.596661	0.034954917
MST1	rs563209905	6.01E-303	0.965335038	1.76E-304	0.028184092	0.00648087	3.16E-308	0.914625	0.034664962
SERPING1	rs28362944	7.79E-133	0.965384195	2.32E-134	0.028711399	0.005904406	1.53E-140	0.587736	0.034615805
ALDH3A1	rs144011694	9.29E-113	0.965453291	2.77E-114	0.028812728	0.005733981	8.24E-120	0.222518	0.034546709
SNCG	rs12780613	4.67E-303	0.965602589	1.51E-304	0.03114618	0.003251231	3.16E-308	0.514926	0.034397411
MST1	rs186331853	5.83E-303	0.965848986	1.67E-304	0.027676549	0.006474465	3.16E-308	0.914625	0.034151014
SNCG	rs553247470	4.92E-303	0.965889981	1.57E-304	0.030808542	0.003301477	3.16E-308	0.514926	0.034110019
MST1	rs115644657	5.66E-303	0.966381503	1.59E-304	0.027168531	0.006449966	3.16E-308	0.914625	0.033618497
PRSS8	rs889555	4.99E-43	0.966658084	1.59E-44	0.030851315	0.002490601	5.71E-49	0.882627	0.033341916
PRSS8	rs562177063	4.99E-43	0.966952073	1.58E-44	0.030556568	0.002491359	5.71E-49	0.882627	0.033047927
IDO1	rs78754071	2.56E-28	0.967816315	6.89E-30	0.026024125	0.00615956	1.21E-34	0.418616	0.032183685
SNCG	rs144099171	5.34E-303	0.967817482	1.59E-304	0.028809956	0.003372562	3.16E-308	0.514926	0.032182518

MST1	rs74680554	4.26E-303	0.968078052	1.21E-304	0.027493311	0.004428637	3.16E-308	0.0723369	0.031921948
MST1	rs184211666	4.52E-303	0.968155952	1.21E-304	0.025889656	0.005954391	3.16E-308	0.914625	0.031844047
HBZ	rs140603557	1.12E-302	0.968473094	2.81E-304	0.024188035	0.007338871	3.16E-308	0.00766885	0.031526906
MST1	rs72554696	4.14E-303	0.968506193	1.10E-304	0.025601729	0.005892077	3.16E-308	0.914625	0.031493806
MST1	rs571299348	4.22E-303	0.968522303	1.11E-304	0.025572972	0.005904725	3.16E-308	0.914625	0.031477697
HBZ	rs216587	1.12E-302	0.968611451	2.79E-304	0.024048629	0.00733992	3.16E-308	0.00766885	0.031388549
HBZ	rs554118213	1.12E-302	0.968638458	2.79E-304	0.024021418	0.007340124	3.16E-308	0.00766885	0.031361542
MST1	rs75779287	3.85E-303	0.968839075	1.06E-304	0.026611819	0.004549105	3.16E-308	0.0723369	0.031160924
DEFB104B	rs370953550	5.04E-73	0.96932621	9.93E-75	0.019070041	0.011603748	2.24E-80	0.852595	0.030673789
MST1	rs145925315	3.78E-303	0.969615155	9.76E-305	0.025037122	0.005347723	3.16E-308	0.916052	0.030384845
MST1	rs185065281	3.74E-303	0.969668983	9.92E-305	0.025734922	0.004596095	3.16E-308	0.916052	0.030331017
MST1	rs189982577	3.77E-303	0.969691243	9.72E-305	0.024961924	0.005346833	3.16E-308	0.916052	0.030308757
MST1	rs527423135	3.70E-303	0.969696689	9.82E-305	0.025714826	0.004588485	3.16E-308	0.916052	0.030303311
MST1	rs539999381	3.89E-303	0.969821263	9.89E-305	0.024613563	0.005565174	3.16E-308	0.914625	0.030178737
MST1	rs78026743	3.59E-303	0.969994865	9.24E-305	0.024938003	0.005067132	3.16E-308	0.916052	0.030005135
MST1	rs111739368	3.45E-303	0.970137601	8.93E-305	0.025076893	0.004785506	3.16E-308	0.916052	0.029862399
MST1	rs114834839	3.53E-303	0.970343135	9.10E-305	0.025030187	0.004626679	3.16E-308	0.916052	0.029656866

COMT	rs116931143	5.44E-172	0.972600232	1.35E-173	0.024235278	0.003164491	3.22E-179	0.446521	0.027399769
ASAH2	rs200859624	2.31E-302	0.973335852	4.36E-304	0.018415176	0.008248972	3.16E-308	0.0684573	0.026664148
SNCG	rs117795997	5.69E-303	0.974557371	1.28E-304	0.022000419	0.00344221	3.16E-308	0.514926	0.025442629
SNCG	rs183082741	5.70E-303	0.97537282	1.24E-304	0.021171094	0.003456086	3.16E-308	0.514926	0.02462718
SNCG	rs117548383	2.03E-301	0.978116599	3.88E-303	0.01870828	0.003175121	3.16E-308	0.443603	0.021883401
GUSB	rs187629615	5.41E-57	0.979482642	8.56E-59	0.015491634	0.005025725	2.64E-63	0.227511	0.020517359
SNCG	rs553880179	1.28E-302	0.97996917	2.21E-304	0.016929526	0.003101304	3.16E-308	0.249963	0.02003083
SNCG	rs565858129	2.86E-302	0.981641145	4.51E-304	0.015466867	0.002891988	3.16E-308	0.443603	0.018358855
SNCG	rs2478634	2.55E-302	0.981790076	3.98E-304	0.015307067	0.002902858	3.16E-308	0.443603	0.018209925
DEFB104B	rs561132918	5.11E-73	0.981946599	3.28E-75	0.006298574	0.011754826	2.24E-80	0.852595	0.0180534
DEFB104B	rs183772362	5.14E-73	0.987295332	4.67E-76	0.000885812	0.011818856	2.24E-80	0.852595	0.012704668
ASAH2	rs184446700	9.91E-301	0.988523375	8.09E-303	0.008063598	0.003413027	3.16E-308	0.850637	0.011476625
ASAH2	rs72797721	1.28E-300	0.989239259	1.03E-302	0.008007903	0.002752838	3.16E-308	0.850637	0.010760741
DEFB104B	rs149510469	0.998053229	0.001865324	3.97E-05	3.26E-08	4.17E-05	0.002225974	0.474096	4.17326E-05

Supplement table4.R11_BLADDER_CANCER.decode.coloc.results

protein	SNP	PP.H0	PP.H1	PP.H2	PP.H3	PP.H4	p1	p2	PP.H3+PP.H4
**NOV**	**rs11779998**	**3.20E-13**	**0.131801675**	**2.63E-13**	**0.107667811**	**0.760530514**	**7.29E-17**	**9.53E-05**	**0.868198325**
**GSTM3**	**rs572141660**	**2.44E-302**	**0.293371658**	**4.79E-303**	**0.057060119**	**0.649568223**	**3.16E-308**	**1.38E-05**	**0.706628342**
GSTM3	rs139606823	2.00E-302	0.373849225	4.04E-303	0.074927476	0.551223299	3.16E-308	1.38E-05	0.626150775
GSTM3	rs72705214	2.00E-302	0.37394262	4.03E-303	0.074696374	0.551361006	3.16E-308	1.38E-05	0.62605738
GSTM3	rs571615814	2.00E-302	0.374068595	4.01E-303	0.074384655	0.55154675	3.16E-308	1.38E-05	0.625931405
GSTM3	rs11101981	2.00E-302	0.374294173	3.98E-303	0.073826474	0.551879354	3.16E-308	1.38E-05	0.625705827
GSTM3	rs148886417	2.00E-302	0.374303266	3.98E-303	0.073803972	0.551892762	3.16E-308	1.38E-05	0.625696734
GSTM3	rs36210087	2.00E-302	0.374379842	3.97E-303	0.07361449	0.552005668	3.16E-308	1.38E-05	0.625620158
GSTM3	rs4970773	2.00E-302	0.374451329	3.96E-303	0.073437598	0.552111073	3.16E-308	1.38E-05	0.625548671
GSTM3	rs141513521	2.00E-302	0.374470432	3.96E-303	0.073390328	0.55213924	3.16E-308	1.38E-05	0.625529568
GSTM3	rs78191259	2.00E-302	0.374479687	3.96E-303	0.073367428	0.552152885	3.16E-308	1.38E-05	0.625520313
GSTM3	rs6676659	2.00E-302	0.374489711	3.95E-303	0.073342622	0.552167666	3.16E-308	1.38E-05	0.625510289
GSTM3	rs58676655	2.00E-302	0.37458777	3.94E-303	0.073099982	0.552312249	3.16E-308	1.38E-05	0.62541223
KIAA1467	rs181227539	2.33E-12	0.378361616	3.69E-12	0.599452134	0.02218625	8.50E-19	0.453556	0.621638384

KIAA1467	rs4551855	3.91E-71	0.396937489	5.86E-71	0.594608788	0.008453723	1.44E-78	0.363101	0.603062511
KIAA1467	rs541868750	9.87E-71	0.428461511	1.30E-70	0.562584019	0.00895447	1.44E-78	0.363101	0.571538489
VASN	rs757593	9.61E-12	0.576856419	2.34E-12	0.140137262	0.283006319	8.51E-18	0.00107503	0.423143581
GSTM3	rs530794766	4.35E-302	0.591970625	6.72E-303	0.091022677	0.317006698	3.16E-308	0.000236347	0.408029375
RHOC	rs10745330	1.68E-28	0.69261264	1.43E-29	0.058904241	0.248483119	2.92E-34	0.00128585	0.30738736
RBP4	rs190355820	1.94E-05	0.713715921	1.28E-06	0.046906647	0.239356735	1.22E-26	0.016778	0.286263382
RGMA	rs144254820	6.50E-13	0.760219169	4.88E-14	0.056828728	0.182952102	5.66E-22	0.0396424	0.239780831
MPG	rs149439168	5.05E-17	0.763835158	3.90E-18	0.058809855	0.177354988	2.69E-22	0.00295141	0.236164842
BPHL	rs200978408	4.51E-37	0.778244163	7.10E-38	0.122373919	0.099381918	4.93E-42	0.0097807	0.221755837
BPHL	rs9503407	4.51E-37	0.778545819	7.08E-38	0.122033742	0.099420439	4.93E-42	0.0097807	0.221454181
MPG	rs2562156	5.05E-17	0.778776683	2.16E-18	0.033089818	0.1881335	2.69E-22	0.00295141	0.221223317
KIAA1467	rs74399808	7.80E-71	0.792216314	1.88E-71	0.190911566	0.016872121	1.44E-78	0.363101	0.207783686
QSOX2	rs117491627	1.40E-300	0.800598278	1.87E-301	0.106636456	0.092765267	3.16E-308	0.00539163	0.199401722
QSOX2	rs554519060	1.41E-300	0.80279176	1.83E-301	0.104188815	0.093019425	3.16E-308	0.00539163	0.19720824
QSOX2	rs7866274	1.41E-300	0.803550421	1.81E-301	0.103342248	0.093107331	3.16E-308	0.00539163	0.196449579
QSOX2	rs79287014	1.41E-300	0.803577164	1.81E-301	0.103312406	0.09311043	3.16E-308	0.00539163	0.196422836
QSOX2	rs3812580	1.41E-300	0.806143074	1.76E-301	0.100449184	0.093407742	3.16E-308	0.00539163	0.193856926

MFGE8	rs142839077	1.23E-171	0.806210224	9.74E-173	0.063592895	0.130196881	6.76E-179	0.00439542	0.193789776
QSOX2	rs10858248	1.41E-300	0.807673538	1.73E-301	0.098741386	0.093585077	3.16E-308	0.00539163	0.192326462
MFGE8	rs143127748	1.24E-171	0.808126821	9.40E-173	0.061366782	0.130506397	6.76E-179	0.00439542	0.191873179
MFGE8	rs149096162	1.24E-171	0.812352325	8.65E-173	0.056458891	0.131188784	6.76E-179	0.00439542	0.187647675
MFGE8	rs201988637	1.24E-171	0.812507395	8.62E-173	0.056278778	0.131213827	6.76E-179	0.00439542	0.187492605
STX4	rs147406120	9.00E-07	0.812737748	5.06E-08	0.045521736	0.141739565	2.02E-13	0.0241635	0.187261301
MFGE8	rs2271714	1.24E-171	0.813333782	8.48E-173	0.055318936	0.131347282	6.76E-179	0.00439542	0.186666218
MFGE8	rs34239095	1.24E-171	0.813512313	8.44E-173	0.055111573	0.131376114	6.76E-179	0.00439542	0.186487687
QSOX2	rs12345528	1.42E-300	0.813988095	1.61E-301	0.091695161	0.094316744	3.16E-308	0.00539163	0.186011905
QSOX2	rs112337563	1.43E-300	0.81400834	1.61E-301	0.09167257	0.09431909	3.16E-308	0.00539163	0.18599166
MFGE8	rs146518381	1.26E-171	0.822022001	6.93E-173	0.045227635	0.132750364	6.76E-179	0.00439542	0.177977999
PLOD3	rs541042659	7.10E-66	0.832826385	7.71E-67	0.090346854	0.076826761	1.02E-80	0.0752593	0.167173615
PLAT	rs2020921	9.61E-136	0.832852105	7.94E-137	0.068690119	0.098457776	3.63E-144	0.0346633	0.167147895
KIAA1467	rs144268071	8.23E-71	0.835000353	1.45E-71	0.147216453	0.017783194	1.44E-78	0.363101	0.164999647
KIAA1467	rs117172138	8.23E-71	0.835048169	1.45E-71	0.147167504	0.017784326	1.44E-78	0.363101	0.164951831
IGDCC4	rs141417880	1.32E-289	0.836358301	1.20E-290	0.075973659	0.087668039	3.16E-308	0.0332744	0.163641699
IGDCC4	rs112819832	1.54E-289	0.836389625	1.43E-290	0.077891715	0.085718661	3.16E-308	0.0332744	0.163610375

IGDCC4	rs148273100	1.32E-289	0.837849402	1.17E-290	0.074326259	0.087824338	3.16E-308	0.0332744	0.162150598
PSAP	rs11000234	1.83E-23	0.838024492	1.16E-24	0.052777608	0.1091979	1.17E-29	0.027325	0.161975508
PLAT	rs74702344	9.69E-136	0.839833301	7.04E-137	0.060883623	0.099283076	3.63E-144	0.0346633	0.160166699
EPHA5	rs2122718	3.35E-24	0.841375992	5.84E-25	0.146694211	0.011929796	7.88E-30	0.147245	0.158624008
IGDCC4	rs189123360	1.33E-289	0.842530797	1.09E-290	0.069154156	0.088315047	3.16E-308	0.0332744	0.157469203
IGDCC4	rs148990036	1.33E-289	0.842841137	1.08E-290	0.068811285	0.088347578	3.16E-308	0.0332744	0.157158863
IGDCC4	rs187219517	1.68E-39	0.843839064	1.48E-40	0.074114963	0.082045973	3.63E-49	0.0899146	0.156160936
IGDCC4	rs35223184	1.33E-289	0.844459121	1.06E-290	0.067023702	0.088517177	3.16E-308	0.0332744	0.155540879
IGDCC4	rs78236773	1.33E-289	0.844879904	1.05E-290	0.066558812	0.088561284	3.16E-308	0.0332744	0.155120096
EPHA5	rs151144506	2.39E-19	0.844997196	4.29E-20	0.151296825	0.003705979	3.85E-25	0.77766	0.155002804
IGDCC4	rs183894976	1.33E-289	0.84507829	1.05E-290	0.066339631	0.088582079	3.16E-308	0.0332744	0.15492171
APOC1	rs2627646	8.63E-19	0.848729772	8.70E-20	0.085447134	0.065823094	2.30E-25	0.0560248	0.151270228
IGDCC4	rs191927852	1.34E-289	0.849415206	9.70E-291	0.061548114	0.089036679	3.16E-308	0.0332744	0.150584794
IGDCC4	rs8034057	1.34E-289	0.850106026	9.58E-291	0.060784882	0.089109092	3.16E-308	0.0332744	0.149893974
KLRB1	rs139517772	4.62E-19	0.851423137	2.20E-20	0.040512448	0.108064415	2.29E-25	0.00722321	0.148576863
IGDCC4	rs72741403	1.34E-289	0.852341256	9.20E-291	0.058315353	0.089343391	3.16E-308	0.0332744	0.147658744
IGDCC4	rs181169068	1.34E-289	0.85346494	9.00E-291	0.057073883	0.089461177	3.16E-308	0.0332744	0.14653506

IGDCC4	rs189830428	1.34E-289	0.854231982	8.87E-291	0.056226438	0.089541579	3.16E-308	0.0332744	0.145768018
KIR2DL4	rs112358903	1.77E-37	0.855734489	1.61E-38	0.077920527	0.066344984	7.96E-44	0.563653	0.144265511
CRP	rs2808628	4.04E-72	0.860882531	6.42E-73	0.136739553	0.002377916	4.07E-78	0.604896	0.139117469
POFUT1	rs143714048	1.12E-107	0.861069543	5.92E-109	0.045518288	0.093412169	1.05E-132	0.183554	0.138930457
POFUT1	rs149604302	1.12E-107	0.861318436	5.89E-109	0.045242393	0.09343917	1.05E-132	0.183554	0.138681564
POFUT1	rs541720091	1.12E-107	0.861822526	5.81E-109	0.044683618	0.093493856	1.05E-132	0.183554	0.138177474
POFUT1	rs567940352	1.12E-107	0.862083349	5.78E-109	0.044394499	0.093522151	1.05E-132	0.183554	0.137916651
POFUT1	rs192159142	1.12E-107	0.862089677	5.77E-109	0.044387486	0.093522838	1.05E-132	0.183554	0.137910323
POFUT1	rs373419129	1.12E-107	0.862266573	5.75E-109	0.044191399	0.093542028	1.05E-132	0.183554	0.137733427
QSOX2	rs74588014	2.98E-36	0.866243815	3.55E-37	0.103123492	0.030632694	7.28E-49	0.642452	0.133756185
VASN	rs541550582	3.07E-06	0.866455899	3.45E-07	0.09716713	0.036373554	1.23E-22	0.488954	0.133540684
PLOD3	rs142309161	3.10E-90	0.866538011	2.26E-91	0.063013354	0.070448635	1.93E-108	0.251764	0.133461989
PLOD3	rs182483308	3.11E-90	0.867631256	2.22E-91	0.06183123	0.070537514	1.93E-108	0.251764	0.132368744
PDGFRA	rs2307050	9.34E-33	0.87530077	9.25E-34	0.086645533	0.038053698	3.18E-40	0.453269	0.12469923
PDGFRA	rs139236922	9.34E-33	0.875304316	9.25E-34	0.086641832	0.038053852	3.18E-40	0.453269	0.124695684
RBP4	rs116887052	3.79E-22	0.876620829	3.55E-23	0.081975532	0.041403639	3.84E-63	0.55257	0.123379171
EIF1AD	rs570418251	1.02E-11	0.876635764	5.34E-13	0.045704528	0.077659709	1.05E-19	0.13241	0.123364236

RBP4	rs10786115	3.80E-22	0.877078124	3.53E-23	0.081496639	0.041425237	3.84E-63	0.55257	0.122921876
QSOX2	rs11103248	1.98E-57	0.880638857	2.03E-58	0.090195328	0.029165815	7.46E-83	0.572217	0.119361143
QSOX2	rs116580427	6.68E-49	0.881472289	7.45E-50	0.098190538	0.020337173	9.10E-67	0.493483	0.118527711
IGDCC4	rs147018417	1.10E-60	0.88225086	9.60E-62	0.077300482	0.040448657	1.73E-68	0.168512	0.11774914
MFGE8	rs555501675	9.76E-05	0.885295066	8.19E-06	0.074279479	0.040319692	5.22E-11	0.724589	0.114599171
ST6GALNAC6	rs10819315	8.12E-39	0.886919387	8.20E-40	0.0894508	0.023629814	7.50E-46	0.431893	0.113080613
TACO1	rs138066238	1.68E-05	0.887349382	7.48E-07	0.039300076	0.073332946	2.62E-12	0.229928	0.112633022
GZMM	rs147053889	1.01E-14	0.887381254	8.89E-16	0.078049388	0.034569358	1.15E-21	0.385233	0.112618746
GZMM	rs150194571	1.01E-14	0.887728709	8.84E-16	0.077688397	0.034582894	1.15E-21	0.385233	0.112271291
ST6GALNAC6	rs139501224	8.13E-39	0.887759469	8.12E-40	0.088588336	0.023652196	7.50E-46	0.431893	0.112240531
TACO1	rs192454466	1.69E-05	0.88781149	7.38E-07	0.038799773	0.073371141	2.62E-12	0.229928	0.112170914
CRYGD	rs202233735	1.85E-37	0.888062823	1.74E-38	0.083133208	0.028803969	4.91E-44	0.0246837	0.111937177
ST6GALNAC6	rs61740973	8.14E-39	0.88828282	8.07E-40	0.088051041	0.023666139	7.50E-46	0.431893	0.11171718
GZMM	rs114537924	1.01E-14	0.889098469	8.68E-16	0.076265276	0.034636255	1.15E-21	0.385233	0.110901531
CRYGD	rs34570682	1.86E-37	0.889122919	1.71E-38	0.082038716	0.028838365	4.91E-44	0.0246837	0.110877081
GZMM	rs1599882	1.01E-14	0.889140281	8.68E-16	0.076221835	0.034637884	1.15E-21	0.385233	0.110859719
MANSC1	rs534396522	1.28E-08	0.889196235	1.22E-09	0.084938449	0.025865302	1.91E-14	0.102747	0.110803751

GZMM	rs200398398	1.01E-14	0.889448244	8.64E-16	0.075901875	0.034649881	1.15E-21	0.385233	0.110551756
CRAT	rs6478861	4.84E-20	0.889637543	5.86E-21	0.107690649	0.002671808	1.18E-25	0.752458	0.110362457
PDGFRA	rs149474954	1.13E-16	0.889964461	1.26E-17	0.098809199	0.01122634	2.97E-23	0.978919	0.110035539
POMC	rs6545951	1.05E-33	0.890037652	4.83E-35	0.040789944	0.069172403	5.50E-43	0.298472	0.109962348
GZMM	rs181948048	1.01E-14	0.890100164	8.56E-16	0.075224558	0.034675278	1.15E-21	0.385233	0.109899836
RBP4	rs11812231	3.85E-22	0.890348752	2.93E-23	0.067599226	0.042052022	3.84E-63	0.55257	0.109651248
HEXB	rs142512890	1.33E-189	0.890392946	7.43E-191	0.049779277	0.059827777	7.91E-197	0.0102934	0.109607054
QSOX2	rs138878730	4.06E-224	0.89084281	4.10E-225	0.089966032	0.019191158	1.48E-231	0.0361568	0.10915719
MANSC1	rs374877307	1.02E-08	0.893506898	9.47E-10	0.083148505	0.023344587	1.91E-14	0.102747	0.106493091
EFNB2	rs288667	2.25E-07	0.894304137	2.58E-08	0.102830447	0.002865166	1.85E-13	0.575452	0.105695613
EFNB2	rs840219	2.25E-07	0.894594786	2.58E-08	0.102539331	0.002865633	1.85E-13	0.575452	0.105404963
PLAT	rs77115522	4.31E-85	0.894897293	3.40E-86	0.07051749	0.034585217	1.96E-93	0.257979	0.105102707
UAP1	rs28384482	9.53E-13	0.895884352	8.38E-14	0.078816353	0.025299295	3.01E-22	0.498307	0.104115648
HEXB	rs13164140	1.34E-189	0.896293076	6.49E-191	0.043482702	0.060224222	7.91E-197	0.0102934	0.103706924
IGDCC4	rs557689101	4.30E-32	0.897233855	4.07E-33	0.084964097	0.017802049	5.69E-39	0.428801	0.102766145
UAP1	rs61747497	9.53E-13	0.897300841	8.20E-14	0.077184349	0.02551481	3.01E-22	0.498307	0.102699159
HEXB	rs1696980	1.34E-189	0.897687105	6.27E-191	0.041995004	0.06031789	7.91E-197	0.0102934	0.102312895

EPHB4	rs314366	5.66E-36	0.897734009	5.93E-37	0.094125109	0.008140882	7.19E-42	0.091029	0.102265991
ACLY	rs147160379	0.047374099	0.847917985	0.002543078	0.045460183	0.056704655	5.90E-09	0.350493	0.102164838
PDGFRA	rs552921178	1.46E-32	0.898098468	1.04E-33	0.063831462	0.03807007	3.18E-40	0.453269	0.101901532
KIR3DS1	rs191585166	3.18E-09	0.899085037	3.09E-10	0.087246965	0.013667995	1.15E-14	0.0560506	0.10091496
CRAT	rs9409310	4.89E-20	0.899765956	5.29E-21	0.09724694	0.002987104	1.18E-25	0.752458	0.100234044
ST6GALNAC6	rs72769820	3.70E-14	0.900256509	3.60E-15	0.087468397	0.012275094	2.28E-20	0.388666	0.099743491
KIR3DS1	rs183324776	3.19E-09	0.902129295	2.98E-10	0.084156461	0.01371424	1.15E-14	0.0560506	0.097870702
MFGE8	rs536168018	4.25E-14	0.902689255	3.50E-15	0.074275657	0.023035087	7.86E-24	0.230401	0.097310745
KIR3DS1	rs62124095	3.20E-09	0.903138696	2.94E-10	0.083131681	0.01372962	1.15E-14	0.0560506	0.0968613
KIR3DS1	rs113082063	3.20E-09	0.903140768	2.94E-10	0.083129578	0.013729651	1.15E-14	0.0560506	0.096859229
NUCB1	rs567783605	7.41E-51	0.90327903	7.64E-52	0.093018326	0.003702644	1.36E-56	0.598362	0.09672097
HYAL1	rs116482870	3.80E-30	0.904736053	2.62E-31	0.062247915	0.033016032	1.43E-36	0.038626	0.095263947
ACLY	rs530779792	0.067595811	0.834545356	0.002899403	0.035737124	0.059222307	4.00E-09	0.328057	0.094959431
HYAL1	rs11545105	3.80E-30	0.905078895	2.60E-31	0.061892561	0.033028544	1.43E-36	0.038626	0.094921105
MAN1A2	rs557718229	1.05E-95	0.905338086	9.19E-97	0.079497623	0.015164291	8.54E-103	0.0819125	0.094661914
GPD1	rs76829642	2.74E-05	0.905354637	1.63E-06	0.053749696	0.040866667	4.26E-13	0.415818	0.094616363
NUCB1	rs3826817	7.43E-51	0.905411823	7.46E-52	0.09087679	0.003711386	1.36E-56	0.598362	0.094588177

KIR3DS1	rs78928735	3.21E-09	0.905573621	2.86E-10	0.08065974	0.013766635	1.15E-14	0.0560506	0.094426376
IGDCC4	rs150167834	5.03E-34	0.905594344	4.93E-35	0.088698174	0.005707482	9.70E-41	0.646858	0.094405656
KIR3DS1	rs79002558	3.21E-09	0.905596236	2.85E-10	0.080636782	0.013766979	1.15E-14	0.0560506	0.094403761
KIR3DS1	rs574769826	3.21E-09	0.905655193	2.85E-10	0.080576928	0.013767876	1.15E-14	0.0560506	0.094344804
BDNF	rs116911082	0.000721624	0.90612813	5.19E-05	0.065116338	0.027982028	2.28E-09	0.0455177	0.093098366
KIR2DL4	rs611728	3.93E-214	0.907223746	3.85E-215	0.088894383	0.003881871	1.45E-221	0.282216	0.092776254
KIR3DS1	rs190014827	3.21E-09	0.907240827	2.80E-10	0.078967223	0.013791946	1.15E-14	0.0560506	0.092759169
EPHB4	rs116968729	5.72E-36	0.907452804	5.32E-37	0.084318182	0.008229014	7.19E-42	0.091029	0.092547196
KIR2DL4	rs191585166	3.93E-214	0.90799348	3.82E-215	0.088121356	0.003885165	1.45E-221	0.282216	0.09200652
KIR2DL4	rs200781151	3.94E-214	0.909152117	3.77E-215	0.086957761	0.003890122	1.45E-221	0.282216	0.090847883
KIR2DL4	rs587721662	3.94E-214	0.909466912	3.75E-215	0.086641619	0.003891469	1.45E-221	0.282216	0.090533088
QSOX2	rs556924226	2.78E-14	0.909557361	2.67E-15	0.087441052	0.003001587	1.16E-20	0.430828	0.090442639
KIR2DL4	rs2916049	3.94E-214	0.909582442	3.75E-215	0.086525594	0.003891963	1.45E-221	0.282216	0.090417558
PTPRU	rs72663870	8.60E-28	0.909612409	4.08E-29	0.043131882	0.047255709	3.03E-42	0.923625	0.090387591
JAG1	rs191355617	1.24E-09	0.910003437	9.12E-11	0.06672384	0.023272722	6.42E-16	0.031199	0.089996562
SMPDL3A	rs562812113	9.31E-06	0.910269657	4.43E-07	0.043258287	0.046462303	1.28E-11	0.985473	0.089720589
PTPRU	rs72649236	8.62E-28	0.910595255	4.01E-29	0.042320943	0.047083802	3.03E-42	0.923625	0.089404745

JAG1	rs2423507	1.24E-09	0.910686266	9.02E-11	0.066023547	0.023290185	6.42E-16	0.031199	0.089313732
KIR2DL4	rs12976350	3.95E-214	0.910904345	3.69E-215	0.085198036	0.00389762	1.45E-221	0.282216	0.089095655
KIR2DL4	rs372069945	3.95E-214	0.911102223	3.68E-215	0.08499931	0.003898466	1.45E-221	0.282216	0.088897777
KIR2DL4	rs370375186	3.95E-214	0.911256942	3.68E-215	0.084843929	0.003899128	1.45E-221	0.282216	0.088743058
FOLH1	rs376294786	4.78E-54	0.911583864	4.21E-55	0.080317959	0.008098177	4.95E-60	0.372068	0.088416136
NTF3	rs148590960	1.53E-21	0.912003663	1.41E-22	0.083931389	0.004064948	2.56E-27	0.805269	0.087996337
KIR2DL4	rs62124095	3.95E-214	0.912128052	3.64E-215	0.083969092	0.003902856	1.45E-221	0.282216	0.087871948
EPHB4	rs143785959	2.97E-35	0.912339801	2.63E-36	0.080747892	0.006912308	1.97E-41	0.129229	0.087660199
VEGFA	rs189619546	1.63E-283	0.912692218	1.46E-284	0.08124851	0.006059272	3.69E-291	0.153495	0.087307782
JAG1	rs1051412	1.25E-09	0.914127696	8.54E-11	0.062494105	0.023378198	6.42E-16	0.031199	0.085872303
STX4	rs535039107	9.38E-13	0.914529511	4.35E-14	0.042360279	0.04311021	9.95E-23	0.460844	0.085470489
KIR2DL4	rs2569657	3.96E-214	0.914936863	3.52E-215	0.081148262	0.003914874	1.45E-221	0.282216	0.085063137
QSOX2	rs555416913	2.80E-14	0.914974824	2.51E-15	0.082005711	0.003019465	1.16E-20	0.430828	0.085025176
VEGFA	rs6921438	1.64E-283	0.915760921	1.40E-284	0.078159435	0.006079645	3.69E-291	0.153495	0.084239079
EPHA5	rs62300408	1.09E-24	0.915893239	8.34E-26	0.070262545	0.013844215	7.88E-30	0.147245	0.084106761
KIR2DL4	rs190014827	3.97E-214	0.916312408	3.46E-215	0.079766832	0.00392076	1.45E-221	0.282216	0.083687592
KIR2DL4	rs180863682	3.97E-214	0.916316476	3.46E-215	0.079762747	0.003920777	1.45E-221	0.282216	0.083683524

KIR2DL4	rs201678781	3.97E-214	0.916376772	3.45E-215	0.079702193	0.003921035	1.45E-221	0.282216	0.083623228
KIR2DL4	rs202100911	3.97E-214	0.916495947	3.45E-215	0.079582508	0.003921545	1.45E-221	0.282216	0.083504053
EPHB4	rs56121056	4.12E-10	0.916607668	3.62E-11	0.080642498	0.002749833	4.18E-16	0.573987	0.083392331
JAG1	rs138469749	0.009916983	0.908085661	0.000569942	0.052159563	0.029267852	7.28E-09	0.591703	0.081427415
TMEM106A	rs534687604	3.61E-12	0.918641365	2.68E-13	0.068220969	0.013137666	1.10E-19	0.192306	0.081358635
NTF3	rs4766352	1.55E-21	0.919345159	1.29E-22	0.076557164	0.004097677	2.56E-27	0.805269	0.080654841
APOC1	rs5112	1.20E-291	0.919678891	1.01E-292	0.078032508	0.002288601	2.52E-299	0.829901	0.080321109
GPD1	rs35256655	1.40E-05	0.919715315	5.69E-07	0.037426374	0.042843775	4.26E-13	0.415818	0.080270148
GPD1	rs199902085	1.40E-05	0.919864347	5.67E-07	0.0372704	0.042850717	4.26E-13	0.415818	0.080121116
VEGFA	rs11757296	1.65E-283	0.920033399	1.32E-284	0.073858592	0.006108009	3.69E-291	0.153495	0.079966601
APOC1	rs438811	1.20E-291	0.920206472	1.01E-292	0.077503614	0.002289914	2.52E-299	0.829901	0.079793528
MAN1A2	rs1998921	9.68E-39	0.920682504	4.55E-40	0.043201759	0.036115737	6.99E-46	0.0848242	0.079317496
PSMB1	rs4710814	4.40E-302	0.921349192	3.13E-303	0.06542769	0.013223118	3.16E-308	0.0332407	0.078650808
LOXL3	rs138485343	2.36E-30	0.921976148	1.77E-31	0.06917603	0.008847822	6.96E-37	0.305985	0.078023852
NTF3	rs141567598	1.00E-16	0.92247708	8.00E-18	0.073775832	0.003747088	2.25E-22	0.918779	0.07752292
ADPRHL2	rs79158556	9.74E-121	0.922595828	5.78E-122	0.054738082	0.02266609	2.82E-140	0.51327	0.077404172
ACY1	rs121912698	7.97E-177	0.922749088	5.13E-178	0.059299924	0.017950987	3.77E-199	0.794501	0.077250912

ACY1	rs121912701	7.97E-177	0.922779215	5.12E-178	0.059269212	0.017951574	3.77E-199	0.794501	0.077220785
NTF3	rs563142712	1.55E-21	0.922904874	1.23E-22	0.07298159	0.004113536	2.56E-27	0.805269	0.077095126
RGMA	rs11074135	3.12E-79	0.923016392	2.53E-80	0.074757511	0.002226098	4.40E-86	0.991271	0.076983608
RGMA	rs11635798	3.12E-79	0.923262267	2.52E-80	0.074511042	0.002226691	4.40E-86	0.991271	0.076737733
PLAT	rs112917062	2.32E-48	0.923932423	1.35E-49	0.053839185	0.022228392	1.74E-55	0.310135	0.076067577
SAT2	rs4130509	1.42E-10	0.924159521	7.24E-12	0.047081879	0.0287586	7.30E-17	0.246116	0.075840478
TPST2	rs77332738	2.20E-61	0.924777954	1.65E-62	0.069331181	0.005890865	1.10E-67	0.117716	0.075222046
TPST2	rs2283824	2.20E-61	0.924933783	1.65E-62	0.06917436	0.005891858	1.10E-67	0.117716	0.075066217
RGMA	rs140443849	3.13E-79	0.924977376	2.46E-80	0.072791797	0.002230827	4.40E-86	0.991271	0.075022624
LOXL3	rs560214250	2.37E-30	0.925091826	1.69E-31	0.066031083	0.008877091	6.96E-37	0.305985	0.074908174
CST6	rs76584642	2.17E-71	0.925101006	9.98E-73	0.042461469	0.032437525	4.08E-78	0.0764258	0.074898994
CST6	rs574977680	0.001430666	0.923731774	6.94E-05	0.044749922	0.030018283	3.08E-09	0.116923	0.074768205
ACY1	rs190202562	8.00E-177	0.925409511	4.89E-178	0.056587746	0.018002743	3.77E-199	0.794501	0.074590489
EIF1AD	rs72930984	9.44E-110	0.925589194	4.03E-111	0.039459113	0.034951693	3.09E-118	0.0695409	0.074410806
LOXL3	rs17010021	2.37E-30	0.925681033	1.67E-31	0.065436222	0.008882745	6.96E-37	0.305985	0.074318967
GMPR	rs6939056	1.39E-302	0.925800363	1.02E-303	0.06763912	0.006560517	3.16E-308	0.0202717	0.074199637
GMPR	rs542540525	1.37E-302	0.92593968	1.00E-303	0.06741728	0.00664304	3.16E-308	0.0202717	0.07406032

GMPR	rs529478109	1.39E-302	0.926146467	1.01E-303	0.067478251	0.006375282	3.16E-308	0.0202717	0.073853533
CST6	rs72928860	2.18E-71	0.926267485	9.70E-73	0.041254089	0.032478426	4.08E-78	0.0764258	0.073732515
GMPR	rs544660681	2.35E-302	0.926524563	1.73E-303	0.068087496	0.005387941	3.16E-308	0.0611646	0.073475437
APOC1	rs10419829	9.22E-18	0.926679696	6.72E-19	0.067483889	0.005836415	1.71E-24	0.660958	0.073320304
LOXL3	rs115156688	2.39E-30	0.926699703	1.66E-31	0.064379625	0.008920673	6.96E-37	0.305985	0.073300297
GMPR	rs571244186	1.54E-77	0.927044851	9.44E-79	0.056865496	0.016089653	2.41E-85	0.367117	0.072955149
TIMP2	rs4789855	7.44E-11	0.927128422	5.59E-12	0.06962961	0.003241968	1.54E-16	0.614795	0.072871578
GMPR	rs551027249	1.03E-126	0.927337945	7.51E-128	0.067348176	0.005313879	2.54E-135	0.50339	0.072662055
GMPR	rs573982069	1.03E-126	0.927521838	7.48E-128	0.067163239	0.005314923	2.54E-135	0.50339	0.072478162
GMPR	rs569573520	1.37E-302	0.927525575	9.76E-304	0.066010379	0.006464046	3.16E-308	0.0202717	0.072474425
GMPR	rs140117824	1.03E-126	0.927708171	7.46E-128	0.066975839	0.00531599	2.54E-135	0.50339	0.072291829
CCL3	rs72829980	7.07E-260	0.927925446	5.17E-261	0.067797638	0.004276916	7.07E-268	0.416876	0.072074554
EIF1AD	rs147745496	1.16E-109	0.928087395	4.60E-111	0.03661068	0.035301925	3.09E-118	0.0695409	0.071912605
GMPR	rs62387693	1.38E-302	0.928892218	9.58E-304	0.064625821	0.00648196	3.16E-308	0.0202717	0.071107782
GMPR	rs560499927	1.38E-302	0.929060479	9.56E-304	0.064455653	0.006483868	3.16E-308	0.0202717	0.070939521
HEXB	rs191100885	8.83E-143	0.929093094	5.58E-144	0.058670779	0.012236127	1.97E-151	0.315048	0.070906906
GMPR	rs78204652	4.20E-276	0.929209003	3.06E-277	0.067741926	0.003049071	1.34E-283	0.426424	0.070790997

PTPRU	rs2179795	3.78E-45	0.929272787	1.99E-46	0.049006701	0.021720512	1.13E-51	0.0350841	0.070727213
GMPR	rs530446605	4.20E-276	0.929322986	3.05E-277	0.067627569	0.003049445	1.34E-283	0.426424	0.070677014
TIMP2	rs9894212	7.47E-11	0.930046184	5.35E-12	0.066701645	0.003252171	1.54E-16	0.614795	0.069953816
ADPRHL2	rs182596214	2.47E-122	0.930405511	1.14E-123	0.04315156	0.026442929	5.52E-142	0.406245	0.069594489
MANSC1	rs118164843	1.64E-159	0.930622186	1.12E-160	0.063413515	0.005964299	2.70E-166	0.315876	0.069377814
GMPR	rs180023	1.38E-302	0.931560218	9.21E-304	0.061930633	0.006509149	3.16E-308	0.0202717	0.068439782
GSTM3	rs41302083	2.22E-180	0.931624721	1.35E-181	0.056499289	0.01187599	1.53E-190	0.949302	0.068375279
GMPR	rs180020	1.38E-302	0.931712772	9.16E-304	0.061784901	0.006502327	3.16E-308	0.0202717	0.068287228
PTPRU	rs12402615	3.79E-45	0.931723284	1.89E-46	0.046498927	0.02177779	1.13E-51	0.0350841	0.068276716
MANSC1	rs116855150	1.64E-159	0.932399886	1.08E-160	0.061624423	0.005975692	2.70E-166	0.315876	0.067600114
PNLIP	rs113558089	2.58E-13	0.932935949	1.39E-14	0.050138764	0.016925287	1.24E-19	0.960331	0.067064051
GSTM3	rs139094298	2.23E-180	0.933393185	1.31E-181	0.054708281	0.011898534	1.53E-190	0.949302	0.066606815
GMPR	rs72840248	1.38E-302	0.933506145	8.87E-304	0.05979653	0.006697325	3.16E-308	0.0202717	0.066493855
GMPR	rs182828893	1.40E-302	0.933844085	8.91E-304	0.059485369	0.006670546	3.16E-308	0.0202717	0.066155915
CD59	rs10836121	2.39E-94	0.93389534	1.20E-95	0.047001821	0.019102839	5.10E-101	0.0388168	0.06610466
CD59	rs4756046	2.39E-94	0.933934969	1.20E-95	0.046961382	0.01910365	5.10E-101	0.0388168	0.066065031
CRP	rs145663198	2.06E-71	0.933978836	1.41E-72	0.063600972	0.002420192	8.90E-78	0.760913	0.066021164

CCL3	rs854683	7.12E-260	0.934093013	4.69E-261	0.061601643	0.004305343	7.07E-268	0.416876	0.065906987
PSMB1	rs1011013	4.46E-302	0.934398866	2.49E-303	0.052190729	0.013410405	3.16E-308	0.0332407	0.065601134
CD59	rs831630	2.39E-94	0.934518759	1.19E-95	0.046365649	0.019115591	5.10E-101	0.0388168	0.065481241
PNLIP	rs147414236	2.58E-13	0.935012548	1.33E-14	0.048024238	0.016963213	1.24E-19	0.960331	0.064987452
GMPR	rs2237213	2.25E-302	0.935175784	1.38E-303	0.057470188	0.007354028	3.16E-308	0.0202717	0.064824216
PSAP	rs1867981	4.41E-237	0.935427763	2.93E-238	0.06220549	0.002366747	1.40E-244	0.744017	0.064572237
POFUT1	rs35968884	7.51E-256	0.935483071	3.49E-257	0.043452571	0.021064358	9.43E-266	0.0956269	0.064516929
POFUT1	rs17268666	7.51E-256	0.935483071	3.49E-257	0.043452571	0.021064358	9.43E-266	0.0956269	0.064516929
S100A2	rs75282796	2.65E-21	0.935573715	1.65E-22	0.058160787	0.006265498	3.04E-27	0.813899	0.064426285
GMPR	rs550480982	4.03E-302	0.935584605	2.39E-303	0.055415377	0.009000018	3.16E-308	0.0202717	0.064415395
POMC	rs531061141	0.014324556	0.920650862	0.000784711	0.050420193	0.013819678	5.52E-08	0.434834	0.064239871
POFUT1	rs188411619	7.51E-256	0.935760781	3.46E-257	0.043168608	0.021070611	9.43E-266	0.0956269	0.064239219
ACY1	rs190880385	7.51E-23	0.93605113	3.92E-24	0.048853381	0.015095488	3.38E-29	0.417047	0.06394887
EPHA5	rs28694596	1.33E-05	0.936076621	8.17E-07	0.05734058	0.006568639	1.82E-11	0.3086	0.063909219
S100A2	rs61265945	2.65E-21	0.936566609	1.62E-22	0.057161244	0.006272147	3.04E-27	0.813899	0.063433391
POFUT1	rs192204714	8.55E-187	0.93680286	5.03E-188	0.055145724	0.008051416	1.53E-196	0.558802	0.06319714
FBP2	rs573212	1.27E-49	0.936877929	7.77E-51	0.057399546	0.005722525	3.84E-56	0.377181	0.063122071

GMPR	rs4716082	1.10E-300	0.9368915	5.81E-302	0.049667168	0.013441332	3.16E-308	0.0266901	0.0631085
GMPR	rs190307933	4.71E-302	0.936940834	2.70E-303	0.053776725	0.009282441	3.16E-308	0.0202717	0.063059166
PSMB1	rs180793142	4.48E-302	0.937203774	2.36E-303	0.049345565	0.013450661	3.16E-308	0.0332407	0.062796226
POMC	rs112405047	6.48E-06	0.937279852	3.56E-07	0.051558473	0.011154839	7.20E-12	0.772131	0.062713312
GMPR	rs559171614	4.72E-302	0.937449624	2.71E-303	0.053876343	0.008674033	3.16E-308	0.0202717	0.062550376
CD36	rs6961069	2.86E-82	0.937474869	1.65E-83	0.053986483	0.008538648	4.28E-89	0.102588	0.062525131
POFUT1	rs150282037	8.55E-187	0.937478483	4.97E-188	0.054464294	0.008057223	1.53E-196	0.558802	0.062521517
IL1RL2	rs3917265	1.72E-76	0.937678003	9.96E-78	0.054202207	0.008119789	2.37E-83	0.109487	0.062321997
CD36	rs186727522	3.26E-66	0.937699532	2.07E-67	0.059636033	0.002664435	3.24E-72	0.612066	0.062300468
LAG3	rs149244347	3.58E-45	0.93824214	2.00E-46	0.05243159	0.00932627	7.85E-52	0.0910228	0.06175786
SAT2	rs858523	3.26E-127	0.938624032	1.86E-128	0.053488408	0.00788756	1.65E-133	0.104747	0.061375968
SAT2	rs187079266	3.26E-127	0.938675397	1.85E-128	0.053436611	0.007887992	1.65E-133	0.104747	0.061324603
MANSC1	rs3825259	1.65E-159	0.938890641	9.69E-161	0.055092068	0.006017291	2.70E-166	0.315876	0.061109359
FOLH1	rs61885329	3.14E-57	0.939173038	1.53E-58	0.045781936	0.015045026	8.05E-63	0.0932567	0.060826962
FBP2	rs10761339	1.27E-49	0.939217596	7.45E-51	0.055045588	0.005736816	3.84E-56	0.377181	0.060782404
FOLH1	rs7482844	8.95E-57	0.939368937	4.08E-58	0.04276966	0.017861403	8.05E-63	0.0932567	0.060631063
PNLIP	rs562303688	2.67E-13	0.939465969	1.25E-14	0.044030306	0.016503726	1.24E-19	0.960331	0.060534031

IL1RL2	rs4850995	1.73E-76	0.939621242	9.60E-78	0.052242141	0.008136617	2.37E-83	0.109487	0.060378758
LAG3	rs3782735	3.58E-45	0.93962839	1.95E-46	0.05103156	0.00934005	7.85E-52	0.0910228	0.06037161
LAG3	rs147749635	3.58E-45	0.939636345	1.95E-46	0.051023526	0.009340129	7.85E-52	0.0910228	0.060363655
HMOX1	rs145427664	1.63E-33	0.939878825	8.96E-35	0.051624871	0.008496305	3.05E-42	0.893997	0.060121175
SAT2	rs111498817	3.86E-127	0.939949435	2.15E-128	0.052218139	0.007832426	1.65E-133	0.104747	0.060050565
HMOX1	rs540828131	1.63E-33	0.940121084	8.91E-35	0.051380422	0.008498495	3.05E-42	0.893997	0.059878916
SEMA7A	rs78994380	1.37E-15	0.940140162	8.10E-17	0.055611162	0.004248677	7.09E-22	0.964654	0.059859838
ACY1	rs112965920	3.86E-69	0.940290527	1.90E-70	0.046178839	0.013530634	1.79E-77	0.900115	0.059709473
HMOX1	rs75689278	1.63E-33	0.940503697	8.85E-35	0.050994349	0.008501953	3.05E-42	0.893997	0.059496303
TNFSF8	rs10982299	3.70E-34	0.940520713	2.17E-35	0.055265543	0.004213743	9.71E-41	0.315296	0.059479287
IL1RL2	rs112437560	1.73E-76	0.940543968	9.43E-78	0.051311425	0.008144607	2.37E-83	0.109487	0.059456032
SHBG	rs858519	6.38E-88	0.940584434	3.61E-89	0.053188015	0.006227551	9.52E-95	0.14582	0.059415566
SEMA7A	rs148553967	1.37E-15	0.940616013	8.03E-17	0.05513316	0.004250827	7.09E-22	0.964654	0.059383987
EIF1AD	rs564880527	2.30E-33	0.940661857	1.00E-34	0.041025221	0.018312923	5.50E-40	0.236768	0.059338143
GMPR	rs3828864	4.16E-80	0.941135672	2.30E-81	0.05202548	0.006838848	7.57E-87	0.185104	0.058864328
GMPR	rs545556242	4.17E-301	0.941160991	2.29E-302	0.051593141	0.007245867	3.16E-308	0.0266901	0.058839009
GSTM3	rs536418758	2.79E-43	0.941332777	1.47E-44	0.049464977	0.009202247	6.87E-51	0.254425	0.058667223

MAN1A2	rs12117281	1.20E-103	0.94142003	5.47E-105	0.0427629	0.01581707	7.69E-110	0.0684542	0.05857997
CCL3	rs854469	7.18E-260	0.941961594	4.09E-261	0.053696796	0.004341611	7.07E-268	0.416876	0.058038406
GSTM3	rs143838310	2.79E-43	0.941974142	1.45E-44	0.048817342	0.009208516	6.87E-51	0.254425	0.058025858
SHBG	rs1050541	6.39E-88	0.942019394	3.51E-89	0.051743555	0.006237052	9.52E-95	0.14582	0.057980606
KRT1	rs147540582	8.94E-14	0.94203761	4.52E-15	0.047607589	0.010354801	5.34E-18	0.252168	0.05796239
RAB6B	rs10212397	1.54E-127	0.942129499	8.96E-129	0.054914404	0.002956098	9.44E-135	0.729587	0.057870501
UROD	rs2487446	1.01E-64	0.942279672	5.72E-66	0.0536278	0.004092529	5.32E-71	0.356367	0.057720328
GMPR	rs186281359	4.18E-301	0.94255456	2.23E-302	0.050188844	0.007256596	3.16E-308	0.0266901	0.05744544
PSAP	rs3747867	4.45E-237	0.942564005	2.60E-238	0.055051181	0.002384815	1.40E-244	0.744017	0.057435995
EFNB2	rs72632701	4.81E-09	0.942695685	2.72E-10	0.053358586	0.003945724	6.82E-14	0.483073	0.05730431
KLRB1	rs118021813	7.79E-42	0.942785241	4.49E-43	0.054297423	0.002917336	2.50E-48	0.515795	0.057214759
VEGFA	rs7692	0.000203631	0.94333228	1.10E-05	0.051004234	0.005448844	8.68E-10	0.285744	0.056453078
KRT1	rs641621	3.33E-12	0.94366965	1.88E-13	0.053243828	0.003086522	1.94E-17	0.490201	0.05633035
CD163	rs7303783	1.10E-19	0.943855491	5.78E-21	0.049401288	0.006743221	1.11E-24	0.125214	0.056144509
CD163	rs11054859	1.09E-19	0.944300465	5.61E-21	0.048594493	0.007105042	1.11E-24	0.125214	0.055699535
LAG3	rs11064323	3.60E-45	0.944306809	1.77E-46	0.046306637	0.009386554	7.85E-52	0.0910228	0.055693191
MAN1A2	rs541770553	1.13E-103	0.944354082	4.79E-105	0.04019524	0.015450678	7.69E-110	0.0684542	0.055645918

LDHB	rs74626198	3.08E-14	0.944622995	1.56E-15	0.047883341	0.007493664	1.79E-20	0.427877	0.055377005
LDHB	rs11046111	3.08E-14	0.944729115	1.56E-15	0.047776379	0.007494506	1.79E-20	0.427877	0.055270885
RAB6B	rs550711656	6.65E-83	0.944876566	3.65E-84	0.051940347	0.003183086	7.45E-90	0.575023	0.055123434
SMPDL3A	rs28385609	5.53E-167	0.9449766	2.96E-168	0.050480597	0.004542803	4.14E-174	0.339815	0.0550234
POFUT1	rs77331109	6.53E-60	0.945029591	3.46E-61	0.05009796	0.004872449	3.46E-67	0.602675	0.054970409
UROD	rs12749939	3.78E-209	0.94526116	1.94E-210	0.048537912	0.006200929	1.43E-216	0.189032	0.05473884
CRAT	rs181839197	1.73E-19	0.945531387	9.45E-21	0.051683079	0.002785534	1.18E-25	0.752458	0.054468613
TACO1	rs144926031	0.080393671	0.861958605	0.00345451	0.037021123	0.017172091	1.27E-07	0.767863	0.054193214
TMEM106A	rs184277380	4.71E-15	0.946435453	2.10E-16	0.042058251	0.011506296	3.09E-22	0.26464	0.053564547
MAN1A2	rs73013841	1.13E-103	0.946587889	4.55E-105	0.03789932	0.015512791	7.69E-110	0.0684542	0.053412111
PSAT1	rs183294886	9.01E-46	0.946706602	4.72E-47	0.04963759	0.003655808	2.08E-52	0.481601	0.053293398
SHBG	rs45599134	3.38E-41	0.946789747	1.81E-42	0.050610944	0.002599309	7.51E-48	0.607784	0.053210253
POFUT1	rs189476169	6.55E-60	0.94736207	3.30E-61	0.047753455	0.004884475	3.46E-67	0.602675	0.05263793
PSAT1	rs2277148	9.01E-46	0.947409672	4.66E-47	0.048931805	0.003658523	2.08E-52	0.481601	0.052590328
GMPR	rs184285632	3.74E-107	0.947447331	1.97E-108	0.049778577	0.002774092	2.49E-114	0.972916	0.052552669
KLRB1	rs61916250	2.54E-170	0.947597857	1.26E-171	0.046756369	0.005645774	5.36E-177	0.17547	0.052402143
CD163	rs6488458	1.10E-19	0.94776889	5.28E-21	0.045464953	0.006766157	1.11E-24	0.125214	0.05223111

PTK7	rs148886793	1.53E-18	0.94810494	7.94E-20	0.049026517	0.002868542	1.04E-24	0.645136	0.05189506
CD36	rs6947745	2.89E-82	0.948836944	1.30E-83	0.04252092	0.008642136	4.28E-89	0.102588	0.051163056
BDNF	rs144178847	7.18E-19	0.948911574	3.47E-20	0.045872798	0.005215628	6.10E-24	0.442022	0.051088426
UROD	rs6704115	6.40E-105	0.949610081	3.16E-106	0.04689899	0.003490928	5.95E-112	0.498156	0.050389919
TMEM106A	rs60766100	4.86E-15	0.949735032	1.58E-16	0.030847856	0.019417112	4.25E-22	0.123304	0.050264968
BDNF	rs10501089	7.19E-19	0.950584957	3.34E-20	0.044190217	0.005224826	6.10E-24	0.442022	0.049415043
B4GALT2	rs555187467	8.26E-116	0.950714505	3.55E-117	0.040866607	0.008418888	1.25E-124	0.517639	0.049285495
TNFSF8	rs1006026	1.61E-56	0.950826484	7.94E-58	0.04681306	0.002360456	4.84E-63	0.839143	0.049173516
PTK7	rs3828755	1.54E-18	0.952060149	7.29E-20	0.045059341	0.002880509	1.04E-24	0.645136	0.047939851
CD14	rs77819967	4.60E-94	0.952332403	1.90E-95	0.039354039	0.008313558	2.55E-100	0.128584	0.047667597
PIGR	rs563200113	4.61E-58	0.952736284	2.16E-59	0.044654906	0.00260881	2.20E-64	0.631422	0.047263716
PIGR	rs748844	4.61E-58	0.952990316	2.15E-59	0.044400179	0.002609505	2.20E-64	0.631422	0.047009684
EPB41	rs204074	5.05E-10	0.953808344	2.22E-11	0.041978299	0.004213356	6.16E-15	0.823319	0.046191655
CD8A	rs113014141	8.04E-41	0.953913277	3.35E-42	0.039765878	0.006320845	2.06E-47	0.783316	0.046086723
B4GALT2	rs3762423	5.37E-119	0.954317299	2.09E-120	0.037083616	0.008599084	1.74E-127	0.518153	0.045682701
EPB41	rs12403568	2.09E-05	0.955226441	9.07E-07	0.04152313	0.003228649	1.13E-10	0.56698	0.044751778
KLRB1	rs1135816	2.57E-170	0.955398865	1.04E-171	0.038908883	0.005692252	5.36E-177	0.17547	0.044601135

CD14	rs73791750	2.16E-154	0.958944204	8.40E-156	0.037296598	0.003759198	3.60E-161	0.373746	0.041055796
CD14	rs5744454	2.16E-154	0.959419853	8.29E-156	0.036819084	0.003761063	3.60E-161	0.373746	0.040580147
CD14	rs5744454	3.57E-98	0.959424652	1.37E-99	0.036819273	0.003756075	1.07E-104	0.378643	0.040575348
CD8A	rs3020728	4.98E-301	0.959879319	1.76E-302	0.033921491	0.006199191	3.16E-308	0.197631	0.040120681
CD8A	rs540829421	5.00E-301	0.964364123	1.53E-302	0.029407722	0.006228155	3.16E-308	0.197631	0.035635877
CD14	rs114928388	2.11E-73	0.964479592	6.83E-75	0.031194462	0.004325947	1.06E-79	0.36725	0.035520408
CD14	rs114928388	1.46E-106	0.964555392	4.73E-108	0.031196992	0.004247616	4.52E-113	0.36725	0.035444608
CD8A	rs543932894	5.01E-301	0.965438003	1.47E-302	0.028326907	0.00623509	3.16E-308	0.197631	0.034561997
CD8A	rs115029613	1.04E-166	0.967564225	2.99E-168	0.027797711	0.004638064	7.34E-174	0.627151	0.032435775
RBP4	rs182421163	0.841909616	0.094628415	0.053129123	0.005967207	0.004365639	0.849745	0.000468188	0.010332846

Supplementary Figure 1. Protein-protein interaction network of 199 target plasma proteins.

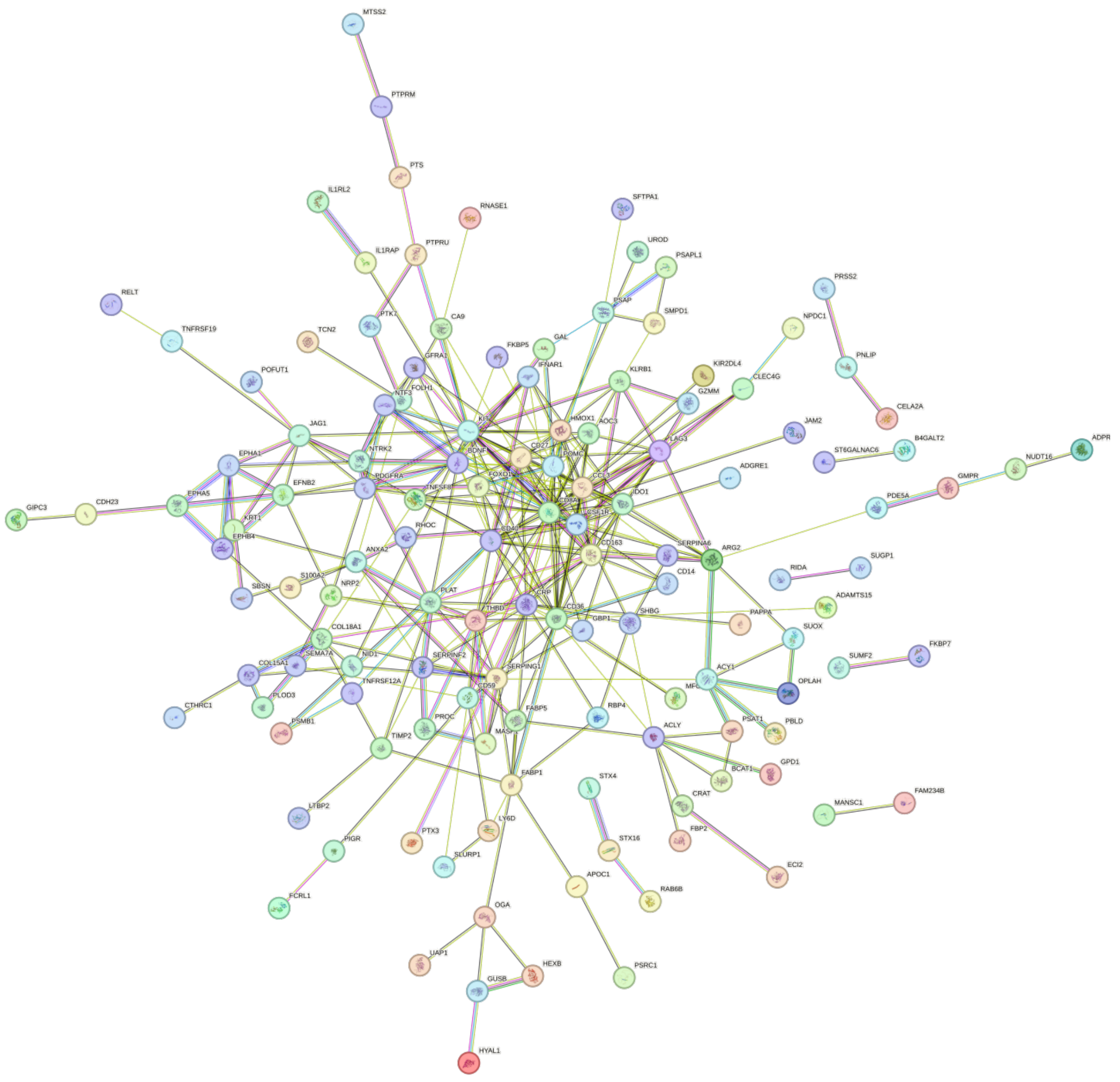

Supplement: Supplementary file 1 — Supplementary figure and tables. [file jcav16p3163s1.pdf]
